# Trends in socioeconomic inequalities in cause-specific premature mortality in Belgium, 1998–2019

**DOI:** 10.1186/s12889-024-17933-z

**Published:** 2024-02-14

**Authors:** Martina Otavova, Bruno Masquelier, Christel Faes, Laura van den Borre, Bram Vandeninden, Eva de Clercq, Brecht Devleesschauwer

**Affiliations:** 1 Center for Demographic Research, UCLouvain, Louvain-la-Neuve, Belgium; 2 Data Science Institute, I-BioStat, Hasselt University, Hasselt, Belgium; 3 Department of Epidemiology and Public Health, Sciensano, Brussels, Belgium; 4 Interface Demography, Department of Sociology, Vrije Universiteit Brussels, Brussels, Belgium; 5 Research Centre on Environmental and Occupational Health, School of Public Health, Université Libre de Bruxelles, Brussels, Belgium; 6 Department of Risk and Health Impact Assessment, Sciensano, Brussels, Belgium; 7 Department of Translational Physiology, Infectiology and Public Health, Ghent University, Merelbeke, Belgium

**Keywords:** Premature mortality, Belgium, Area-based measure of inequality, Belgian Indices of Multiple deprivation, Causes of death

## Abstract

**Background:**

Higher levels of socioeconomic deprivation have been consistently associated with increased risk of premature mortality, but a detailed analysis by causes of death is lacking in Belgium. We aim to investigate the association between area deprivation and all-cause and cause-specific premature mortality in Belgium over the period 1998–2019.

**Methods:**

We used the 2001 and 2011 Belgian Indices of Multiple Deprivation to assign statistical sectors, the smallest geographical units in the country, into deprivation deciles. All-cause and cause-specific premature mortality rates, population attributable fraction, and potential years of life lost due to inequality were estimated by period, sex, and deprivation deciles.

**Results:**

Men and women living in the most deprived areas were 1.96 and 1.78 times more likely to die prematurely compared to those living in the least deprived areas over the period under study (1998–2019). About 28% of all premature deaths could be attributed to socioeconomic inequality and about 30% of potential years of life lost would be averted if the whole population of Belgium faced the premature mortality rates of the least deprived areas.

**Conclusion:**

Premature mortality rates have declined over time, but inequality has increased due to a faster pace of decrease in the least deprived areas compared to the most deprived areas. As the causes of death related to poor lifestyle choices contribute the most to the inequality gap, more effective, country-level interventions should be put in place to target segments of the population living in the most deprived areas as they are facing disproportionately high risks of dying.

**Supplementary Information:**

The online version contains supplementary material available at 10.1186/s12889-024-17933-z.

## Introduction

Social justice is an essential factor that profoundly impacts our lives and determines our well-being, susceptibility to illnesses, and risk of premature death. The poorest of the poor tend to experience the greatest levels of illness and premature mortality, but a social gradient is evident across most countries, where lower socioeconomic positions correlate with poorer well-being [1].

The association of health and mortality with socioeconomic status has been demonstrated worldwide [2–7]. Studies of changes in health inequalities over recent decades have shown a widening or persistence in the difference between rich and poor [8–11]. For instance, Mackenbach et al. (2003) unveiled an increase in relative inequalities in total mortality in Finland, Sweden, Norway, Denmark, England/Wales, and Italy in the period 1990–2010 [8]. A study from Canada reported that premature mortality attributable to inequality increased in the years 1995–2005, and about 40% of premature deaths were attributable to deprivation [12]. A more recent study by Lewer et al. (2020) reported that premature mortality rates in England have increased between 2003 and 2018, particularly for women living in the most deprived areas [4].

In Belgium, Renard et al. (2014) studied regional disparities in premature mortality in 1993–2009 and reported an impressive improvement in premature mortality from the leading causes of death in men and women, but also substantial disparities between the regions [13]. In another study, Renard et al. (2016) used the level of education as a proxy of socioeconomic status (SES) and confirmed a reduction in premature mortality in men and women in the period of 1991–2001, and an increase in relative inequalities in both sexes [14]. Eggerickx et al. (2018) studied the evolution of social differences in mortality in Belgium between 1991 and 2016. They used a multidimensional indicator of deprivation at the individual level and observed a significant increase in social inequalities since the 1990s [15]. In 2021, our team published a study in which we used housing conditions as concrete manifestations of socioeconomic deprivation in Belgium and investigated their association with mortality. Our results showed a decrease in mortality rates but a rise in inequality from 1991 to 2020 [16].

Building on this work, the main objective of this study is to assess the overall magnitude of socioeconomic inequality in all-cause and cause-specific premature mortality between the years 1998 and 2019 in Belgium. The novelty of our study is the use of the Belgian Indices of Multiple Deprivation, measuring the aggregate scale of socioeconomic inequality at the level of the smallest administrative unit in Belgium, the statistical sector [17]. By using health metrics, such as premature mortality rates, population attributable fractions, and potential years of life lost, we introduce the scale of the socioeconomic inequality in Belgium in a way that is easy to communicate to policymakers and the public; track health inequality developments over time; and disaggregate the inequality by sex, level of deprivation, and cause of death.

## Methods

### Belgian Indices of Multiple Deprivation 2001 and 2011 (BIMD2001 and BIMD2011)

**Fig. 1:**
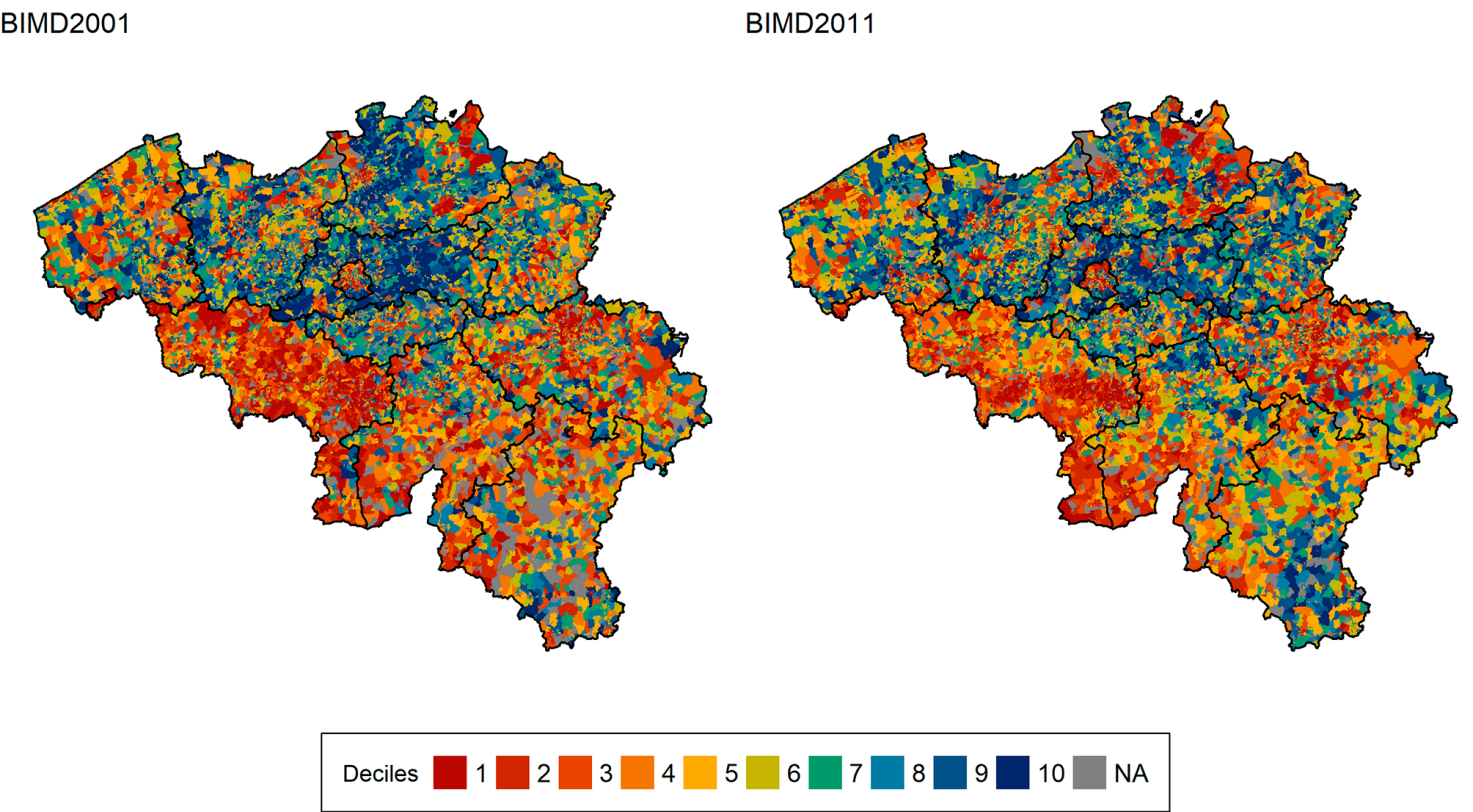
Distribution of the BIMD2001 and BIMD2011 deprivation deciles across Belgian statistical sectors in 2001 and 2011. The most deprived statistical sectors fall into the first deprivation decile (dark red)

The Belgian Indices of Multiple Deprivation (BIMDs) are time- and spatial-specific tools for measuring multiple deprivation at the level of the statistical sector [17]. The BIMDs encompass six domains of deprivation: income, education, employment, housing, health and crime. These domains are combined to obtain a single score for each geographical area. The scores are then ranked and assigned to deprivation deciles from the most (1) to the least (10) deprived. Figure 1 shows the distribution of deprivation deciles in 2001 and 2011. To avoid correlation between the indices with the health domain and the health outcomes, we re-built the BIMDs without the health domains for this study. The BIMDs are available via https://github.com/bimd-project/bimd, the interactive tool can be found at https://bimd.sciensano.be/tool and more details on the methodology are found in the paper by Otavova et al. (2023) [17].

Due to concerns related to privacy and the unreliability of small population estimates, we excluded from our analysis all statistical sectors with 10 or fewer inhabitants in the year 2001 and 2011, equaling to 1,486 (7.5%) and 1,018 (5.2%) statistical sectors, out of which 50.7% (2001) and 36.5% (2011) had zero inhabitants and corresponded to forest, parks, rivers, etc… The total amount of statistical sectors used in our study was therefore 18,295 (in 2001) and 18,764 (in 2011). These statistical sectors are heterogeneous in population size, with a median population of 344 (1st quartile of 133 and 3rd quartile of 759) in 2001 and 347 (1st quartile of 130 and 3rd quartile of 781) in 2011.

The BIMDs were used to categorize deaths and population exposure by deprivation level based on the place of residence at the time of death. We used the BIMD2001 for the periods 1998–2003 and 2004–2008, and the BIMD2011 for the periods 2009–2013 and 2014–2019.

### Data

We used pseudonymized individual-level all-cause and cause-specific mortality data extracted from the Civil Registry in Belgium relative to deaths that occurred between Jan. 1, 1998, and Dec. 31, 2019 in people aged 1 to 74 years. Infant deaths were excluded from the present study due two reasons. First, an infant death is officially registered in the National Register (our data source) only when an infant lives through the January 1st. Including the age 0 in our analysis might result in underestimating the infant mortality. Additionally, infant mortality arises from factors that are specific to this age group, and there are more suitable indicators available that better capture mortality within this age group [18].

Data included age, sex, the underlying cause of death by International Classification of Diseases-10 code, and a place of residence of the deceased, defined by the statistical sector. Mid-year population estimates by statistical sector, sex, and single-year of age from 1998 to 2019 were obtained from the National Register data, which includes all legal residents of Belgium and excludes irregular migrants and asylum seekers.

### Grouping of the causes of deaths

The causes of deaths were grouped using a three-level hierarchy. First, we subset all-cause and all-age deaths to premature deaths (deaths before age of 75), and then we grouped them by ICD-10 chapters (such as I00-I99: circulatory diseases). Second, if a chapter included subcategories responsible for more than 1,000 deaths between 1998 and 2019 (such as I20-I25: ischaemic heart disease), we created a second level showing these subcategories. Finally, if the subcategory included any three-digit diagnosis responsible for more than 1,000 deaths (such as I20: acute myocardial infarction), we created a third level for these diagnoses. Although the threshold of 1,000 deaths was chosen arbitrarily, it corresponded to death counts that were large enough to ensure reliable estimates.

### Age-standardized premature mortality rates

Premature mortality was defined as mortality occurring before the age of 75. The threshold of 75 years was chosen for two main reasons. First, the officially reported causes of deaths after the age of 75 are less reliable because of more frequent competing causes of death in older people [19]. Second, such upper-limit is consistent with the recent definition of avoidable mortality [20].

We stratified the overall and cause-specific mortality and population data to 5-year age groups (except the lowest age group made of children aged 1–4), sex, BIMDs’ deprivation deciles, and periods 1998–2003, 2004–2008, 2009–2013, and 2014–2019. We then computed sex- and age-standardized premature mortality rates per 100,000 person-years for each stratum using the sex and age structure of the European standard population 2018.

In addition, a z-test was performed to assess the statistical difference in ASMR between two compared populations. The evaluation between the first and last period and between the most and least deprived deciles is expressed in absolute and relative differences; e.g., (Rate 4th– Rate 1st )/rate 1st.

More on the computational approach is included in Supplementary materials.

### Population attributable fraction

We computed the population attributable fraction (PAF) to measure the impact of the inequality in the whole population, and to suggest the percentage of improvement that could be expected, if the whole of Belgium had the premature mortality rates of the least deprived areas. Within each age group, sex, and period, the premature mortality rates of the least deprived decile were used as reference groups and applied to remaining deciles to produce a number of expected deaths under this counterfactual scenario. The mortality attributable to socioeconomic inequality represents the difference between the observed and expected deaths.

A Monte Carlo simulation approach was used to estimate the 95% uncertainty interval (UI). Specifically, we performed 10,000 iterations of random sampling of deaths in each sex, period, decile, and cause of death. The sampling was carried out from a Poisson distribution, with the mean number of deaths serving as the distribution parameter, determined from the observed data. Death rates were computed by dividing the simulated deaths by the corresponding populations at risk, and the death rates of the least deprived decile were then applied to the corresponding population at risk in each stratum to compute the expected number of deaths. Attributable deaths were determined by taking the difference between simulated deaths and expected deaths. The sum of attributable deaths was calculated across all iterations of the Monte Carlo simulation, and the PAF, the ratio of attributable deaths to the total simulated deaths, was computed. The 95% UI for the PAF was obtained by computing quantiles of the PAF distribution.

### Potential years of life lost

The potential years of life lost (PYLL) is a summary measure of premature mortality that estimates the years of potential life lost due to premature deaths. We computed crude PYLL for all-cause and cause-specific mortality, stratified by sex, period, and deprivation decile. The number of PYLL was calculated in each stratum by summing the number of deaths at each age between 1 and 74 years, multiplied by the number of years of life remaining up to the 75th birthday [21]. The PYLL due to inequality was then computed as an absolute and relative difference between the PYLL in the least deprived decile and the remaining deciles. We report the uncorrected number of PYLL due to our interest in the actual number of deaths distributed by age, sex, and cause, not in the number of deaths in the theoretical standard population obtained by multiplying the specific death rates by the standard population.

In addition, we calculated the age-standardized PYLL per 100,000 persons using the European Standard Population 2018, stratified by sex, period, and deprivation decile. These were introduced to facilitate comparisons between different areas, specifically, between the most and least deprived.

All analyses were done in R version 4.1.1 [22].

## Results

### All-cause and cause-specific premature mortality rates, their disparities in time and across deprivation deciles

Table 1 presents age-standardized premature mortality rates by sex, deprivation decile and period (1998–2003, 2004–2008, 2009–2013, and 2014–2019). Over the whole period, the overall premature mortality rate in men and women living in areas considered as the most deprived was 745 (95% UI 741–749) and 381 (95% UI 378–384) per 100,000 person-years, respectively. Premature mortality rates were significantly lower in men and women living in the least deprived areas, estimated at 380 (95% UI 378–384) and 213 (95% UI 210–216) per 100,000 person-years. The ratio of the most/least deprived premature mortality rates in men (1.97; 95% UI 1.96–1.98) differed significantly from the ratio in women (1.79; 95% UI 1.78–1.79).

**Table 1:** Standardized premature mortality rates per 100,000 person-years

	1998–2003	2004–2008	2009–2013	2014–2019	All years	Absolute change in time	Relative change in time
Men							
Most deprived	865 (857–874)	775 (767–784)	711 (703–718)	648 (641–654)	745 (741–749)	-217	-25.09%
Least deprived	475 (467–482)	397 (389–405)	343 (335–351)	301 (295–308)	380 (378–384)	-175	-36.63%
Absolute difference	390	378	367	347	365		
Relative difference	82.10%	95.21%	107.29%	115.28%	96.05%		
Women							
Most deprived	414 (408–420)	389 (383–395)	374 (369–380)	350 (345–355)	381 (378–384)	-64	-15.47%
Least deprived	247 (241–253)	216 (211–222)	203 (197–209)	184 (179–189)	213 (210–216)	-63	-25.51%
Absolute difference	167	173	172	166	153		
Relative difference	67.61%	80.10%	84.73%	90.21%	71.83%		

Premature mortality rates are standardized using the European Standard Population 2018. Rates for other deciles are not shown for brevity, and lie monotonically between these values. Change = a relative change in values from 1998 to 2003 to 2014–2019. Most deprived and least deprived refer to the 1st and 10th deciles as measured in 1998–2003 and 2004–2008 with the BIMD2001; and in 2009–2013 and 2014–2019 with the BIMD2011

Comparing 1998–2003 with 2014–2019, the overall premature mortality rates decreased over time in both sexes and across all deciles. The relative difference in the most and least deprived deciles over time was greater in men (25.09% and 36.63%) than in women (15.47% and 25.51%). The relative difference in premature mortality between the most and least deprived deciles increased in men (1998–2003; 82.10% and 2014–2019; 115.28%) as well as in women (1998–2003; 67.61% and 2014–2019; 90.21%). In 1998–2003, mortality rates of men and women living in the most deprived areas were, respectively, 1.82 (95% UI 1.79–1.85) and 1.68 (95% UI 1.64–1.72) higher than in those living in the least deprived areas. In 2014–2019, these ratios increased to 2.15 (95% UI 2.10–2.20) in men and 1.90 (95% UI 1.85–1.96) in women.

**Fig. 2:**
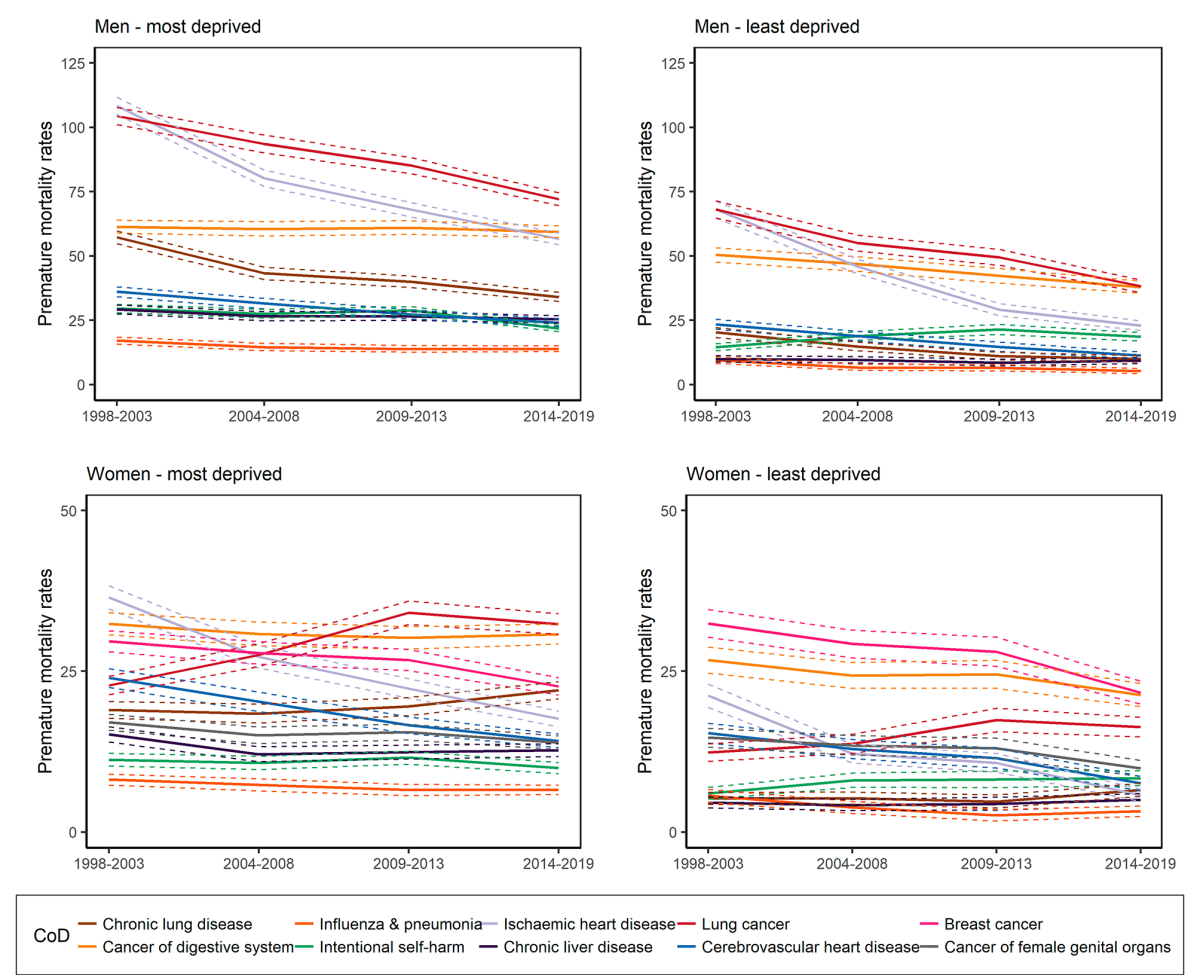
Causes of death (CoD) with the highest rates of premature mortality and their development over time for men and women living in the most and least deprived areas

Important differences were observed in cause-specific premature mortality in men and women living in areas identified as most and least deprived by the BIMDs. We ranked the causes of deaths according to the male and female premature mortality rates. The top 10 causes of deaths for each sex as ranked in the first period (1998–2003) with their premature mortality rates in the most and least deprived are displayed in Fig. 2; Table 2.

**Table 2:** Top 10 causes of death of premature death (before 75 years of age) in women and their evolution over time in most and least deprived areas in Belgium (1998–2019)

	Period	ASMR (D_1_)	ASMR (D_10_)	Absolute difference	Relative difference	ASMR RATIO (95%CI)	*p*-value (ASMR RATIO)
Premature mortality (women)	1998–2003	413.95 (408.20-419.71)	246.97 (241.43-252.51)	166.98	67.61%	1.68 (1.64–1.72)	< 0.001
	2014–2019	349.91 (345.08-354.74)	183.86 (178.50-189.21)	166.02	90.31%	1.90 (1.85–1.96)	< 0.001
	Absolute difference	-64.04	-63.11				
	Relative difference	-15.47	-25.55%				
Ischemic heart disease	1998–2003	36.50 (34.72–38.29)	21.20 (19.39–23.01)	15.30	72.17%	1.72 (1.57–1.88)	< 0.001
	2014–2019	17.59 (16.40-18.78)	5.72 (4.79–6.64)	11.87	207.52%	3.08 (2.67–3.55)	< 0.001
	Absolute difference	-18.91	-15.48				
	Relative difference	-51.8	-73.0%				
Neoplasm of digestive system	1998–2003	32.40 (30.70–34.10)	26.75 (24.72–28.77)	5.65	21.12%	1.21 (1.10–1.32)	< 0.001
	2014–2019	30.77 (29.20-32.34)	21.30 (19.52–23.08)	9.47	44.46%	1.44 (1.32–1.58)	< 0.001
	Absolute difference	-1.63	-5.45				
	Relative difference	-5.10	-20.40%				
Breast cancer	1998–2003	29.64 (28.01–31.28)	32.45 (30.30–34.60)	2.81	8.65%	0.91 (0.84–0.99)	< 0.001
	2014–2019	22.65 (21.32–23.99)	21.66 (19.88–23.44)	0.99	4.57%	1.05 (0.95–1.16)	< 0.001
	Absolute difference	-6.99	-10.79				
	Relative difference	-23.58	-33.25%				
Lung cancer	1998–2003	22.79 (21.35–24.23)	12.36 (11.00-13.73)	10.43	84.39%	1.84 (1.64–2.09)	< 0.001
	2014–2019	32.35 (30.76–33.95)	16.32 (14.78–17.86)	16.03	98.22%	1.98 (1.80–2.18)	< 0.001
	Absolute difference	9.56	3.96				
	Relative difference	41.94%	32.04%				
Cerebrovascular heart disease (Stroke)	1998–2003	23.97 (22.52–25.43)	15.39 (13.84–16.93)	8.58	55.75%	1.84 (1.64–2.09)	< 0.001
	2014–2019	14.17 (13.10-15.25)	7.58 (6.46–8.69)	6.59	86.95%	1.98 (1.80–2.18)	< 0.001
	Absolute difference	-9.8	-7.81				
	Relative difference	-40.88	-50.74%				
Chronic lung disease (COPD + Asthma)	1998–2003	18.99 (17.69–20.29)	5.2 (4.27–6.14)	13.79	265.19%	3.65 (3.14–4.28)	< 0.001
	2014–2019	22.05 (20.72–23.39)	6.57 (5.55–7.59)	15.48	235.61%	3.36 (2.94–3.83)	< 0.001
	Absolute difference	3.06	1.37				
	Relative difference	16.11%	26.34%				
Female genital organs cancer	1998–2003	17.05 (15.81–18.29)	14.69 (13.22–16.15)	2.36	16.06%	1.16 (1.03–1.28)	< 0.001
	2014–2019	13.91 (12.87–14.96)	9.91 (8.69–11.14)	4	40.36%	1.40 (1.22–1.61)	< 0.001
	Absolute difference	-3.14	-4.78				
	Relative difference	-18.42	-32.53%				
Chronic liver disease	1998–2003	15.18 (13.99–16.37)	4.59 (3.74–5.45)	2.31	230.72%	3.30 (2.80–3.85)	< 0.001
	2014–2019	12.74 (11.74–13.73)	5.02 (4.10–5.93)	7.72	153.78%	2.54 (2.16–2.98)	< 0.001
	Absolute difference	-2.44	0.43				
	Relative difference	-16.07%	9.37%				
Intentional self-harm	1998–2003	11.23 (10.24–12.21)	6.02 (5.09–6.95)	5.21	86.54%	1.86 (1.58–2.22)	< 0.001
	2014–2019	9.94 (9.09–10.79)	8.35 (7.18–9.51)	1.59	19.04%	1.19 (1.02–1.39)	< 0.001
	Absolute difference	-1.29	2.33				
	Relative difference	-11.48%	38.70				
Influenza & Pneumonia	1998–2003	8.16 (7.29–9.04)	5.65 (4.62–6.67)	2.51	44.43%	1.45 (1.19–1.70)	< 0.001
	2014–2019	6.57 (5.83–7.31)	3.24 (2.45–4.02)	3.33	102.78%	2.0 (1.61–2.55)	< 0.001
	Absolute difference	-1.59	-2.41				
	Relative difference	-19.49	-42.65%				

In men and women across all causes of deaths, the most deprived areas had greater premature mortality rates than the least deprived areas. For most causes of death, we observed a reduction in cause-specific premature mortality in the most and least deprived decile in 2014–2019 compared to 1998–2003. This reduction was always greater in the least than most deprived areas, but rates of decline varied by cause of death. For the majority of causes of deaths in men and women, the relative difference between the most and least deprived areas was greater in the last period under observation (2014–2019), due to slower decrease in premature mortality in men living in the most deprived, compared to those living in the least deprived areas. For instance, the premature mortality of men caused by neoplasms decreased by 22.01% in the most deprived groups (2014–2019 vs. 1998–2003), and by 32.38% in the least deprived group. As a result, the gap between the most and least deprived in 1998–2003 and 2014–2019 increased, from 42.02 to 63.82%. Similar results were observed in premature mortality rates of neoplasms in women, greater reduction in least deprived areas (20.29% vs. 5.76%) and an increase in the gap between the most and least deprived areas. In 2014–2019, women living in the most deprived areas had 41.17% greater premature mortality rates caused by neoplasms compared to women living in the least deprived areas, whereas in 1998–2003 this difference equaled to 19.40%.

For a few causes of death, we observed increases in premature mortality risks over time, in both the most and least deprived areas. In women, mortality rates of lung cancer in the most and least deprived areas increased by 41.94% and 32.04% in 2014–2019 vs. 1998–2003, whereas mortality rates of chronic lung disease (COPD) increased by 16.11% and 26.34% (Fig. 2). Mortality increases were also observed when deaths were caused by chronic diseases and conditions related to unhealthy lifestyle behavior, such as drugs and alcohol. For instance, the proportion of deaths caused by drugs or alcohol poisoning doubled and tripled in the most and least deprived deciles.

Another pattern worth mentioning was that some causes of death showed a reduction in the premature mortality rates in the most deprived areas, but an increase in the least deprived areas (1998–2003 vs. 2014–2019). For instance, mortality from intentional self-harm demonstrated a decrease by -26.44% and − 11.48% among men and women living in the most deprived areas and an increase by 28.00% and 38.70% among men and women in the least deprived areas (1998–2003 vs. 2014–2019).

Age standardized mortality rate ratios (ASMR RATIO) of the most/the least deprived premature mortality rates were computed for each category of death in the period 1998–2003 and 2014–2019. A majority of causes of death had the ASMR ratio significantly greater in the last period compared to the first period, suggesting that health inequalities have amplified. Some causes demonstrated however a significant decrease in inequality. This was the case of psychoactive substance use by men, and intentional self-harm in men and women (Tables 3 and 2).

**Table 3:** Top 10 causes of death of premature death (before 75 years of age) in men and their evolution over time in most and least deprived areas in Belgium (1998–2019)

	Period	ASMR (D_1_)	ASMR (D_10_)	Absolute difference	Relative difference	ASMR RATIO (95%CI)	*p*-value (ASMR RATIO)
Premature mortality (men)	1998–2003	865.72 (857.4-874.03)	474.83 (467.19-482.47)	390.89	82.32%	1.82 (1.79–1.85)	< 0.001
	2014–2019	647.80 (641.35-654.26)	301.79 (294.95-308.63)	346.01	114.65%	2.15 (2.10–2.20)	< 0.001
	Absolute difference	-217.92	-173.04				
	Relative difference	-25.17	-36.44%				
Ischemic heart disease	1998–2003	108.34 (104.98-111.72)	68.05 (64.74–71.35)	40.30	59.22%	1.59 (1.51–1.65)	< 0.001
	2014–2019	56.60 (54.37–58.83)	22.86 (21.00-24.71)	33.74	147.60%	2.48 (2.29–2.68)	< 0.001
	Absolute difference	-51.74	-45.19				
	Relative difference	-47.76	-66.41%				
Lung cancer	1998–2003	104.41 (101.09-107.73)	68.07 (64.74–71.38)	36.34	53.38%	1.53 (1.45–1.60)	< 0.001
	2014–2019	72.06 (69.54–74.58)	38.20 (35.81–40.60)	33.85	88.61%	1.89 (1.77–2.01)	< 0.001
	Absolute difference	-32.35	-29.87				
	Relative difference	-30.99	-43.88%				
Neoplasm of digestive system	1998–2003	61.34 (58.79–63.88)	50.39 (47.57–53.21)	10.95	21.73%	1.21 (1.14–1.29)	< 0.001
	2014–2019	59.36 (57.07–61.65)	37.86 (35.49–40.23)	21.50	56.81%	1.57 (1.46–1.68)	< 0.001
	Absolute difference	-1.98	-12.53				
	Relative difference	-3.22	-24.86%				
Chronic lung disease	1998–2003	57.20 (54.72–59.67)	20.27 (18.40-22.15)	36.93	182.19%	2.82 (2.59–2.99)	< 0.001
	2014–2019	34.07 (32.30-35.85)	9.97 (8.72–11.22)	24.10	241.73%	3.42 (3.06–3.82)	< 0.001
	Absolute difference	-23.13	-10.30				
	Relative difference	-40.44	-50.81%				
Accidents	1998–2003	36.75 (34.99–38.51)	17.27 (15.77–18.76)	19.48	112.79%	2.12 (1.95–2.31)	< 0.001
	2014–2019	33.32 (31.75–34.89)	13.79 (12.31–15.27)	19.53	141.62%	2.42 (2.19–2.66)	< 0.001
	Absolute difference	-3.43	-3.48				
	Relative difference	-9.3	-20.15%				
Cerebrovascular heart disease (Stroke)	1998–2003	36.08 (34.12–38.03)	23.46 (21.49–25.43)	12.62	53.79%	1.54 (1.40–1.65)	< 0.001
	2014–2019	23.98 (21.49–25.43)	11.34 (9.98–12.7)	12.64	111.46%	2.11 (1.88–2.38)	< 0.001
	Absolute difference	-12.10	-12.12				
	Relative difference	-33.54	-51.66%				
Chronic liver disease	1998–2003	29.23 (27.53–30.93)	9.98 (8.76–11.20)	19.25	192.88%	2.93 (2.61–3.25)	< 0.001
	2014–2019	25.34 (23.91–26.77)	9.29 (8.13–10.45)	16.05	172.76%	2.73 (2.43–3.06)	< 0.001
	Absolute difference	-3.89	-0.69				
	Relative difference	-13.30%	-6.91%				
Intentional self-harm	1998–2003	29.61 (28.03–31.20)	14.50 (13.14–15.85)	15.11	104.21%	2.04 (1.85–2.25)	< 0.001
	2014–2019	21.78 (20.55-23.00)	18.56 (16.88–20.24)	3.22	17.35%	1.17 (1.06–1.30)	< 0.001
	Absolute difference	-7.83	4.06				
	Relative difference	-26.44%	28.00%				
Influenza & Pneumonia	1998–2003	17.06 (15.72–18.41)	9.49 (8.17–10.80)	7.57	79.78%	1.80 (1.55–2.02)	< 0.001
	2014–2019	13.93 (12.80-15.06)	5.26 (4.29–6.22)	8.67	164.82%	2.65 (2.24–3.13)	< 0.001
	Absolute difference	-3.13	-4.23				
	Relative difference	-18.35	-44.57%				
Neoplasm of urinary system	1998–2003	14.56 (13.31–15.82)	12.51 (11.08–13.93)	2.05	16.39%	1.16 (1.01–1.31)	< 0.001
	2014–2019	12.29 (11.23–13.36)	9.32 (8.11–10.54)	2.97	31.87%	1.32 (1.13–1.53)	< 0.001
	Absolute difference	-2.27	-3.19				
	Relative difference	-15.60	-25.49%				

The detailed analysis of inequalities in cause-specific premature mortality across age groups, measured by the ASMR ratio, revealed that the magnitude and location differ by cause of death, sex, and period studied. For several causes of death, the inequality has moved toward the higher ages over time, but their peaks were more pronounced in 1998–2003, compared to 2014–2019. This trend was observed for instance in male deaths caused by chronic lung disease and chronic liver disease with their highest points of inequality moving from the age group of 50–54 to 65–69, and from 40 to 44 to 50–54 during the twenty years under study. In women, the same trend was observed in deaths by chronic liver disease or intentional self-harm, for which the peak of inequality moved from the age group of 45–49 to 50–54, and from 25 to 29 to 35–39. On the contrary, some causes of death showed greater inequality peak at higher ages spreading across several age groups in the last period compared to the first period studied. This trend was observed in deaths by cancer of the digestive or respiratory system, stroke, or accident in men; in deaths by breast cancer in women; and deaths by ischaemic heart disease in both.

Lastly, for several causes of death, the level of inequality in the first period studied was consistently higher across all ages compared to the last period, such as hypertension or certain types of cancer (cancer of mouth, lymphoid, bone and articular cartilage), in both, men and women. A more comprehensive overview of cause-specific premature mortality rates in men and women is provided in Tables S2 and S3 in Supplementary materials.

### Overall and cause-specific premature mortality attributable to socioeconomic inequality in Belgium

We computed the proportion (PAF) of premature mortality attributable to socio-economic inequality for both all-cause and cause-specific premature mortality. Of the 789,666 premature deaths that occurred in Belgium between 1998 and 2019, 27.6% (95% UI 27.1–28.2) were attributable to socioeconomic inequality. The PAF was greater in men (29.1%, 95% UI 28.4–29.8) compared to women (25.1%, 95% UI 24.2–26.1).

Our results also showed an increase in premature mortality attributable to inequality over time (Table 4). The overall PAF has increased from 24.2% (95% UI 23.1–25.2) in 1998–2003 up to 32.6% (95% UI 31.5–33.7) in 2014–2019. Sex differences were observed in PAF development over time. In 1998–2003 and 2014–2019, premature mortality attributable to SE inequality was greater in men (25.5%, 95% UI 24.2–26.8; 34.2%, 95% UI 32.8–35.6) than in women (21.8%, 95% UI 19.9–23.6; 29.9%, 95% UI 28.1–31.9). These numbers, however, suggested similar increase in women (8.2%) as in men (8.6%). In 1998–2019, the greatest proportion of overall premature deaths attributable to inequality occurred in men aged 40–59 years (around 60%), and in women aged 40–54 years (more than 50%), living in the most deprived areas, respectively (Figure S4 in Supplementary materials).

**Table 4:** Population attributable fraction of premature mortality associated with socioeconomic inequality by sex and period and 95% UI

	1998–2003	2004–2008	2009–2013	2014–2019		All years	Relative difference between 1st and 4th period
Men	25.5 (24.2–26.8)	28.0 (26.6–29.5)	31.9 (30.4–33.5)	34.2 (32.8–35.6)	29.1 (28.4–29.8)		34.04%
Women	21.8 (19.9–23.6)	24.2 (22.2–26.2)	26.4 (24.2–28.5)	29.9 (28.1–31.9)	25.1 (24.2–26.1)		26.23%
Absolute difference	3.75	3.81	5.52	4.20	4.00		

**Fig. 3:**
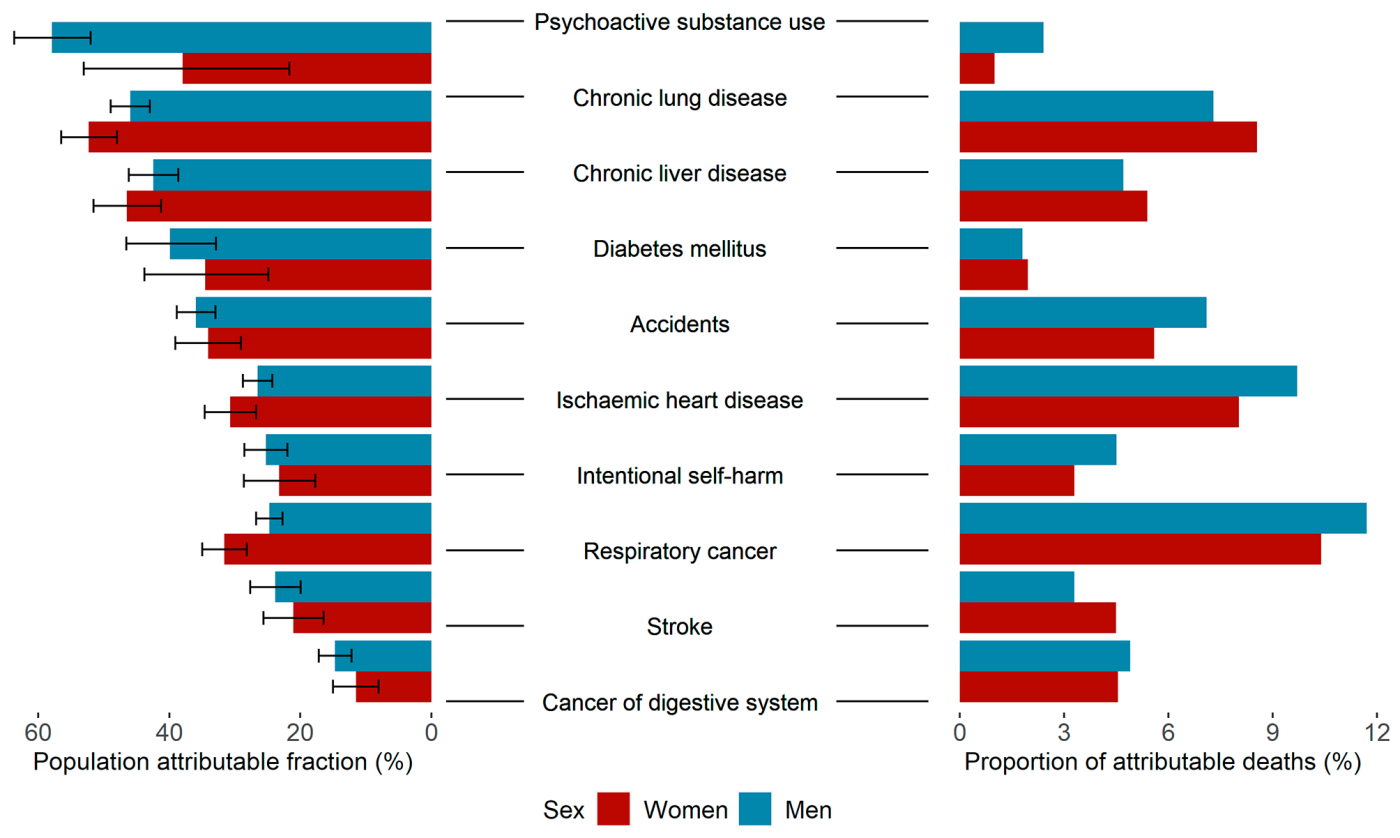
Causes of death with the greatest proportion of premature mortality attributable to socioeconomic inequality and their contributions to the total number of attributable deaths, 1998–2019

Considering the leading causes of death, the greatest inequality was observed for deaths due to psychoactive substance use (50.9%, 95% UI 44.9–56.8), chronic lung disease (47%, 95% UI 44.5–49.5), and chronic liver disease (43%, 95% UI 40-46.2). Almost half of these deaths were attributable to inequality. External causes of deaths, such as deaths due to accidents (33.7%, 95% UI 31.1–36.3) showed high inequalities as well. Almost one fourth of the deaths caused by diseases of the circulatory system could be attributable to inequality, with leading causes of ischaemic heart diseases (26%, 95% UI 24–28) and cerebrovascular heart disease or stroke (22%, 95% UI 19.2–25.1). Causes of death with the greatest proportion of premature mortality attributable to inequality are shown in Fig. 3.

Neoplasms generally have low inequality, but neoplasms of liver and intrahepatic bile ducts (28.1%, 95% UI 22.6–33.5), neoplasms of mouth (26.4%, 95% UI 20.5–32.1), lung (25%, 95% UI 22.6–26.2) and larynx (23.3%, 95% UI 14.5–32.6) were exceptions, with higher proportions of deaths attributable to inequality. The contribution of considered diseases to the total number of attributable deaths, was greatest for lung cancer, ischaemic heart disease, and chronic lung diseases, equaling about 10%.

Our results showed only a few significant differences in PAF of major ICD10 chapters in men and women and across periods. In men, circulatory diseases, neoplasms and respiratory diseases increased by 15%, 8% and 10% in 4th period under study, compared to the 1st period. In women, only circulatory diseases and neoplasms showed an increase by 16% and 10% in premature mortality attributable to inequality over time. Across all major causes of deaths, the middle-aged men and women in the most deprived groups had the greatest proportion of premature mortality attributable to inequality.

The contribution of diseases to the total number of attributable deaths was similar for men and women across all periods under study. Table S5 in Supplementary materials shows a detailed overview of the cause-specific PAF.

### Crude PYLL due inequality in Belgium since 1998

In the period of 1998–2019, a total number of 11,221,841 potential years of life (PYLL) were lost in Belgium, about 64.5% (7,243,567) in men and 35.5% (3,978,274) in women. Using the deprivation deciles, we computed the expected number of PYLL in Belgian men (4,899,798) and women (2,892,879), if all had the same mortality rates of those living in the least deprived areas. Potential years of life lost due inequality represented 32.3% (2,343,769) of all PYLL in men and 27.3% (1,085,395) of all PYLL in women.

**Fig. 4:**
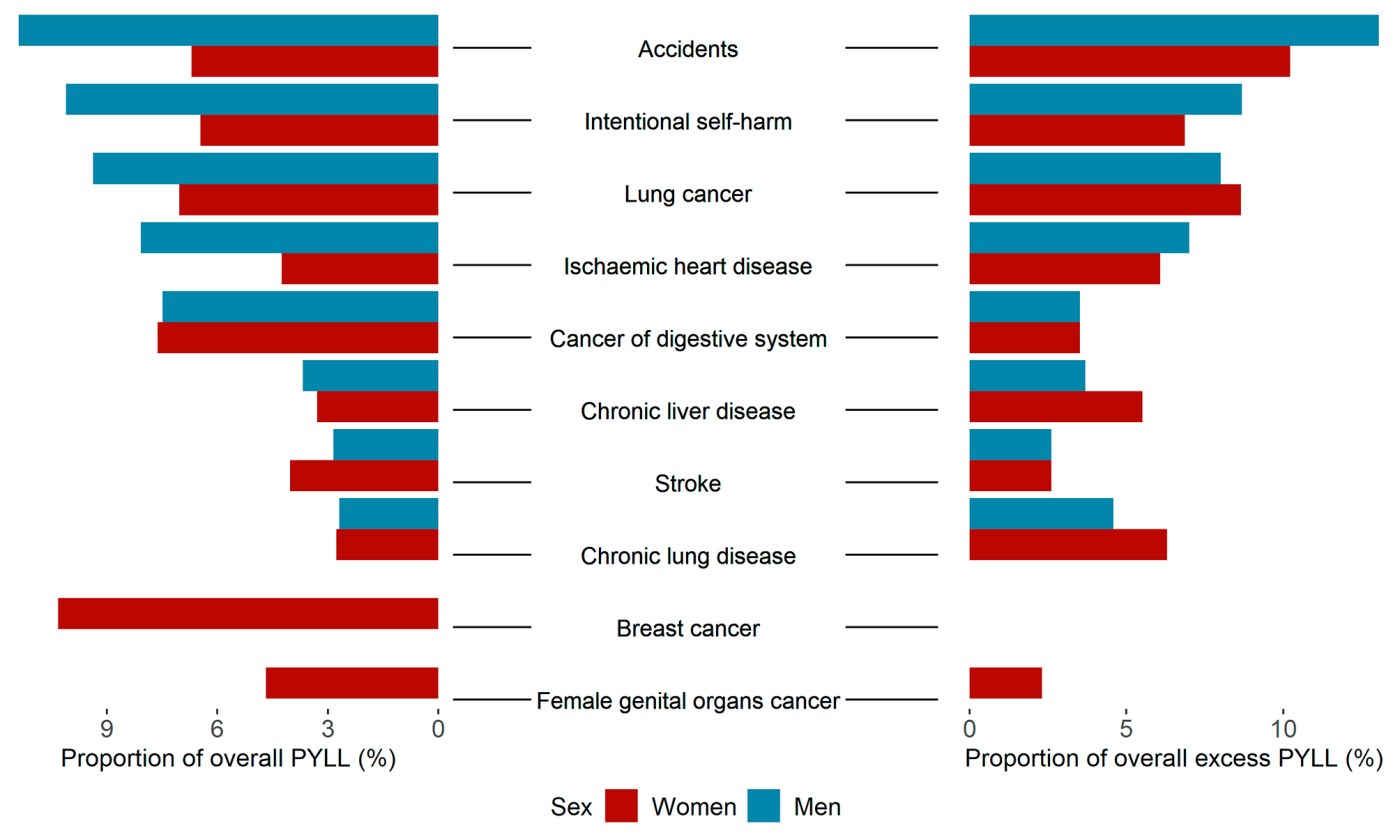
Causes of death with greatest contribution to the overall crude PYLL (left) and their contribution to the excess number of PYLL (right)

In both sexes, the greatest contributors into the overall PYLL were deaths by neoplasms, contributing by 29.4% in men and 40.9% in women. However, after decomposing the ICD-10 chapters into smaller subgroups of deaths, the results showed the following causes of deaths contributed the most to the overall PYLL (Fig. 4). In men– deaths by accident (11.4%), intentional self-harm (10.1%), lung cancer (9.4%), ischaemic heart disease (8%), cancer of digestive system (7.5%), and chronic liver disease (3.7%). In women, the following causes of death contributed the most to the overall PYLL– deaths by breast cancer (10.3%), lung cancer (7.4%), deaths by accident (6.7%), intentional self-harm (6.5%), cancer of female genital organs (4.7%), ischaemic heart disease (4.3%), stroke (4%), and chronic liver disease (3.3%).

The subgroups of cause-specific deaths contributing the most (with more than 5%) to the excess PYLL (Fig. 4) were in men the deaths by accident (305,755 − 13.5%), intentional self-harm (203,386 − 8.7%), lung cancer (187,816 − 8.01%), ischaemic heart disease (164,408 − 7.1%), chronic liver disease (129,064 − 5.5%). In women, the causes of deaths contributing with more than 5% to the overall excess PYLL included deaths by lung cancer (71,876–8.7%), intentional self-harm (74,472–6.9%), chronic lung disease (68,325–6.3%), and ischaemic heart disease (65,888–9.1%).

### Age-standardized PYLL due inequality in Belgium since 1998

We computed the age-standardized PYLL per 100,000 by sex, deciles and period. In the period studied, 6,474 PYLL and 3,418 PYLL per 100,000 men and women were observed. In men and women, PYLL were decreasing over time across all deprivation deciles. The decrease was greater among the least deprived, peaking in men (-36%). The relative difference in the most and least deprived stayed almost constant. More details are provided in Table 5.

**Table 5:** Overview of age-standardized PYLL per 100,000 persons by sex, period and the most and least deprived deciles

	All years	1998–2003	2004–2008	2009–2013	2014–2019	Absolute difference	Relative difference
Overall	5,485	6,408	5,674	5,208	4,648	-1,760	-27.47%
Men	6,474	8,261	7,283	6,398	5,592	-2,613	-30.89%
most deprived	10,192	12,076	10,792	9,564	8,337	-3,739	-30.96%
least deprived	4,817	5,944	5,070	4,449	3,804	-2,140	-36.00%
Absolute difference	-5,376	-6,131	-5,723	-5,116	-4,533	1,598	-26.06%
Relative difference	-52.7%	-50.8%	-53.0	-53.5%	-54.3%		
Women	3,418	4,242	3,914	3,631	3,336	-902	-20.78%
most deprived	5,351	5,919	5,490	5,292	4,701	-1,218	-20.58%
least deprived	2,856	3,331	2,925	2,725	2,445	-885	-26.58%
Absolute difference	-2,494	-2,589	-2,566	-2,567	-2,256	333	-12.86%
Relative difference	-46.1%	-43.7%	-46.7	-48.1%	-49.0%		

The number of observed and expected PYLL per 100,000 persons has been decreasing over time in both sexes, the relative difference in observed vs. expected has increased in men from 27.9% up to 31.6% and in women from 22.4 to 26.7%, 1998–2003 vs. 2014–2019.

A detailed overview of the number of observed and expected PYLL per 100,000 person-years is presented in Table S5 in Supplementary materials, whereas crude PYLL by causes of death stratified by men and women is shown in Tables S6 and S7 in Supplementary materials.

## Discussion

This is the first Belgian study that investigates deprivation-driven inequalities in premature mortality using the 2001 and 2011 Belgian Indices of Multiple Deprivation to shed light on pronounced socioeconomic inequalities in several health indicators. Compared to men and women living in the least deprived statistical sectors, those living in the most deprived statistical sectors had a 96% and 78% greater chance to die prematurely. Almost 28% of all premature deaths was attributable to socioeconomic inequality, corresponding to 1 person dying prematurely every hour in Belgium since 1998. About 30% of potential years lost due to socioeconomic inequality would be saved if the whole of Belgium had the premature mortality rates of the least deprived areas. The relative decrease in premature mortality was greater in men than in women (25% vs. 15%), in the most and least deprived deciles (36% vs. 25%). The inequality has increased due to faster pace of decrease in the least deprived areas compared to the most deprived areas. The relative difference between the most and least deprived areas increased in men and women by about 8%.

We observed the greatest inequality in premature mortality for respiratory, mental and behavioral, and digestive diseases, whereas much less inequality was observed for neoplasms, musculoskeletal and nervous diseases. The greatest inequality measured by the PAF and PYLL was observed in psychoactive substance use, chronic lung and liver disease, obesity, and diabetes mellitus causes, which are causes of death linked to increased opiate, alcohol and tobacco consumption, or poor diet. The PAF tended to increase with age, and peaked in men and women in the late middle-age (ages 45–64).

Our study confirms findings from previous studies that reported a decrease in premature mortality rates and an increase in health inequality in Belgium over time [13–15, 18, 23]. Findings of the current study also represent a valuable extension to our previous study (Otavova et al. 2022) in which we used area-based housing indices, later integrated into the BIMDs, to investigate association between poor housing and all-ages mortality [16]. The greater magnitude of inequality reported in the current study might suggest that housing indices only partially capture inequalities and the role of other factors, such as education and income must be considered to have a more comprehensive picture.

Although our study design does not allow us to assess a causal relationship between deprivation and health, some reasonable hypotheses can be derived to interpret the trends we observe. The substantial decrease in deaths caused by ischaemic heart disease in the most and least deprived deciles suggests a positive impact of health policies, successful education of population regarding healthy diet and healthy lifestyle [24, 25], or improvements in prevention (screening strategy) followed by an adequate medical treatment. A fall of deaths by road accidents reflects the drastic measures in road security policies [26]. Development of more effective treatments (hormone, chemo or immunotherapy) and successful implementation of screening programs led to a decrease in deaths by breast cancer [27], male and female genital cancer [28], colorectal cancer [29], lymphoid and haematological cancer by more than one third in the most and least deprived deciles.

Almost 40% and 20% increase in premature deaths caused by liver cancer in men in the most and least deprived deciles might result from a change in reporting [13], but other factors, could play a role. Thus, further studies should be conducted to explain this change.

Smoking represents the main risk factor for lung cancer, accounting for 80–90% of all cases [30]. In Belgium, lung cancer in men has been decreasing since the 1990s [13], and our results show a similar trend– a decrease across all deprivation groups and an increase in premature rates with age. Over recent decades smoking prevalence has declined faster in upper than lower socioeconomic groups in northern Europe, resulting in strong socioeconomic gradients in smoking [31, 32]. Our results also show that deaths by lung cancer decreased faster in men in the least deprived groups (-44%) compared to the most deprived groups (-31%). At the contrary, the female lung cancer premature rates increased by 42% for those living in the most deprived areas and by 32% for those living in the least deprived areas. This reflects the well-known sex differences in the timing of the smoking epidemic [33–35].

### Strengths and limitations

Our study has two major strengths: the utilization of the Belgian Indices of Multiple Deprivation and the cause-specific analysis of carefully selected health indicators. We used BIMDs that combine data on income, education, employment, housing, and crime to provide insight into health inequalities associated with upstream socioeconomic circumstances. Our decision to use these indices and measure inequality at the aggregate level was justified by several arguments. A main benefit of the BIMD use is the wider range of disadvantageous circumstances covered. The intersection of various disadvantages can concurrently influence health and health behavior, and given the diversity of the Belgian society, researchers in population health must consider the interplay between different dimensions of disadvantage– a task for which the BIMDs are well-suited. We were also interested in capturing area deprivation effects as conceptualized in three different meanings: as compositional (i.e. a proportion of deprived people living in the area), as collective, and as environmental (i.e. socio-cultural and historical features of communities or environmental features, synonymous with neighborhood effect) [36]. It has been shown that, even after accounting for individual risk factors, neighborhoods still strongly affect their inhabitants [37–41]. Using the BIMD, we were able to include all three dimensions, but it became difficult to isolate individual effects, as the tool is constructed on the aggregation of individual variables. Furthermore, an inherent strength of the indices is their easy replicability, allowing for future updates with the most recent data, and hence, a follow-up study can be conducted to track future developments in health inequalities since 2019 in Belgium.

Another major benefit of our study is a detailed cause-specific analysis of health inequalities by premature mortality, population attributable fraction, and potential years of life lost. Whereas, the PAF offers an estimate of the potential reduction in premature mortality if socioeconomic inequalities were eliminated, the PYLL completes the picture by weighting the burden of each condition with the loss of quantity of life [13]. To compute these population-level estimates of inequality, we utilized cause-specific data from the whole population of Belgium, ensuring the maximum statistical power and avoidance of selection bias. The method of computing the premature mortality associated with inequality is replicable in other settings and can be used to make comparison studies. Our results can be disintegrated by cause of death, and, due to our aggregate approach, we can spatially identify the most prevalent causes of deaths and pinpoint geographical areas in need of interventions reducing health inequalities. By ranking the causes of premature deaths based on premature mortality rates, PAF, and PYLL we are able to help setting up priorities for policy makers.

In choosing a suitable geographical area, we preferred the smallest administrative unit in Belgium, the statistical sector. This choice was validated by the fact that statistical sectors are official administrative units; their boundaries change little over time [17]; they are large enough to provide statistically robust estimates; and better homogeneity within their populations can be assumed, reducing the risk of ecological fallacy [42, 43]. Additionally, the use of aggregate data was more practical and enabled us to accommodate the privacy, security, and confidentiality issues associated with a use of the administrative and survey data more easily. We were also given an access to data that were used for the crime and health domains, that would otherwise be unavailable or the process of obtaining them would be too lengthy.

The study has limitations that need to be considered when interpreting the results. As we investigate the association between deprivation and health at the aggregate level, inferences about individuals cannot be drawn. An ecological fallacy would be committed [44] by falsely assuming that individuals living in the most deprived areas must themselves be highly deprived, and conversely, that those living in the least deprived areas must themselves be less deprived.

The indices are built on census and register data related mostly to years 2001 and 2011, but the BIMD2001 and BIMD2011 are applied to the period 1998–2008 and 2009–2019. As the Spearman’s rank correlation coefficient between the indices is 0.9, we assume that the BIMDs are fairly stable over time. However, we cannot eliminate a possible change in the level of deprivation across statistical sectors during the periods under study.

Another limitation of the current study is that we are unable to capture morbidity or quality of life aspects based on the death registration data. There is also a risk of misclassification as mortality data rely on death certificate data of which quality is questionable for certain groups and certain causes of deaths [45]. By restricting our analysis to data from 1998 to 2019 and by limiting the scope to premature deaths (1–74 years), we avoided the more questionable time periods and higher age groups in which the causes of deaths are reported with more errors and multi-morbidity plays an increasing role.

Our analysis accounted for differences in age and sex between deprivation groups but were not able to adjust for other variables, such as migrant status. The migrant status might be a confounder for diseases with a strong genetic or migration-related risk [46, 47]. Internal mobility was also not considered as the deprivation deciles could only be assigned once to the deceived, i.e. at the time of death. The place of death was identified as the place where a deceased was officially registered, but it might not correspond to the place he or she was exposed to specific risk factors across the life course.

In the current study, we measured the extent of health inequality associated with socioeconomic deprivation as defined by the BIMDs but we could not capture a causal effect of deprivation on health. The design of our cross-sectional study does not allow us to assess the causal relationship between deprivation and premature mortality and is not suitable for showing that any of the domains of the BIMDs have caused death or that their improvement would result in a decrease in premature mortality. Instead, we showed a scenario in which the whole Belgium had the same premature mortality rates as the least deprived deciles of the BIMDs, without suggesting how this scenario would be achieved. By its very nature, the study is designed to show the scale of health inequalities associated with deprivation rather than the cause of the health inequalities.

## Conclusion

We studied the trends and disparities in premature mortality in Belgium and provided a better understanding of the extent to which area-level deprivation is associated with premature deaths. Our study showed that premature mortality decreased but health inequalities increased over time and every year, thousands of deaths in Belgium could be avoided if Belgium had the premature mortality rates of the least deprived decile. The greatest inequality in causes of death are related to behavior risk factors, such as alcohol consumption or tobacco and illicit drugs use. These results emphasize the importance of a more in-depth evaluation of health and social policies, and a need of new, country-level interventions that aim to protect those with a lower socioeconomic status more effectively from health-deteriorating lifestyles and environments.

### Electronic supplementary material

Below is the link to the electronic supplementary material.

Supplementary Material 1

## Data Availability

The data that support the findings of this study are available at the Belgian statistical office, Statbel, but restrictions apply to the availability of these data, which are used under license for the current study, and so are not publicly available. Data are however available from the corresponding author upon reasonable request and with the permission of Statbel.
