# Supplementary material for: Trends in socioeconomic inequalities in cause-specific premature mortality in Belgium, 1998–2019
Source: BMC Public Health. 2024 Feb 14;24:470. doi: 10.1186/s12889-024-17933-z (PMC10868013; doi:10.1186/s12889-024-17933-z)
Supplement: Supplementary file 1 — Supplementary Material 1 [file 12889_2024_17933_MOESM1_ESM.docx]

Supplementary materials

Supplement to: Otavova M, Masquelier B, Faes C, Van Den Borre L, Vandeninden B, De Clercq E, Devleesschauwer B. Trends in socioeconomic inequalities in cause-specific premature mortality in Belgium, 1998-2019

Table of Contents

[Methods 1](#_Toc133933936)

[Age-standardized premature mortality rates 1](#_Toc133933937)

[Potential years of life lost 3](#_Toc133933938)

[Results 3](#_Toc133933939)

[Belgian indices of multiple deprivation and population characteristics across deprivation deciles 3](#_Toc133933940)

[Overall premature deaths and share of the main categories of causes of death between 1998 and 2019 and distribution across deprivation deciles 5](#_Toc133933941)

[All-cause and cause-specific age standardized premature mortality rates 9](#_Toc133933942)

[Overall and cause-specific premature mortality attributable to socioeconomic inequality in Belgium 61](#_Toc133933943)

[Potential years of life lost in Belgium since 1998 70](#_Toc133933944)

# Methods

## Age-standardized premature mortality rates

We stratified the overall and cause-specific mortality and population data to 5-year age groups (except the lowest age group made of children aged 1–4), sex, BIMDs’ deprivation deciles, and periods 1998-2003, 2004-2008, 2009-2013, and 2014-2019. We then computed sex- and age-standardized premature mortality rates per 100,000 person-years for each stratum using the sex and age structure of the European standard population 2018. The age-standardized premature rate was calculated as follows:

ASMR *=* $\sum\left( d_{i}w_{i}/y_{i} \right)$

Where *ASMR* = Age-standardized premature mortality rates; *d_i_* = age-specific deaths in the age-group *i*; *y_i_* = person-years at risk in the age group *i*; *w_i_* = weight of age group *i* in the standard EU population 2018, summing to 1.

We calculated the standard error of an age-standardized premature mortality rate using a Poisson approximation [1].

Var (ASMR) = ∑(d_i_ w_i_ ^2^*100,000/ y_i_ )/(∑ w_i_ )^2^

Where *Var*(*ASMR)* = Variance of age-standardized premature mortality rates; *d_i_* = age-specific deaths in the age-group *i*; *w_i_* = weight of age group *i* in the standard EU population 2018 summing to 1; *y_i_* = person-years at risk in the age group *i*.

The decision to use the Poisson approximation for computing the standard error of the ASMR was motivated by its alignment with the characteristics of mortality data in our study. Specifically, we conducted a detailed cause-specific analysis across several sex-, period-, and deprivation-specific strata that resulted in a small number of events in each stratum. In this case, the Poisson distribution provides a mathematically tractable and theoretically grounded approach for modeling the distribution of small numbers of deaths.

The relative difference, ASMR ratio, was computed as the ratio of two age-standardized premature mortality rates calculated by the direct method. It represents the relative risk of dying prematurely in population 1 compared to population 2.

ASMR RATIO = ASMR_1_/ASMR_10_

Where *ASMR_1_* = ASMR in the most deprived decile and *ASMR_10_* = ASMR in the least deprived decile.

The uncertainty interval for the ASMR ratio was calculated as follows:

X = (ASMR_1_ – ASMR_10_) /(√(s.e.(ASMR_1_)^2^ + s.e.(ASMR_10_)^2^)

Lower bound = (ASMR_1_/ASMR_10_)^1-(1.96/X)^

Upper bound = (ASMR_1_/ASMR_10_)^1+(1.96/X)^

## Potential years of life lost

The potential years of life lost were computed as a sum of the number of deaths at each age (between 1 and 75) multiplied by the remaining years of life up to age 7 [2].

PYLL = ∑ a_i_ d_i_

Where *a_i_* = the remaining years to live until age 75 when death occurs at age *i* (75-*i*), and *d_i_* = number of deaths at age *i*.

Besides observed or crude PYLL we computed age-standardized rate of PYLL per 100,000 person-years to compare the number of PYLL across different causes of death and deprivation deciles.

Rate of PYLL = ∑ a_i_ d_i_ x (100,000/N)

Where N = number of persons at age *i* in the actual population.

# Results

## Belgian indices of multiple deprivation and population characteristics across deprivation deciles

The 2001 and 2011 Belgian indices of multiple deprivation (without the health domains) were used to measure deprivation at the level of statistical sector. During twenty-two years included in our study, almost 211 million people aged between 1-75 years were officially registered in Belgium. Although the division of statistical sectors into deprivation deciles was balanced, proportion of population across deciles varied, whereas the sex distribution was similar across all deciles. The greatest proportion of population lived into the most deprived areas, equaling to about 9% of men and women, whereas about 4% men and women lived in the least deprived areas. The proportion of men and women living in the most deprived areas slightly increased from about 9% to 10% (1998-2003 vs 2014-2019). On the contrary, the proportion of men and women living in the least deprived areas decreased from 5 % down to 4%. The mean age was lowest in men (34.4 years) and women (35.4 years) living in the most deprived areas, whereas men and women living in the least deprived areas had a mean age of 37.4 and 38.1 years. Over time, the mean age of men and women living in the most deprived areas stayed constant, while the mean age in the least deprived areas has increased from 35.6 to 39.1 years in men and 36.2 to 39.5 years in women. Distribution of person-years from 1998 to 2019 in Belgium across the deprivation deciles is shown in Table S1 and Figure S1.

*Table S1 Overview of distribution of person-years and deaths across deprivation decile (1998-2019)*

|  | Men | | | | Women | | | |
| --- | --- | --- | --- | --- | --- | --- | --- | --- |
| Deciles | Deaths | Person-years | Deaths % | Pop  % | Deaths | Person-years | Deaths  % | Pop  % |
| 1 | 11,2066 | 19,738,832 | 14.19 | 9.36 | 63,670 | 19,386,307 | 8.06 | 9.2 |
| 2 | 67,502 | 12,167,942 | 8.55 | 5.77 | 39,854 | 12,284,671 | 5.05 | 5.83 |
| 3 | 50,611 | 9,582,380 | 6.41 | 4.55 | 29,799 | 9,729,882 | 3.77 | 4.62 |
| 4 | 43,280 | 8,784,342 | 5.48 | 4.17 | 25,488 | 8,797,812 | 3.23 | 4.17 |
| 5 | 40,667 | 8,906,917 | 5.15 | 4.23 | 23,747 | 8,845,322 | 3.01 | 4.2 |
| 6 | 38,767 | 8,580,760 | 4.91 | 4.07 | 22,606 | 8,570,098 | 2.86 | 4.07 |
| 7 | 38,553 | 9,094,327 | 4.88 | 4.31 | 22,096 | 8,980,813 | 2.8 | 4.26 |
| 8 | 35,948 | 9,023,846 | 4.55 | 4.28 | 20,694 | 8,898,004 | 2.62 | 4.22 |
| 9 | 37,042 | 9,605,786 | 4.69 | 4.56 | 20,929 | 9,378,095 | 2.65 | 4.45 |
| 10 | 35,647 | 10,290,999 | 4.51 | 4.88 | 20,700 | 1,013,005 | 2.62 | 4.81 |
| Total | 500,083 | 105,776,130 | 63.32 | 50.18 | 289,583 | 105,001,037 | 36.67 | 49.83 |


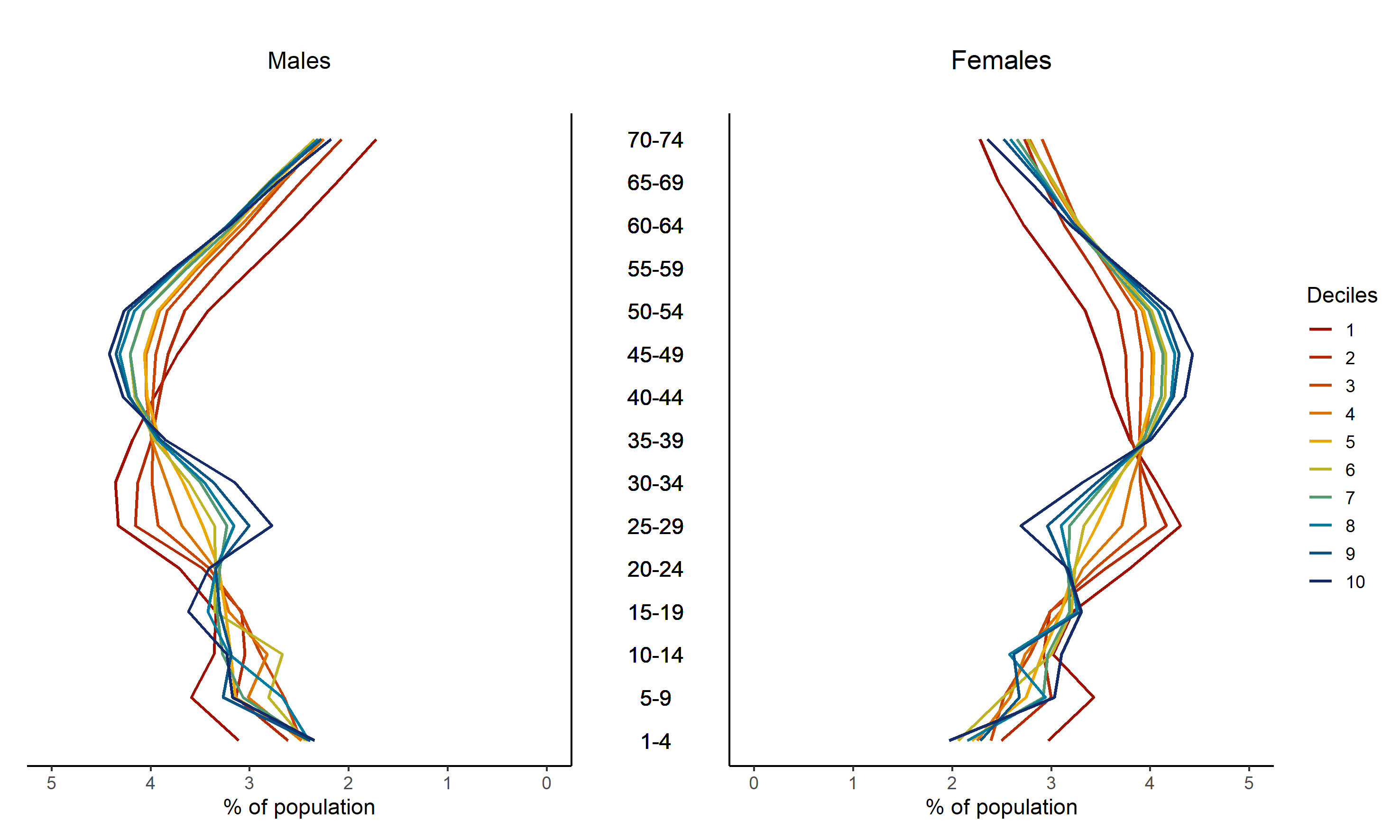


*Figure S1 Distribution of person-years from 1998 to 2019 in Belgium across the deprivation deciles*

## Overall premature deaths and share of the main categories of causes of death between 1998 and 2019 and distribution across deprivation deciles

Among the 2,315,287 deaths that occurred in Belgium in 1998-2019, we analyze here the 789,666 deaths (34.1% of the total) that occurred above age 1 and below age 75. Infant deaths represented 0.4% of all deaths, while deaths occurring in people aged 75 and over accounted for 65.9%. The number of all deaths was similar in both sexes, the proportion of premature deaths (deaths before age 75) was greater in men than in women, representing 63.3% and 36.7% of the total deaths, respectively.

Neoplasms and diseases of the circulatory system were the two leading categories of causes of premature mortality in both sexes, representing 36.1% and 22.7% in males and 42% and 19.6% in females respectively. In men, lung cancer (12%), ischaemic heart disease (10%), neoplasms of the digestive system (9%), intentional self-harm (5%), and chronic lung disease (5%) were the leading causes of death (Figure 3). In women, the most prominent causes of premature deaths were breast cancer (10%), neoplasms of the digestive system (9%), lung cancer (7%), ischaemic heart disease (6%), and cerebrovascular heart disease (5%).


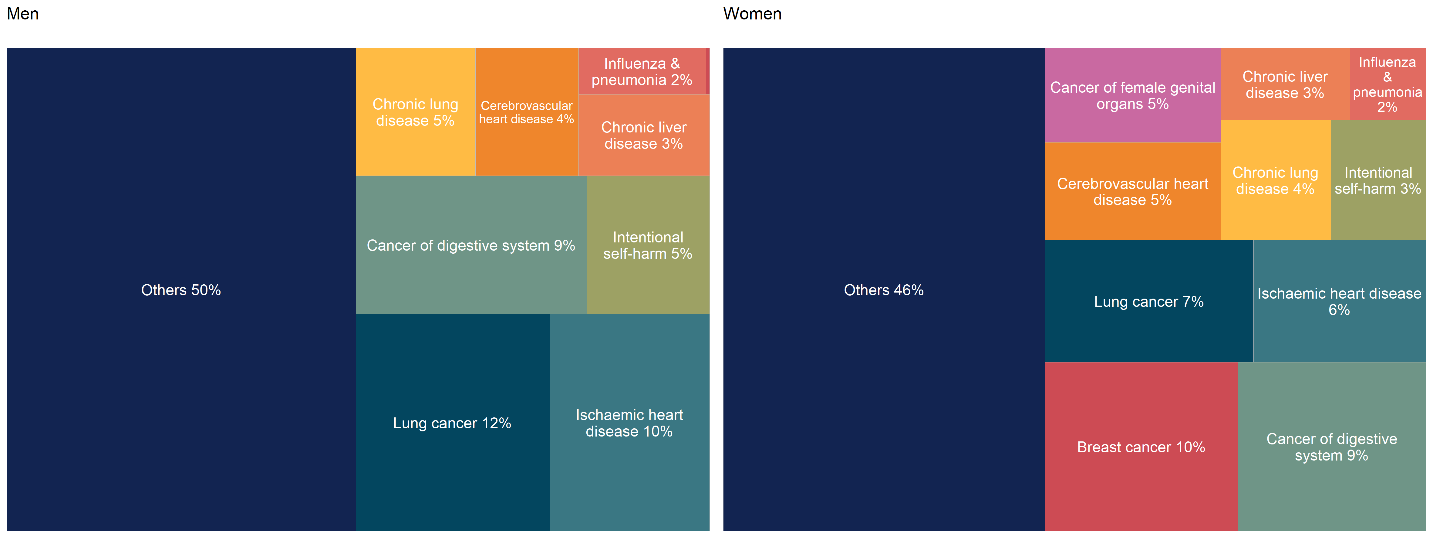


*Figure S2 Distribution of the main ICD-10 chapters in men and women*

A marked socioeconomic gradient was observed in the distribution of the total number of deaths, and premature deaths for both sexes. For men and women equally, about 18% and 7% of all-age deaths and 22% and 7% of premature deaths occurred in the most and least deprived deciles. Similar proportions of premature deaths were observed in the most and least deprived areas in all periods under study, suggesting no change over time. The distribution of cause-specific deaths in the least deprived areas was similar to those in the most deprived in men and women with small exceptions. The proportions of the largest group of causes of death, neoplasms, were greater in the least deprived areas in men (41.6%) and women (50.5%) than in the most deprived, representing an increase by 10.4% and 14.7%. In women, the proportions of causes of death related to neoplasms of female genital organs and breast were greater by 2% and 6% and in men, the proportion of lung cancer was greater by 2.2% in the least deprived areas. Figure S3 shows the distribution of the most common causes of deaths in men and women in the most and least deprived groups.


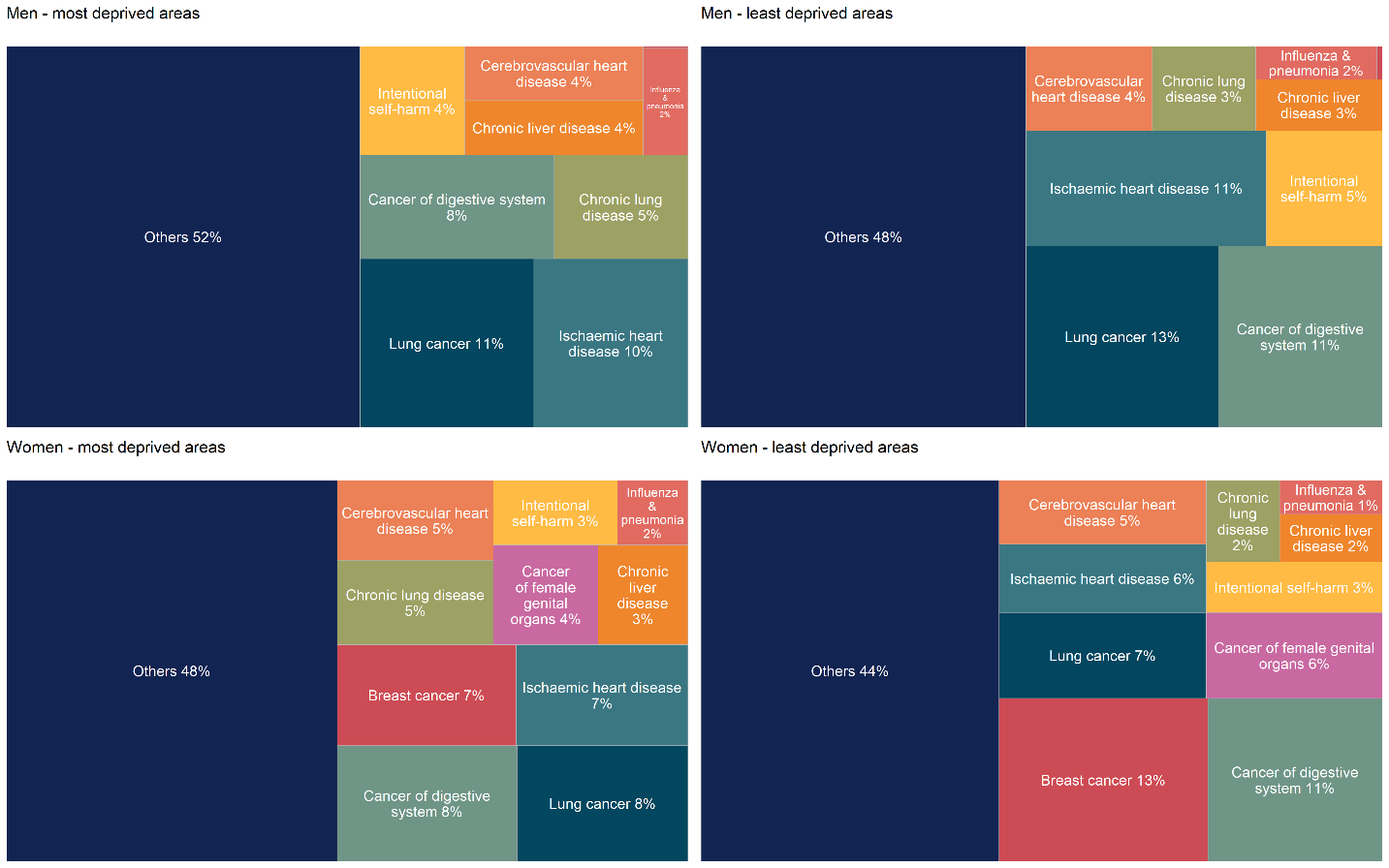


*Figure S3 Sex differences in distribution of the most common causes of deaths across the most and least deprived groups in the period between 1998 and 2019*

## All-cause and cause-specific age standardized premature mortality rates

##

Table S2 Overview of cause-specific premature mortality rates in men in Belgium from 1998 to 2019 in the most and least deprived deciles

| **Chapter** | **Subgroup** | **Diagnosis** | **ICD 10 codes** | **Period** | **Most  deprived 1st decile** | **Least  deprived 10th decile** | **Difference between  most and least  deprived** | **(%)** | **Most  deprived / least deprived** |
| --- | --- | --- | --- | --- | --- | --- | --- | --- | --- |
| **Infections** | **Viral hepatitis** |  | B15-B19 |  | 1 | 10 | Difference | (%) |  |
|  |  |  |  | 1998-2003 | 2.42 | 1.92 | -0.50 | -20.66% | 1.26 |
|  |  |  |  | 2014-2019 | 1.26 | 0.37 | -0.89 | -70.63% | 3.41 |
|  |  |  |  | Change in time | -1.16 | -1.55 |  |  |  |
|  |  |  |  | (%) | -47.93% | -80.73% |  |  |  |
|  | **Other** |  | A0-B09; B20-B99 |  | 1 | 10 | Difference | (%) |  |
|  |  |  |  | 1998-2003 | 15.63 | 7.56 | -8.07 | -51.63% | 2.07 |
|  |  |  |  | 2014-2019 | 14.43 | 4.47 | -9.96 | -69.02% | 3.23 |
|  |  |  |  | Change in time | -1.2 | -3.09 |  |  |  |
|  |  |  |  | (%) | -7.68% | -40.87% |  |  |  |
|  | **TOTAL** |  | A01-99; B00-99 |  | 1 | 10 | Difference | (%) |  |
|  |  |  |  | 1998-2003 | 17.37 | 8.14 | -9.23 | -53.14% | 2.13 |
|  |  |  |  | 2014-2019 | 15.36 | 4.55 | -10.81 | -70.38% | 3.38 |
|  |  |  |  | Change in time | -2.01 | -3.59 |  |  |  |
|  |  |  |  | (%) | -11.57% | -44.10% |  |  |  |
| **Neoplasm** | **Respiratory** | **Larynx** | C32 |  | 1 | 10 | Difference | (%) |  |
|  |  |  |  | 1998-2003 | 7 | 3.05 | -3.95 | -56.43% | 2.30 |
|  |  |  |  | 2014-2019 | 3.23 | 1.91 | -1.32 | -40.87% | 1.69 |
|  |  |  |  | Change in time | -3.77 | -1.14 |  |  |  |
|  |  |  |  | (%) | -53.86% | -37.38% |  |  |  |
|  |  | **Lung** | C34 |  | 1 | 10 | Difference | (%) |  |
|  |  |  |  | 1998-2003 | 104.41 | 68.07 | -36.34 | -34.81% | 1.53 |
|  |  |  |  | 2014-2019 | 72.06 | 38.2 | -33.86 | -46.99% | 1.89 |
|  |  |  |  | Change in time | -32.35 | -29.87 |  |  |  |
|  |  |  |  | (%) | -30.98% | -43.88% |  |  |  |
|  |  | **Other** | C30-31; C33; C35-39 |  | 1 | 10 | Difference | (%) |  |
|  |  |  |  | 1998-2003 | 3.86 | 2.39 | -1.47 | -38.08% | 1.62 |
|  |  |  |  | 2014-2019 | 1.59 | 1.93 | 0.34 | 21.38% | 0.82 |
|  |  |  |  | Change in time | -2.27 | -0.46 |  |  |  |
|  |  |  |  | (%) | -58.81% | -19.25% |  |  |  |
|  |  | **Total** | C30-39 |  | 1 | 10 | Difference | (%) |  |
|  |  |  |  | 1998-2003 | 115.02 | 72.33 | -42.69 | -37.12% | 1.59 |
|  |  |  |  | 2014-2019 | 76.16 | 40.06 | -36.10 | -47.40% | 1.90 |
|  |  |  |  | Change in time | -38.86 | -32.27 |  |  |  |
|  |  |  |  | (%) | -33.79% | -44.61% |  |  |  |
|  | **Mouth** |  | C00-14 |  | 1 | 10 | Difference | (%) |  |
|  |  |  |  | 1998-2003 | 12.92 | 5.07 | -7.85 | -60.76% | 2.55 |
|  |  |  |  | 2014-2019 | 9.49 | 4.38 | -5.11 | -53.85% | 2.17 |
|  |  |  |  | Change in time | -3.43 | -0.69 |  |  |  |
|  |  |  |  | (%) | -26.55% | -13.61% |  |  |  |
|  | **Other** |  | C76-80; C97; D00-D48 |  | 1 | 10 | Difference | (%) |  |
|  |  |  |  | 1998-2003 | 25.82 | 12.03 | -13.79 | -53.41% | 2.15 |
|  |  |  |  | 2014-2019 | 22.28 | 9.05 | -13.23 | -59.38% | 2.46 |
|  |  |  |  | Change in time | -3.54 | -2.98 |  |  |  |
|  |  |  |  | (%) | -13.71% | -24.77% |  |  |  |
|  | **Urinary** | **Bladder** | C67 |  | 1 | 10 | Difference | (%) |  |
|  |  |  |  | 1998-2003 | 9.08 | 7.59 | -1.49 | -16.41% | 1.20 |
|  |  |  |  | 2014-2019 | 6.38 | 4.16 | -2.22 | -34.80% | 1.53 |
|  |  |  |  | Change in time | -2.7 | -3.43 |  |  |  |
|  |  |  |  | (%) | -29.74% | -45.19% |  |  |  |
|  |  | **Kidney** | C64 |  | 1 | 10 | Difference | (%) |  |
|  |  |  |  | 1998-2003 | 5.1 | 5.01 | -0.09 | -1.76% | 1.02 |
|  |  |  |  | 2014-2019 | 3.72 | 4.65 | 0.93 | 25.00% | 0.80 |
|  |  |  |  | Change in time | -1.38 | -0.36 |  |  |  |
|  |  |  |  | (%) | -27.06% | -7.19% |  |  |  |
|  |  | **Other** | C65-66; C68 |  | 1 | 10 | Difference | (%) |  |
|  |  |  |  | 1998-2003 | 1.19 | 0.95 | -0.24 | -20.17% | 1.25 |
|  |  |  |  | 2014-2019 | 2.54 | 1.98 | -0.56 | -22.05% | 1.28 |
|  |  |  |  | Change in time | 1.35 | 1.03 |  |  |  |
|  |  |  |  | (%) | 113.45% | 108.42% |  |  |  |
|  |  | **Total** | C64-68 |  | 1 | 10 | Difference | (%) |  |
|  |  |  |  | 1998-2003 | 14.56 | 12.51 | -2.05 | -14.08% | 1.16 |
|  |  |  |  | 2014-2019 | 12.29 | 9.32 | -2.97 | -24.17% | 1.32 |
|  |  |  |  | Change in time | -2.27 | -3.19 |  |  |  |
|  |  |  |  | (%) | -15.59% | -25.50% |  |  |  |
|  | **Digestive** | **Colon** | C18 |  | 1 | 10 | Difference | (%) |  |
|  |  |  |  | 1998-2003 | 15.93 | 14.6 | -1.33 | -8.35% | 1.09 |
|  |  |  |  | 2014-2019 | 11.9 | 9.17 | -2.73 | -22.94% | 1.30 |
|  |  |  |  | Change in time | -4.03 | -5.43 |  |  |  |
|  |  |  |  | (%) | -25.30% | -37.19% |  |  |  |
|  |  | **Liver & IH bile ducts** | C22 |  | 1 | 10 | Difference | (%) |  |
|  |  |  |  | 1998-2003 | 8.52 | 5.07 | -3.45 | -40.49% | 1.68 |
|  |  |  |  | 2014-2019 | 12.05 | 5.91 | -6.14 | -50.95% | 2.04 |
|  |  |  |  | Change in time | 3.53 | 0.84 |  |  |  |
|  |  |  |  | (%) | 41.43% | 16.57% |  |  |  |
|  |  | **Oesophagus** | C15 |  | 1 | 10 | Difference | (%) |  |
|  |  |  |  | 1998-2003 | 9.94 | 7.13 | -2.81 | -28.27% | 1.39 |
|  |  |  |  | 2014-2019 | 8.59 | 6.46 | -2.13 | -24.80% | 1.33 |
|  |  |  |  | Change in time | -1.35 | -0.67 |  |  |  |
|  |  |  |  | (%) | -13.58% | -9.40% |  |  |  |
|  |  | **Other** | C17; C21; C23; C24; C26 |  | 1 | 10 | Difference | (%) |  |
|  |  |  |  | 1998-2003 | 4.31 | 3.59 | -0.72 | -16.71% | 1.20 |
|  |  |  |  | 2014-2019 | 3.74 | 4.3 | 0.56 | 14.97% | 0.87 |
|  |  |  |  | Change in time | -0.57 | 0.71 |  |  |  |
|  |  |  |  | (%) | -13.23% | 19.78% |  |  |  |
|  |  | **Pancreas** | C25 |  | 1 | 10 | Difference | (%) |  |
|  |  |  |  | 1998-2003 | 10.96 | 9.21 | -1.75 | -15.97% | 1.19 |
|  |  |  |  | 2014-2019 | 12.31 | 8.87 | -3.44 | -27.94% | 1.39 |
|  |  |  |  | Change in time | 1.35 | -0.34 |  |  |  |
|  |  |  |  | (%) | 12.32% | -3.69% |  |  |  |
|  |  | **Rectosigmoid junction** | C19 | 1 | 1 | 10 | Difference | (%) |  |
|  |  |  |  | 1998-2003 | 1.6 | 1.7 | 0.10 | 6.25% | 0.94 |
|  |  |  |  | 2014-2019 | 1.98 | 0.97 | -1.01 | -51.01% | 2.04 |
|  |  |  |  | Change in time | 0.38 | -0.73 |  |  |  |
|  |  |  |  | (%) | 23.75% | -42.94% |  |  |  |
|  |  | **Rectum** | C20 |  | 1 | 10 | Difference | (%) |  |
|  |  |  |  | 1998-2003 | 4.23 | 4.86 | 0.63 | 14.89% | 0.87 |
|  |  |  |  | 2014-2019 | 3.79 | 2.68 | -1.11 | -29.29% | 1.41 |
|  |  |  |  | Change in time | -0.44 | -2.18 |  |  |  |
|  |  |  |  | (%) | -10.40% | -44.86% |  |  |  |
|  |  | **Stomach** | C16 |  | 1 | 10 | Difference | (%) |  |
|  |  |  |  | 1998-2003 | 8.53 | 7.3 | -1.23 | -14.42% | 1.17 |
|  |  |  |  | 2014-2019 | 6.72 | 4.49 | -2.23 | -33.18% | 1.50 |
|  |  |  |  | Change in time | -1.81 | -2.81 |  |  |  |
|  |  |  |  | (%) | -21.22% | -38.49% |  |  |  |
|  |  | **Total** | C15-26 |  | 1 | 10 | Difference | (%) |  |
|  |  |  |  | 1998-2003 | 61.34 | 50.39 | -10.95 | -17.85% | 1.22 |
|  |  |  |  | 2014-2019 | 59.36 | 37.86 | -21.50 | -36.22% | 1.57 |
|  |  |  |  | Change in time | -1.98 | -12.53 |  |  |  |
|  |  |  |  | (%) | -3.23% | -24.87% |  |  |  |
|  | **Thyroid & endocrine glands** |  | C73-75 |  | 1 | 10 | Difference | (%) |  |
|  |  |  |  | 1998-2003 | 1.82 | 1.89 | 0.07 | 3.85% | 0.96 |
|  |  |  |  | 2014-2019 | 1.65 | 1.91 | 0.26 | 15.76% | 0.86 |
|  |  |  |  | Change in time | -0.17 | 0.02 |  |  |  |
|  |  |  |  | (%) | -9.34% | 1.06% |  |  |  |
|  | **Male genital organs** |  | C60-63 |  | 1 | 10 | Difference | (%) |  |
|  |  |  |  | 1998-2003 | 13.19 | 12.53 | -0.66 | -5.00% | 1.05 |
|  |  |  |  | 2014-2019 | 10.07 | 6.91 | -3.16 | -31.38% | 1.46 |
|  |  |  |  | Change in time | -3.12 | -5.62 |  |  |  |
|  |  |  |  | (%) | -23.65% | -44.85% |  |  |  |
|  | **Lymphoid & haematopoietic** | **Leukaemia, unspecified unspecified** | C95 |  | 1 | 10 | Difference | (%) |  |
|  |  |  |  | 1998-2003 | 2.49 | 1.77 | -0.72 | -28.92% | 1.41 |
|  |  |  |  | 2014-2019 | 1.52 | 2.08 | 0.56 | 36.84% | 0.73 |
|  |  |  |  | Change in time | -0.97 | 0.31 |  |  |  |
|  |  |  |  | (%) | -38.96% | 17.51% |  |  |  |
|  |  | **Lymphoid leukaemia** | C91 |  | 1 | 10 | Difference |  |  |
|  |  |  |  | 1998-2003 | 2.59 | 2.95 | 0.36 | 13.90% | 0.88 |
|  |  |  |  | 2014-2019 | 1.53 | 0.99 | -0.54 | -35.29% | 1.55 |
|  |  |  |  | Change in time | -1.06 | -1.96 |  |  |  |
|  |  |  |  | (%) | -40.93% | -66.44% |  |  |  |
|  |  | **Multiple myeloma** | C90 |  | 1 | 10 | Difference |  |  |
|  |  |  |  | 1998-2003 | 3.46 | 3.08 | -0.38 | -10.98% | 1.12 |
|  |  |  |  | 2014-2019 | 1.78 | 2.18 | 0.40 | 22.47% | 0.82 |
|  |  |  |  | Change in time | -1.68 | -0.9 |  |  |  |
|  |  |  |  | (%) | -48.55% | -29.22% |  |  |  |
|  |  | **Myeloid leukaemia** | C92 |  | 1 | 10 | Difference |  |  |
|  |  |  |  | 1998-2003 | 4.44 | 3.36 | -1.08 | -24.32% | 1.32 |
|  |  |  |  | 2014-2019 | 2.98 | 3.49 | 0.51 | 17.11% | 0.85 |
|  |  |  |  | Change in time | -1.46 | 0.13 |  |  |  |
|  |  |  |  | (%) | -32.88% | 3.87% |  |  |  |
|  |  | **Other** | C81-89; C93-94; C96 |  | 1 | 10 | Difference | (%) |  |
|  |  |  |  | 1998-2003 | 7.29 | 6.22 | -1.07 | -14.68% | 1.17 |
|  |  |  |  | 2014-2019 | 5.79 | 4.47 | -1.32 | -22.80% | 1.30 |
|  |  |  |  | Change in time | -1.5 | -1.75 |  |  |  |
|  |  |  |  | (%) | -20.58% | -28.14% |  |  |  |
|  |  | **Total** | C81-96 |  | 1 | 10 | Difference | (%) |  |
|  |  |  |  | 1998-2003 | 16.92 | 13.86 | -3.06 | -18.09% | 1.22 |
|  |  |  |  | 2014-2019 | 11.95 | 10.36 | -1.59 | -13.31% | 1.15 |
|  |  |  |  | Change in time | -4.97 | -3.5 |  |  |  |
|  |  |  |  | (%) | -29.37% | -25.25% |  |  |  |
|  | **Bone and articular cartilage** |  | C40-41 |  | 1 | 10 | Difference | (%) |  |
|  |  |  |  | 1998-2003 | 1.74 | 2.21 | 0.47 | 27.01% | 0.79 |
|  |  |  |  | 2014-2019 | 1.6 | 1.77 | 0.17 | 10.63% | 0.90 |
|  |  |  |  | Change in time | -0.14 | -0.44 |  |  |  |
|  |  |  |  | (%) | -8.05% | -19.91% |  |  |  |
|  | **Mesothelial and soft tissue** |  | C45-49 |  | 1 | 10 | Difference | (%) |  |
|  |  |  |  | 1998-2003 | 3.8 | 4.04 | 0.24 | 6.32% | 0.94 |
|  |  |  |  | 2014-2019 | 3.55 | 5.17 | 1.62 | 45.63% | 0.69 |
|  |  |  |  | Change in time | -0.25 | 1.13 |  |  |  |
|  |  |  |  | (%) | -6.58% | 27.97% |  |  |  |
|  | **Breast** |  | C50 |  | 1 | 10 | Difference | (%) |  |
|  |  |  |  | 1998-2003 | 1.12 | 0.97 | -0.15 | -13.39% | 1.15 |
|  |  |  |  | 2014-2019 | 0.48 | 0.64 | 0.16 | 33.33% | 0.75 |
|  |  |  |  | Change in time | -0.64 | -0.33 |  |  |  |
|  |  |  |  | (%) | -57.14% | -34.02% |  |  |  |
|  | **Eye, brain & CNS** |  | C69-72 |  | 1 | 10 | Difference | (%) |  |
|  |  |  |  | 1998-2003 | 6.77 | 7.38 | 0.61 | 9.01% | 0.92 |
|  |  |  |  | 2014-2019 | 5.52 | 8.73 | 3.21 | 58.15% | 0.63 |
|  |  |  |  | Change in time | -1.25 | 1.35 |  |  |  |
|  |  |  |  | (%) | -18.46% | 18.29% |  |  |  |
|  | **Skin** |  | C43-44 |  | 1 | 10 | Difference | (%) |  |
|  |  |  |  | 1998-2003 | 2.71 | 4.17 | 1.46 | 53.87% | 0.65 |
|  |  |  |  | 2014-2019 | 2.26 | 3.82 | 1.56 | 69.03% | 0.59 |
|  |  |  |  | Change in time | -0.45 | -0.35 |  |  |  |
|  |  |  |  | (%) | -16.61% | -8.39% |  |  |  |
|  | **TOTAL** |  | C00-97; D00-48 |  | 1 | 10 | Difference | (%) | 0.10 |
|  |  |  |  | 1998-2003 | 271.89 | 191.44 | -80.45 | -29.59% | 1.42 |
|  |  |  |  | 2014-2019 | 212.05 | 129.44 | -82.61 | -38.96% | 1.64 |
|  |  |  |  | Change in time | -59.84 | -62 |  |  |  |
|  |  |  |  | (%) | -22.01% | -32.39% |  |  |  |
| **Blood** | **TOTAL** |  | D50-89 |  | 1 | 10 | Difference | (%) |  |
|  |  |  |  | 1998-2003 | 2.93 | 1.62 | -1.31 | -44.71% | 1.81 |
|  |  |  |  | 2014-2019 | 2.72 | 1.43 | -1.29 | -47.43% | 1.90 |
|  |  |  |  | Change in time | -0.21 | -0.19 |  |  |  |
|  |  |  |  | (%) | -7.17% | -11.73% |  |  |  |
| **Endocrine, nutritional, metabolic** | **Diabetes mellitus** |  | E10-14 |  | 1 | 10 | Difference | (%) |  |
|  |  |  |  | 1998-2003 | 13.96 | 5.61 | -8.35 | -59.81% | 2.49 |
|  |  |  |  | 2014-2019 | 10.59 | 3.9 | -6.69 | -63.17% | 2.72 |
|  |  |  |  | Change in time | -3.37 | -1.71 |  |  |  |
|  |  |  |  | (%) | -24.14% | -30.48% |  |  |  |
|  | **Metabolic disorders** |  | E70-89 |  | 1 | 10 | Difference | (%) |  |
|  |  |  |  | 1998-2003 | 3.33 | 2.5 | -0.83 | -24.92% | 1.33 |
|  |  |  |  | 2014-2019 | 4.63 | 1.98 | -2.65 | -57.24% | 2.34 |
|  |  |  |  | Change in time | 1.3 | -0.52 |  |  |  |
|  |  |  |  | (%) | 39.04% | -20.80% |  |  |  |
|  | **Obesity** |  | E66-68 |  | 1 | 10 | Difference | (%) |  |
|  |  |  |  | 1998-2003 | 2.15 | 1.16 | -0.99 | -46.05% | 1.85 |
|  |  |  |  | 2014-2019 | 2.31 | 0.55 | -1.76 | -76.19% | 4.20 |
|  |  |  |  | Change in time | 0.16 | -0.61 |  |  |  |
|  |  |  |  | (%) | 7.44% | -52.59% |  |  |  |
|  | **Other** |  | E00-07; E15-63 |  | 1 | 10 | Difference | (%) |  |
|  |  |  |  | 1998-2003 | 1.59 | 0.7 | -0.89 | -55.97% | 2.27 |
|  |  |  |  | 2014-2019 | 1.64 | 0.77 | -0.87 | -53.05% | 2.13 |
|  |  |  |  | Change in time | 0.05 | 0.07 |  |  |  |
|  |  |  |  | (%) | 3.14% | 10.00% |  |  |  |
|  | **TOTAL** |  | E00-E89 |  | 1 | 10 | Difference | (%) |  |
|  |  |  |  | 1998-2003 | 19.24 | 7.45 | -11.79 | -61.28% | 2.58 |
|  |  |  |  | 2014-2019 | 17.7 | 5.34 | -12.36 | -69.83% | 3.31 |
|  |  |  |  | Change in time | -1.54 | -2.11 |  |  |  |
|  |  |  |  | (%) | -8.00% | -28.32% |  |  |  |
| **Mental behavioural** | **Psychoactive substance use** | **Alcohol** | F10 |  | 1 | 10 | Difference | (%) |  |
|  |  |  |  | 1998-2003 | 9.99 | 2.18 | -7.81 | -78.18% | 4.58 |
|  |  |  |  | 2014-2019 | 9.88 | 3.02 | -6.86 | -69.43% | 3.27 |
|  |  |  |  | Change in time | -0.11 | 0.84 |  |  |  |
|  |  |  |  | (%) | -1.10% | 38.53% |  |  |  |
|  |  | **Other** | F11-19 |  | 1 | 10 | Difference | (%) |  |
|  |  |  |  | 1998-2003 | 4.37 | 1.23 | -3.14 | -71.85% | 3.55 |
|  |  |  |  | 2014-2019 | 2.92 | 0.26 | -2.66 | -91.10% | 11.23 |
|  |  |  |  | Change in time | -1.45 | -0.97 |  |  |  |
|  |  |  |  | (%) | -33.18% | -78.86% |  |  |  |
|  | **Psychoactive substance use** | **Total** | F10-19 |  | 1 | 10 | Difference | (%) |  |
|  |  |  |  | 1998-2003 | 13.75 | 2.75 | -11.00 | -80.00% | 5.00 |
|  |  |  |  | 2014-2019 | 12.3 | 3.03 | -9.27 | -75.37% | 4.06 |
|  |  |  |  | Change in time | -1.45 | 0.28 |  |  |  |
|  |  |  |  | (%) | -10.55% | 10.18% |  |  |  |
|  | **Organic** |  | F01-F09 |  | 1 | 10 | Difference | (%) |  |
|  |  |  |  | 1998-2003 | 3.73 | 2.63 | -1.10 | -29.49% | 1.42 |
|  |  |  |  | 2014-2019 | 3.35 | 2.74 | -0.61 | -18.21% | 1.22 |
|  |  |  |  | Change in time | -0.38 | 0.11 |  |  |  |
|  |  |  |  | (%) | -10.19% | 4.18% |  |  |  |
|  | **Other** |  | F20-99 |  | 1 | 10 | Difference | (%) |  |
|  |  |  |  | 1998-2003 | 1.96 | 0.99 | -0.97 | -49.49% | 1.98 |
|  |  |  |  | 2014-2019 | 2.22 | 1.6 | -0.62 | -27.93% | 1.39 |
|  |  |  |  | Change in time | 0.26 | 0.61 |  |  |  |
|  |  |  |  | (%) | 13.27% | 61.62% |  |  |  |
|  | **TOTAL** |  | F01-99 |  | 1 | 10 | Difference | (%) |  |
|  |  |  |  | 1998-2003 | 18.39 | 4.99 | -13.40 | -72.87% | 3.69 |
|  |  |  |  | 2014-2019 | 16.98 | 5.32 | -11.66 | -68.67% | 3.19 |
|  |  |  |  | Change in time | -1.41 | 0.33 |  |  |  |
|  |  |  |  | (%) | -7.67% | 6.61% |  |  |  |
| **Nervous** | **Episodic & paroxysmal** |  | G40-47 |  | 1 | 10 | Difference | (%) |  |
|  |  |  |  | 1998-2003 | 3.91 | 2.74 | -1.17 | -29.92% | 1.43 |
|  |  |  |  | 2014-2019 | 3.78 | 2.34 | -1.44 | -38.10% | 1.62 |
|  |  |  |  | Change in time | -0.13 | -0.4 |  |  |  |
|  |  |  |  | (%) | -3.32% | -14.60% |  |  |  |
|  | **Other** |  | G00-09; G50-64; G70-72; G80--83; G90-98 |  | 1 | 10 | Difference | (%) |  |
|  |  |  |  | 1998-2003 | 4.96 | 2.93 | -2.03 | -40.93% | 1.69 |
|  |  |  |  | 2014-2019 | 5.37 | 4.71 | -0.66 | -12.29% | 1.14 |
|  |  |  |  | Change in time | 0.41 | 1.78 |  |  |  |
|  |  |  |  | (%) | 8.27% | 60.75% |  |  |  |
|  | **Other degenerative** |  | G30-31 |  | 1 | 10 | Difference | (%) |  |
|  |  |  |  | 1998-2003 | 3.79 | 2.84 | -0.95 | -25.07% | 1.33 |
|  |  |  |  | 2014-2019 | 3.78 | 3.6 | -0.18 | -4.76% | 1.05 |
|  |  |  |  | Change in time | -0.01 | 0.76 |  |  |  |
|  |  |  |  | (%) | -0.26% | 26.76% |  |  |  |
|  | **Demyelinating diseases of CNS** |  | G35-37 |  | 1 | 10 | Difference | (%) |  |
|  |  |  |  | 1998-2003 | 2.11 | 1.42 | -0.69 | -32.70% | 1.49 |
|  |  |  |  | 2014-2019 | 1.32 | 2.18 | 0.86 | 65.15% | 0.61 |
|  |  |  |  | Change in time | -0.79 | 0.76 |  |  |  |
|  |  |  |  | (%) | -37.44% | 53.52% |  |  |  |
|  | **Extrapyramidal & movement** |  | G20-26 |  | 1 | 10 | Difference | (%) |  |
|  |  |  |  | 1998-2003 | 2.98 | 2.12 | -0.86 | -28.86% | 1.41 |
|  |  |  |  | 2014-2019 | 3.02 | 3.66 | 0.64 | 21.19% | 0.83 |
|  |  |  |  | Change in time | 0.04 | 1.54 |  |  |  |
|  |  |  |  | (%) | 1.34% | 72.64% |  |  |  |
|  | **Systemic atrophies of CNS** |  | G10-14 |  | 1 | 10 | Difference | (%) |  |
|  |  |  |  | 1998-2003 | 2.63 | 3.27 | 0.64 | 24.33% | 0.80 |
|  |  |  |  | 2014-2019 | 2.63 | 4.01 | 1.38 | 52.47% | 0.66 |
|  |  |  |  | Change in time | 0 | 0.74 |  |  |  |
|  |  |  |  | (%) | 0.00% | 22.63% |  |  |  |
|  | **TOTAL** |  | G00-98 |  | 1 | 10 | Difference | (%) |  |
|  |  |  |  | 1998-2003 | 17.51 | 10.6 | -6.91 | -39.46% | 1.65 |
|  |  |  |  | 2014-2019 | 17.77 | 15.94 | -1.83 | -10.30% | 1.11 |
|  |  |  |  | Change in time | 0.26 | 5.34 |  |  |  |
|  |  |  |  | (%) | 1.48% | 50.38% |  |  |  |
| **Circulatory** | **Hypertensive** |  | I10-15 |  | 1 | 10 | Difference | (%) |  |
|  |  |  |  | 1998-2003 | 3.89 | 1.57 | -2.32 | -59.64% |  |
|  |  |  |  | 2014-2019 | 2.68 | 1.64 | -1.04 | -38.81% |  |
|  |  |  |  | Change in time | -1.21 | 0.07 |  |  |  |
|  |  |  |  | (%) | -31.11% | 4.46% |  |  |  |
|  | **Ischaemic heart disease** | **Acute MI** | I21 |  | 1 | 10 | Difference | (%) |  |
|  |  |  |  | 1998-2003 | 69.31 | 47.43 | -21.88 | -31.57% | 1.46 |
|  |  |  |  | 2014-2019 | 35.3 | 14.83 | -20.47 | -57.99% | 2.38 |
|  |  |  |  | Change in time | -34.01 | -32.6 |  |  |  |
|  |  |  |  | (%) | -49.07% | -68.73% |  |  |  |
|  |  | **Chronic** | I25 |  | 1 | 10 | Difference | (%) |  |
|  |  |  |  | 1998-2003 | 33.62 | 18.41 | -15.21 | -45.24% | 1.83 |
|  |  |  |  | 2014-2019 | 19.09 | 7.42 | -11.67 | -61.13% | 2.57 |
|  |  |  |  | Change in time | -14.53 | -10.99 |  |  |  |
|  |  |  |  | (%) | -43.22% | -59.70% |  |  |  |
|  |  | **Other** | I20; I22-24 |  | 1 | 10 | Difference | (%) |  |
|  |  |  |  | 1998-2003 | 5.87 | 3.23 | -2.64 | -44.97% | 1.82 |
|  |  |  |  | 2014-2019 | 2.51 | 1.96 | -0.55 | -21.91% | 1.28 |
|  |  |  |  | Change in time | -3.36 | -1.27 |  |  |  |
|  |  |  |  | (%) | -57.24% | -39.32% |  |  |  |
|  |  | **Total** | I20-25 |  | 1 | 10 | Difference | (%) |  |
|  |  |  |  | 1998-2003 | 108.35 | 68.05 | -40.30 | -37.19% | 1.59 |
|  |  |  |  | 2014-2019 | 56.6 | 22.86 | -33.74 | -59.61% | 2.48 |
|  |  |  |  | Change in time | -51.75 | -45.19 |  |  |  |
|  |  |  |  | (%) | -47.76% | -66.41% |  |  |  |
|  | **Pulmonary** | **Other** | I27-28 |  | 1 | 10 | Difference | (%) |  |
|  |  |  |  | 1998-2003 | 1.16 | 1.04 | -0.12 | -10.34% | 1.12 |
|  |  |  |  | 2014-2019 | 1.41 | 1 | -0.41 | -29.08% | 1.41 |
|  |  |  |  | Change in time | 0.25 | -0.04 |  |  |  |
|  |  |  |  | (%) | 21.55% | -3.85% |  |  |  |
|  |  | **Pulmonary embolism** | I26 |  | 1 | 10 | Difference | (%) |  |
|  |  |  |  | 1998-2003 | 5.59 | 2.61 | -2.98 | -53.31% | 2.14 |
|  |  |  |  | 2014-2019 | 4.81 | 2.68 | -2.13 | -44.28% | 1.79 |
|  |  |  |  | Change in time | -0.78 | 0.07 |  |  |  |
|  |  |  |  | (%) | -13.95% | 2.68% |  |  |  |
|  |  | **Total** | I26-28 |  | 1 | 10 | Difference | (%) |  |
|  |  |  |  | 1998-2003 | 5.71 | 2.7 | -3.01 | -52.71% | 2.11 |
|  |  |  |  | 2014-2019 | 5.12 | 2.87 | -2.25 | -43.95% | 1.78 |
|  |  |  |  | Change in time | -0.59 | 0.17 |  |  |  |
|  |  |  |  | (%) | -10.33% | 6.30% |  |  |  |
|  | **Veins, lymph, vessels and nodes** |  | I80-89 |  | 1 | 10 | Difference | (%) |  |
|  |  |  |  | 1998-2003 | 2.31 | 1.2 | -1.11 | -48.05% | 1.93 |
|  |  |  |  | 2014-2019 | 2.11 | 0.73 | -1.38 | -65.40% | 2.89 |
|  |  |  |  | Change in time | -0.2 | -0.47 |  |  |  |
|  |  |  |  | (%) | -8.66% | -39.17% |  |  |  |
|  | **Other types of heart disease** | **Atrial fibrillation & flutter** | I48 |  | 1 | 10 | Difference | (%) |  |
|  |  |  |  | 1998-2003 | 2.54 | 2.27 | -0.27 | -10.63% | 1.12 |
|  |  |  |  | 2014-2019 | 2.41 | 1.69 | -0.72 | -29.88% | 1.43 |
|  |  |  |  | Change in time | -0.13 | -0.58 |  |  |  |
|  |  |  |  | (%) | -5.12% | -25.55% |  |  |  |
|  |  | **Cardiomyopathy** | I42 |  | 1 | 10 | Difference | (%) |  |
|  |  |  |  | 1998-2003 | 3.99 | 3.63 | -0.36 | -9.02% | 1.10 |
|  |  |  |  | 2014-2019 | 3.17 | 2.94 | -0.23 | -7.26% | 1.08 |
|  |  |  |  | Change in time | -0.82 | -0.69 |  |  |  |
|  |  |  |  | (%) | -20.66% | -18.79% |  |  |  |
|  |  | **Heart failure** | I50 |  | 1 | 10 | Difference | (%) |  |
|  |  |  |  | 1998-2003 | 11.21 | 6.87 | -4.34 | -38.72% | 1.63 |
|  |  |  |  | 2014-2019 | 9.5 | 6.1 | -3.40 | -35.79% | 1.56 |
|  |  |  |  | Change in time | -1.71 | -0.77 |  |  |  |
|  |  |  |  | (%) | -15.25% | -11.21% |  |  |  |
|  |  | **Other** | I30-40; I43-47; I49; I51 |  | 1 | 10 | Difference | (%) |  |
|  |  |  |  | 1998-2003 | 29.54 | 16.71 | -12.83 | -43.43% | 1.77 |
|  |  |  |  | 2014-2019 | 19.21 | 9.6 | -9.61 | -50.03% | 2.00 |
|  |  |  |  | Change in time | -10.33 | -7.11 |  |  |  |
|  |  |  |  | (%) | -34.97% | -42.55% |  |  |  |
|  |  | **Total** | I30-51 |  | 1 | 10 | Difference | (%) |  |
|  |  |  |  | 1998-2003 | 48.8 | 28.88 | -19.92 | -40.82% | 1.69 |
|  |  |  |  | 2014-2019 | 35.34 | 18.11 | -17.23 | -48.75% | 1.95 |
|  |  |  |  | Change in time | -13.46 | -10.77 |  |  |  |
|  |  |  |  | (%) | -27.58% | -37.29% |  |  |  |
|  | **Stroke** | **Haemorrhage** | I60-62 |  | 1 | 10 | Difference | (%) |  |
|  |  |  |  | 1998-2003 | 14.19 | 10.17 | -4.02 | -28.33% | 1.40 |
|  |  |  |  | 2014-2019 | 9.68 | 5.09 | -4.59 | -47.42% | 1.90 |
|  |  |  |  | Change in time | -4.51 | -5.08 |  |  |  |
|  |  |  |  | (%) | -31.76% | -50.00% |  |  |  |
|  |  | **Infarction** | I63 |  | 1 | 10 | Difference | (%) |  |
|  |  |  |  | 1998-2003 | 4.36 | 3.95 | -0.41 | -9.40% | 1.10 |
|  |  |  |  | 2014-2019 | 3.84 | 2.78 | -1.06 | -27.60% | 1.38 |
|  |  |  |  | Change in time | -0.52 | -1.17 |  |  |  |
|  |  |  |  | (%) | -11.93% | -29.62% |  |  |  |
|  |  | **Other** | I64-69 |  | 1 | 10 | Difference | (%) |  |
|  |  |  |  | 1998-2003 | 18.24 | 10.37 | -7.87 | -43.15% | 1.76 |
|  |  |  |  | 2014-2019 | 10.99 | 4.64 | -6.35 | -57.78% | 2.37 |
|  |  |  |  | Change in time | -7.25 | -5.73 |  |  |  |
|  |  |  |  | (%) | -39.75% | -55.26% |  |  |  |
|  |  | **Total** | I60-69 |  | 1 | 10 | Difference | (%) |  |
|  |  |  |  | 1998-2003 | 32.83 | 21.35 | -11.48 | -34.97% | 1.54 |
|  |  |  |  | 2014-2019 | 21.82 | 10.32 | -11.5 | -52.70% | 2.11 |
|  |  |  |  | Change in time | -11.01 | -11.03 |  |  |  |
|  |  |  |  | (%) | -33.54% | -51.66% |  |  |  |
|  | **Arteries** | **Aortic aneurysm & dis.** | I71 |  | 1 | 10 | Change | (%) |  |
|  |  |  |  | 1998-2003 | 6.78 | 6.2 | -0.58 | -8.55% | 1.09 |
|  |  |  |  | 2014-2019 | 3.97 | 2.28 | -1.69 | -42.57% | 1.74 |
|  |  |  |  | Change in time | -2.81 | -3.92 |  |  |  |
|  |  |  |  | (%) | -41.45% | -63.23% |  |  |  |
|  |  | **Other** | I70; I72-79 |  | 1 | 10 | Difference | (%) |  |
|  |  |  |  | 1998-2003 | 7.4 | 3.09 | -4.31 | -58.24% | 2.39 |
|  |  |  |  | 2014-2019 | 4.91 | 2.11 | -2.80 | -57.03% | 2.33 |
|  |  |  |  | Change in time | -2.49 | -0.98 |  |  |  |
|  |  |  |  | (%) | -33.65% | -31.72% |  |  |  |
|  |  | **Total** | I70-79 |  | 1 | 10 | Difference | (%) |  |
|  |  |  |  | 1998-2003 | 13.88 | 8.86 | -5.02 | -36.17% | 1.57 |
|  |  |  |  | 2014-2019 | 8.47 | 3.88 | -4.59 | -54.19% | 2.18 |
|  |  |  |  | Change in time | -5.41 | -4.98 |  |  |  |
|  |  |  |  | (%) | -38.98% | -56.21% |  |  |  |
|  | **Other** |  | I00-09; I95-99 |  | 1 | 10 | Difference | (%) |  |
|  |  |  |  | 1998-2003 | 1.67 | 1.22 | -0.45 | -26.95% | 1.37 |
|  |  |  |  | 2014-2019 | 1.61 | 1.13 | -0.48 | -29.81% | 1.42 |
|  |  |  |  | Change in time | -0.06 | -0.09 |  |  |  |
|  |  |  |  | (%) | -3.59% | -7.38% |  |  |  |
|  | **TOTAL** |  | I00-99 |  | 1 | 10 | Difference | (%) |  |
|  |  |  |  | 1998-2003 | 220.42 | 133.72 | -86.70 | -39.33% | 1.65 |
|  |  |  |  | 2014-2019 | 134.79 | 57.87 | -76.92 | -57.07% | 2.33 |
|  |  |  |  | Change in time | -85.63 | -75.85 |  |  |  |
|  |  |  |  | (%) | -38.85% | -56.72% |  |  |  |
| **Respiratory** | **Chronic lung disease** | **Asthma** | J45 |  | 1 | 10 | Difference | (%) |  |
|  |  |  |  | 1998-2003 | 2.96 | 1.16 | -1.80 | -60.81% | 2.55 |
|  |  |  |  | 2014-2019 | 1.23 | 0.16 | -1.07 | -86.99% | 7.69 |
|  |  |  |  | Change in time | -1.73 | -1 |  |  |  |
|  |  |  |  | (%) | -58.45% | -86.21% |  |  |  |
|  |  | **Other (COPD)** | J40-44; J46-47 |  | 1 | 10 | Difference | (%) |  |
|  |  |  |  | 1998-2003 | 55.04 | 19.59 | -35.45 | -64.41% | 2.81 |
|  |  |  |  | 2014-2019 | 33.38 | 9.94 | -23.44 | -70.22% | 3.36 |
|  |  |  |  | Change in time | -21.66 | -9.65 |  |  |  |
|  |  |  |  | (%) | -39.35% | -49.26% |  |  |  |
|  |  | **Total** | J40-47 |  | 1 | 10 | Difference | (%) |  |
|  |  |  |  | 1998-2003 | 57.2 | 20.27 | -36.93 | -64.56% | 2.82 |
|  |  |  |  | 2014-2019 | 34.07 | 9.97 | -24.10 | -70.74% | 3.42 |
|  |  |  |  | Change in time | -23.13 | -10.3 |  |  |  |
|  |  |  |  | (%) | -40.44% | -50.81% |  |  |  |
|  | **Influenza & pneumonia** |  | J09-J18 |  | 1 | 10 | Difference | (%) |  |
|  |  |  |  | 1998-2003 | 17.06 | 9.49 | -7.57 | -44.37% | 1.80 |
|  |  |  |  | 2014-2019 | 13.93 | 5.26 | -8.67 | -62.24% | 2.65 |
|  |  |  |  | Change in time | -3.13 | -4.23 |  |  |  |
|  |  |  |  | (%) | -18.35% | -44.57% |  |  |  |
|  | **External agents** |  | J60-70 |  | 1 | 10 | Difference | (%) |  |
|  |  |  |  | 1998-2003 | 9.98 | 2.95 | -7.03 | -70.44% | 3.38 |
|  |  |  |  | 2014-2019 | 3.03 | 1.81 | -1.22 | -40.26% | 1.67 |
|  |  |  |  | Change in time | -6.95 | -1.14 |  |  |  |
|  |  |  |  | (%) | -69.64% | -38.64% |  |  |  |
|  | **Other** |  | J00-08; J20-22; J30-39; J80-99 |  | 1 | 10 | Difference | (%) |  |
|  |  |  |  | 1998-2003 | 11.83 | 5.42 | -6.41 | -54.18% | 2.18 |
|  |  |  |  | 2014-2019 | 9.48 | 4.98 | -4.50 | -47.47% | 1.90 |
|  |  |  |  | Change in time | -2.35 | -0.44 |  |  |  |
|  |  |  |  | (%) | -19.86% | -8.12% |  |  |  |
|  | **TOTAL** |  | J00-99 |  | 1 | 10 | Difference | (%) |  |
|  |  |  |  | 1998-2003 | 95.08 | 36.18 | -58.90 | -61.95% | 2.63 |
|  |  |  |  | 2014-2019 | 59.61 | 20.35 | -39.26 | -65.86% | 2.93 |
|  |  |  |  | Change in time | -35.47 | -15.83 |  |  |  |
|  |  |  |  | (%) | -37.31% | -43.75% |  |  |  |
| **Digestive** | **Liver** | **Alcoholic liver disease** | K70 |  | 1 | 10 | Difference | (%) |  |
|  |  |  |  | 1998-2003 | 19.28 | 5.71 | -13.57 | -70.38% | 3.38 |
|  |  |  |  | 2014-2019 | 13.17 | 4.42 | -8.75 | -66.44% | 2.98 |
|  |  |  |  | Change in time | -6.11 | -1.29 |  |  |  |
|  |  |  |  | (%) | -31.69% | -22.59% |  |  |  |
|  |  | **Fibrosis and cirrhosis** | K74 |  | 1 | 10 | Difference | (%) |  |
|  |  |  |  | 1998-2003 | 8.22 | 3.74 | -4.48 | -54.50% | 2.20 |
|  |  |  |  | 2014-2019 | 9.99 | 3.86 | -6.13 | -61.36% | 2.59 |
|  |  |  |  | Change in time | 1.77 | 0.12 |  |  |  |
|  |  |  |  | (%) | 21.53% | 3.21% |  |  |  |
|  |  | **Other** | K71-73; K75-76 |  | 1 | 10 | Difference | (%) |  |
|  |  |  |  | 1998-2003 | 2.42 | 1.58 | -0.84 | -34.71% | 1.53 |
|  |  |  |  | 2014-2019 | 2.57 | 1.99 | -0.58 | -22.57% | 1.29 |
|  |  |  |  | Change in time | 0.15 | 0.41 |  |  |  |
|  |  |  |  | (%) | 6.20% | 25.95% |  |  |  |
|  |  | **Total** | K70-77 |  | 1 | 10 | Difference | (%) |  |
|  |  |  |  | 1998-2003 | 29.23 | 9.98 | -19.25 | -65.86% | 2.93 |
|  |  |  |  | 2014-2019 | 25.34 | 9.29 | -16.05 | -63.34% | 2.73 |
|  |  |  |  | Change in time | -3.89 | -0.69 |  |  |  |
|  |  |  |  | (%) | -13.31% | -6.91% |  |  |  |
|  | **Oesophagus, stomach, duodenum** |  | K20-31 |  | 1 | 10 | Difference | (%) |  |
|  |  |  |  | 1998-2003 | 3.93 | 1.45 | -2.48 | -63.10% | 2.71 |
|  |  |  |  | 2014-2019 | 2.16 | 1.57 | -0.59 | -27.31% | 1.38 |
|  |  |  |  | Change in time | -1.77 | 0.12 |  |  |  |
|  |  |  |  | (%) | -45.04% | 8.28% |  |  |  |
|  | **Gallbladder, biliary tract, pancreas** |  | K80-87 |  | 1 | 10 | Difference | (%) |  |
|  |  |  |  | 1998-2003 | 3.85 | 2.08 | -1.77 | -45.97% | 1.85 |
|  |  |  |  | 2014-2019 | 3.32 | 1.74 | -1.58 | -47.59% | 1.91 |
|  |  |  |  | Change in time | -0.53 | -0.34 |  |  |  |
|  |  |  |  | (%) | -13.77% | -16.35% |  |  |  |
|  | **Other** |  | K00-14; K35-38, K40-46; K65-66; K90-93 |  | 1 | 10 | Difference | (%) |  |
|  |  |  |  | 1998-2003 | 5.62 | 2.59 | -3.03 | -53.91% | 2.17 |
|  |  |  |  | 2014-2019 | 5.5 | 2.33 | -3.17 | -57.64% | 2.36 |
|  |  |  |  | Change in time | -0.12 | -0.26 |  |  |  |
|  |  |  |  | (%) | -2.14% | -10.04% |  |  |  |
|  | **Other intestines** |  | K50-64 |  | 1 | 10 | Difference | (%) |  |
|  |  |  |  | 1998-2003 | 6.59 | 5.24 | -1.35 | -20.49% | 1.26 |
|  |  |  |  | 2014-2019 | 7.03 | 3.25 | -3.78 | -53.77% | 2.16 |
|  |  |  |  | Change in time | 0.44 | -1.99 |  |  |  |
|  |  |  |  | (%) | 6.68% | -37.98% |  |  |  |
|  | **TOTAL** |  | K00-93 |  | 1 | 10 | Difference | (%) |  |
|  |  |  |  | 1998-2003 | 48.11 | 17.94 | -30.17 | -62.71% | 2.68 |
|  |  |  |  | 2014-2019 | 42.49 | 15.18 | -27.31 | -64.27% | 2.80 |
|  |  |  |  | Change in time | -5.62 | -2.76 |  |  |  |
|  |  |  |  | (%) | -11.68% | -15.38% |  |  |  |
| **Skin and subcutaneous tissue** | **TOTAL** |  | L00-L99 |  | 1 | 10 | Difference | (%) |  |
|  |  |  |  | 1998-2003 | 1.08 | 0.68 | -0.40 | -37.04% | 1.59 |
|  |  |  |  | 2014-2019 | 1.26 | 0.66 | -0.60 | -47.62% | 1.91 |
|  |  |  |  | Change in time | 0.18 | -0.02 |  |  |  |
|  |  |  |  | (%) | 16.67% | -2.94% |  |  |  |
| **Musculoskeletal** | **TOTAL** |  | M00-99 |  | 1 | 10 | Difference | (%) |  |
|  |  |  |  | 1998-2003 | 3.01 | 1.59 | -1.42 | -47.18% | 1.89 |
|  |  |  |  | 2014-2019 | 2.72 | 1.56 | -1.16 | -42.65% | 1.74 |
|  |  |  |  | Change in time | -0.29 | -0.03 |  |  |  |
|  |  |  |  | (%) | -9.63% | -1.89% |  |  |  |
| **Genitourinary system** | **Other** |  | N00-16; N20-99 |  | 1 | 10 | Difference | (%) |  |
|  |  |  |  | 1998-2003 | 2.73 | 1.42 | -1.31 | -47.99% | 1.92 |
|  |  |  |  | 2014-2019 | 4 | 1.51 | -2.49 | -62.25% | 2.65 |
|  |  |  |  | Change in time | 1.27 | 0.09 |  |  |  |
|  |  |  |  | (%) | 46.52% | 6.34% |  |  |  |
|  | **Renal failure** |  | N17-19 |  | 1 | 10 | Difference | (%) |  |
|  |  |  |  | 1998-2003 | 5.73 | 2.28 | -3.45 | -60.21% | 2.51 |
|  |  |  |  | 2014-2019 | 3.52 | 1.47 | -2.05 | -58.24% | 2.39 |
|  |  |  |  | Change in time | -2.21 | -0.81 |  |  |  |
|  |  |  |  | (%) | -38.57% | -35.53% |  |  |  |
|  | **TOTAL** |  | N00-99 |  | 1 | 10 | Difference | (%) |  |
|  |  |  |  | 1998-2003 | 7.98 | 2.95 | -5.03 | -63.03% | 2.71 |
|  |  |  |  | 2014-2019 | 7.1 | 2.73 | -4.37 | -61.55% | 2.60 |
|  |  |  |  | Change in time | -0.88 | -0.22 |  |  |  |
|  |  |  |  | (%) | -11.03% | -7.46% |  |  |  |
| **Symptoms, signs,  abnormal findings** | **TOTAL** |  | R00-99 |  | 1 | 10 | Difference | (%) |  |
|  |  |  |  | 1998-2003 | 58.5 | 16.92 | -41.58 | -71.08% | 3.46 |
|  |  |  |  | 2014-2019 | 52.18 | 14.82 | -37.36 | -71.60% | 3.52 |
|  |  |  |  | Change in time | -6.32 | -2.1 |  |  |  |
|  |  |  |  | (%) | -10.80% | -12.41% |  |  |  |
| **External** | **Undetermined intent** |  | Y10-34 |  | 1 | 10 | Difference | (%) |  |
|  |  |  |  | 1998-2003 | 4.6 | 1.82 | -2.78 | -60.43% | 2.53 |
|  |  |  |  | 2014-2019 | 7.78 | 5.15 | -2.63 | -33.80% | 1.51 |
|  |  |  |  | Change in time | 3.18 | 3.33 |  |  |  |
|  |  |  |  | (%) | 69.13% | 182.97% |  |  |  |
|  | **Accidents** | **Drugs, alcohol poisoning** | X41-42; X44-45 |  | 1 | 10 | Difference | (%) |  |
|  |  |  |  | 1998-2003 | 2.46 | 0.69 | -1.77 | -71.95% | 3.57 |
|  |  |  |  | 2014-2019 | 4.85 | 2.03 | -2.82 | -58.14% | 2.39 |
|  |  |  |  | Change in time | 2.39 | 1.34 |  |  |  |
|  |  |  |  | (%) | 97.15% | 194.20% |  |  |  |
|  |  | **Other** | X00-40; X43; X46-59; W00-98; V00; V07-08; V37; V45; V57; V68; V80; V83; V86; V90-97 |  | 1 | 10 | Difference | (%) |  |
|  |  |  |  | 1998-2003 | 18.85 | 6.86 | -11.99 | -63.61% | 2.75 |
|  |  |  |  | 2014-2019 | 21.21 | 8.13 | -13.08 | -61.67% | 2.61 |
|  |  |  |  | Change in time | 2.36 | 1.27 |  |  |  |
|  |  |  |  | (%) | 12.52% | 18.51% |  |  |  |
|  |  | **Vehicle accidents** | V01-06; V09-34; V38-44; V46-55;  V58-67; V69-79; V81-82; V84-85; V87-89 |  | 1 | 10 | Difference | (%) |  |
|  |  |  |  | 1998-2003 | 16.93 | 11.1 | -5.83 | -34.44% | 1.53 |
|  |  |  |  | 2014-2019 | 7.61 | 6.43 | -1.18 | -15.51% | 1.18 |
|  |  |  |  | Change in time | -9.32 | -4.67 |  |  |  |
|  |  |  |  | (%) | -55.05% | -42.07% |  |  |  |
|  |  | **Total** | X00-59; V00-97; W00-98 |  | 1 | 10 | Difference | (%) |  |
|  |  |  |  | 1998-2003 | 36.75 | 17.27 | -19.48 | -53.01% | 2.13 |
|  |  |  |  | 2014-2019 | 33.32 | 13.79 | -19.53 | -58.61% | 2.42 |
|  |  |  |  | Change in time | -3.43 | -3.48 |  |  |  |
|  |  |  |  | (%) | -9.33% | -20.15% |  |  |  |
|  | **Other** |  | X85-99; Y00-09; Y35-36; Y85-98 |  | 1 | 10 | Difference | (%) |  |
|  |  |  |  | 1998-2003 | 5.21 | 2.69 | -2.52 | -48.37% | 1.94 |
|  |  |  |  | 2014-2019 | 4.77 | 2.91 | -1.86 | -38.99% | 1.64 |
|  |  |  |  | Change in time | -0.44 | 0.22 |  |  |  |
|  |  |  |  | (%) | -8.45% | 8.18% |  |  |  |
|  | **Intentional self-harm** |  | X60-84 |  | 1 | 10 | Difference | (%) |  |
|  |  |  |  | 1998-2003 | 29.61 | 14.5 | -15.11 | -51.03% | 2.04 |
|  |  |  |  | 2014-2019 | 21.78 | 18.56 | -3.22 | -14.78% | 1.17 |
|  |  |  |  | Change in time | -7.83 | 4.06 |  |  |  |
|  |  |  |  | (%) | -26.44% | 28.00% |  |  |  |
|  | **TOTAL** |  | X00-99; V00-97; W00-98; Y00-98 |  | 1 | 10 | Difference | (%) |  |
|  |  |  |  | 1998-2003 | 74.99 | 33.37 | -41.62 | -55.50% | 2.25 |
|  |  |  |  | 2014-2019 | 66.86 | 37.28 | -29.58 | -44.24% | 1.79 |
|  |  |  |  | Change in time | -8.13 | 3.91 |  |  |  |
|  |  |  |  | (%) | -10.84% | 11.72% |  |  |  |
| **Congenital** | **TOTAL** |  | Q00-99 |  | 1 | 10 | Difference | (%) |  |
|  |  |  |  | 1998-2003 | 2.18 | 3.04 | 0.86 | 39.45% | 0.72 |
|  |  |  |  | 2014-2019 | 1.91 | 2.84 | 0.93 | 48.69% | 0.67 |
|  |  |  |  | Change in time | -0.27 | -0.2 |  |  |  |
|  |  |  |  | (%) | -12.39% | -6.58% |  |  |  |
| **Other** | **TOTAL** |  | P00-96; O00-99; H05-82; Z05-ZZ; S18; T32-98; U00-49; U83-85 |  | 1 | 10 | Difference | (%) |  |
|  |  |  |  | 1998-2003 | 12.55 | 15.17 | 2.62 | 20.88% | 0.83 |
|  |  |  |  | 2014-2019 | 0.22 | 0 | -0.22 | -100.00% | 0.00 |
|  |  |  |  | Change in time | -12.33 | -15.17 |  |  |  |
|  |  |  |  | (%) | -98.25% | -100.00% |  |  |  |
| **ALL-CAUSE** | **TOTAL** |  |  |  | 1 | 10 | Difference | (%) |  |
|  |  |  |  | 1998-2003 | 865.72 | 474.83 | -390.89 | -45.15% | 1.82 |
|  |  |  |  | 2014-2019 | 647.8 | 301.79 | -346.01 | -53.41% | 2.15 |
|  |  |  |  | Change in time | -217.92 | -173.04 |  |  |  |
|  |  |  |  | (%) | -25.17% | -36.44% |  |  |  |

Table S3 Overview of cause-specific premature mortality rates in women in Belgium from 1998 to 2019 in the most and least deprived deciles

| **Chapter** | **Subgroup** | **Diagnosis** | **ICD 10 codes** | **Period** | **Most  deprived 1st decile** | **Least  deprived 10th decile** | **Difference between  most and least  deprived** | **(%)** | **Most  deprived / least deprived** |
| --- | --- | --- | --- | --- | --- | --- | --- | --- | --- |
| **Infections** | **Viral hepatitis** |  | B15-B19 |  | 1 | 10 | Difference | (%) |  |
|  |  |  |  | 1998-2003 | 1.91 | 0.82 | -1.09 | -57.07% | 2.32 |
|  |  |  |  | 2014-2019 | 1.32 | 0.4 | -0.92 | -69.70% | 3.33 |
|  |  |  |  | Change in time | -0.59 | -0.42 |  |  |  |
|  |  |  |  | (%) | -30.89% | -51.22% |  |  |  |
|  | **Other** |  | A0-B09; B20-B99 |  | 1 | 10 | Difference | (%) |  |
|  |  |  |  | 1998-2003 | 8.59 | 5.57 | -3.02 | -35.16% | 1.54 |
|  |  |  |  | 2014-2019 | 8.12 | 3.19 | -4.93 | -60.71% | 2.55 |
|  |  |  |  | Change in time | -0.47 | -2.38 |  |  |  |
|  |  |  |  | (%) | -5.47% | -42.73% |  |  |  |
|  |  |  | A01-99; B00-99 |  | 1 | 10 | Difference | (%) |  |
|  | **TOTAL** |  |  | 1998-2003 | 10.07 | 5.96 | -4.11 | -40.81% | 1.69 |
|  |  |  |  | 2014-2019 | 8.82 | 3.27 | -5.55 | -62.93% | 2.69 |
|  |  |  |  | Change in time | -1.25 | -2.69 |  |  |  |
|  |  |  |  | (%) | -12.41% | -45.13% |  |  |  |
| **Neoplasm** | **Respiratory** | **Larynx** | C32 |  | 1 | 10 | Difference | (%) |  |
|  |  |  |  | 1998-2003 | 1.57 | 0.55 | -1.02 | -64.97% | 2.86 |
|  |  |  |  | 2014-2019 | 0.93 | 0.7 | -0.23 | -24.73% | 1.31 |
|  |  |  |  | Change in time | -0.64 | 0.15 |  |  |  |
|  |  |  |  | (%) | -40.76% | 27.27% |  |  |  |
|  |  | **Lung** | C34 |  | 1 | 10 | Difference | (%) |  |
|  |  |  |  | 1998-2003 | 22.79 | 12.36 | -10.43 | -45.77% | 1.84 |
|  |  |  |  | 2014-2019 | 32.35 | 16.32 | -16.03 | -49.55% | 1.98 |
|  |  |  |  | Change in time | 9.56 | 3.96 |  |  |  |
|  |  |  |  | (%) | 41.95% | 32.04% |  |  |  |
|  |  | **Other** | C30-31; C33; C35-39 |  | 1 | 10 | Difference | (%) |  |
|  |  |  |  | 1998-2003 | 1.46 | 1.57 | 0.11 | 7.53% | 0.93 |
|  |  |  |  | 2014-2019 | 0.7 | 0.81 | 0.11 | 15.71% | 0.86 |
|  |  |  |  | Change in time | -0.76 | -0.76 |  |  |  |
|  |  |  |  | (%) | -52.05% | -48.41% |  |  |  |
|  |  | **Total** | C30-39 |  | 1 | 10 | Difference | (%) |  |
|  |  |  |  | 1998-2003 | 24.62 | 13.07 | -11.55 | -46.91% | 1.88 |
|  |  |  |  | 2014-2019 | 33.35 | 16.76 | -16.59 | -49.75% | 1.99 |
|  |  |  |  | Change in time | 8.73 | 3.69 |  |  |  |
|  |  |  |  | (%) | 35.46% | 28.23% |  |  |  |
|  | **Mouth** |  | C00-14 |  | 1 | 10 | Difference | (%) |  |
|  |  |  |  | 1998-2003 | 2.81 | 1.39 | -1.42 | -50.53% | 2.03 |
|  |  |  |  | 2014-2019 | 3.11 | 2.11 | -1.00 | -32.15% | 1.47 |
|  |  |  |  | Change in time | 0.30 | 0.72 |  |  |  |
|  |  |  |  | (%) | 10.68% | 51.80% |  |  |  |
|  | **Other** |  | C76-80; C97; D00-D48 |  | 1 | 10 | Difference | (%) |  |
|  |  |  |  | 1998-2003 | 12.84 | 7.58 | -5.26 | -40.97% | 1.69 |
|  |  |  |  | 2014-2019 | 11.09 | 6.39 | -4.70 | -42.38% | 1.74 |
|  |  |  |  | Change in time | -1.75 | -1.19 |  |  |  |
|  |  |  |  | (%) | -13.63% | -15.70% |  |  |  |
|  | **Urinary** | **Bladder** | C67 |  | 1 | 10 | Difference | (%) |  |
|  |  |  |  | 1998-2003 | 2.4 | 1.83 | -0.57 | -23.75% | 1.31 |
|  |  |  |  | 2014-2019 | 1.8 | 1.3 | -0.50 | -27.78% | 1.39 |
|  |  |  |  | Change in time | -0.60 | -0.53 |  |  |  |
|  |  |  |  | (%) | -25.00% | -28.96% |  |  |  |
|  |  | **Kidney** | C64 |  | 1 | 10 | Difference | (%) |  |
|  |  |  |  | 1998-2003 | 2.42 | 3.28 | 0.86 | 35.54% | 0.74 |
|  |  |  |  | 2014-2019 | 1.83 | 1.57 | -0.26 | -14.21% | 1.16 |
|  |  |  |  | Change in time | -0.59 | -1.71 |  |  |  |
|  |  |  |  | (%) | -24.38% | -52.13% |  |  |  |
|  |  | **Other** | C65-66; C68 |  | 1 | 10 | Difference | (%) |  |
|  |  |  |  | 1998-2003 | 0.45 | 1.1 | 0.65 | 144.44% | 0.41 |
|  |  |  |  | 2014-2019 | 1.14 | 1.21 | 0.07 | 6.14% | 0.94 |
|  |  |  |  | Change in time | 0.69 | 0.11 |  |  |  |
|  |  |  |  | (%) | 153.33% | 10.00% |  |  |  |
|  |  | **Total** | C64-68 |  | 1 | 10 | Difference | (%) |  |
|  |  |  |  | 1998-2003 | 4.63 | 4.65 | 0.02 | 0.43% | 0.99 |
|  |  |  |  | 2014-2019 | 3.91 | 2.85 | -1.06 | -27.11% | 1.37 |
|  |  |  |  | Change in time | -0.72 | -1.80 |  |  |  |
|  |  |  |  | (%) | -15.55% | -38.71% |  |  |  |
|  | **Digestive** | **Colon** | C18 |  | 1 | 10 | Difference | (%) |  |
|  |  |  |  | 1998-2003 | 9.7 | 9.84 | 0.14 | 1.44% | 0.99 |
|  |  |  |  | 2014-2019 | 7.29 | 6.13 | -1.16 | -15.91% | 1.19 |
|  |  |  |  | Change in time | -2.41 | -3.71 |  |  |  |
|  |  |  |  | (%) | -24.85% | -37.70% |  |  |  |
|  |  | **Liver & IH bile ducts** | C22 |  | 1 | 10 | Difference | (%) |  |
|  |  |  |  | 1998-2003 | 3.87 | 2.49 | -1.38 | -35.66% | 1.56 |
|  |  |  |  | 2014-2019 | 3.91 | 3.04 | -0.87 | -22.25% | 1.29 |
|  |  |  |  | Change in time | 0.04 | 0.55 |  |  |  |
|  |  |  |  | (%) | 1.03% | 22.09% |  |  |  |
|  |  | **Oesophagus** | C15 |  | 1 | 10 | Difference | (%) |  |
|  |  |  |  | 1998-2003 | 2.81 | 1.77 | -1.04 | -37.01% | 1.59 |
|  |  |  |  | 2014-2019 | 2.95 | 2.1 | -0.85 | -28.81% | 1.41 |
|  |  |  |  | Change in time | 0.14 | 0.33 |  |  |  |
|  |  |  |  | (%) | 4.98% | 18.64% |  |  |  |
|  |  | **Other** | C17; C21; C23; C24; C26 |  | 1 | 10 | Difference | (%) |  |
|  |  |  |  | 1998-2003 | 3.18 | 3.97 | 0.79 | 24.84% | 0.80 |
|  |  |  |  | 2014-2019 | 2.69 | 2.92 | 0.23 | 8.55% | 0.92 |
|  |  |  |  | Change in time | -0.49 | -1.05 |  |  |  |
|  |  |  |  | (%) | -15.41% | -26.45% |  |  |  |
|  |  | **Pancreas** | C25 |  | 1 | 10 | Difference | (%) |  |
|  |  |  |  | 1998-2003 | 7.5 | 6.36 | -1.14 | -15.20% | 1.18 |
|  |  |  |  | 2014-2019 | 9.6 | 6.78 | -2.82 | -29.38% | 1.42 |
|  |  |  |  | Change in time | 2.10 | 0.42 |  |  |  |
|  |  |  |  | (%) | 28.00% | 6.60% |  |  |  |
|  |  | **Rectosigmoid junction** | C19 |  | 1 | 10 | Difference | (%) |  |
|  |  |  |  | 1998-2003 | 0.92 | 0.95 | 0.03 | 3.26% | 0.97 |
|  |  |  |  | 2014-2019 | 1.31 | 1.32 | 0.01 | 0.76% | 0.99 |
|  |  |  |  | Change in time | 0.39 | 0.37 |  |  |  |
|  |  |  |  | (%) | 42.39% | 38.95% |  |  |  |
|  |  | **Rectum** | C20 |  | 1 | 10 | Difference | (%) |  |
|  |  |  |  | 1998-2003 | 2.5 | 2.68 | 0.18 | 7.20% | 0.93 |
|  |  |  |  | 2014-2019 | 2.59 | 1.84 | -0.75 | -28.96% | 1.40 |
|  |  |  |  | Change in time | 0.09 | -0.84 |  |  |  |
|  |  |  |  | (%) | 3.60% | -31.34% |  |  |  |
|  |  | **Stomach** | C16 |  | 1 | 10 | Difference | (%) |  |
|  |  |  |  | 1998-2003 | 3.97 | 2.72 | -1.25 | -31.49% | 1.46 |
|  |  |  |  | 2014-2019 | 2.92 | 2.72 | -0.20 | -6.85% | 1.07 |
|  |  |  |  | Change in time | -1.05 | 0.00 |  |  |  |
|  |  |  |  | (%) | -26.45% | 0.00% |  |  |  |
|  |  | **Total** | C15-26 |  | 1 | 10 | Difference | (%) |  |
|  |  |  |  | 1998-2003 | 32.4 | 26.75 | -5.65 | -17.44% | 1.21 |
|  |  |  |  | 2014-2019 | 30.77 | 21.3 | -9.47 | -30.78% | 1.44 |
|  |  |  |  | Change in time | -1.63 | -5.45 |  |  |  |
|  |  |  |  | (%) | -5.03% | -20.37% |  |  |  |
|  | **Female genital organs** |  | C51-58 |  | 1 | 10 | Difference | (%) |  |
|  |  |  |  | 1998-2003 | 17.05 | 14.69 | -2.36 | -13.84% | 1.16 |
|  |  |  |  | 2014-2019 | 13.91 | 9.91 | -4.00 | -28.76% | 1.40 |
|  |  |  |  | Change in time | -3.14 | -4.78 |  |  |  |
|  |  |  |  | (%) | -18.42% | -32.54% |  |  |  |
|  | **Thyroid & endocrine glands** |  | C73-75 |  | 1 | 10 | Difference | (%) |  |
|  |  |  |  | 1998-2003 | 1.53 | 1.47 | -0.06 | -3.92% | 1.04 |
|  |  |  |  | 2014-2019 | 1.48 | 2.13 | 0.65 | 43.92% | 0.70 |
|  |  |  |  | Change in time | -0.05 | 0.66 |  |  |  |
|  |  |  |  | (%) | -3.27% | 44.90% |  |  |  |
|  | **Lymphoid & haematopoietic** | **Leukaemia, unspecified unspecified** | C95 |  | 1 | 10 | Difference | (%) |  |
|  |  |  |  | 1998-2003 | 1.51 | 1.81 | 0.30 | 19.87% | 0.84 |
|  |  |  |  | 2014-2019 | 1.29 | 0.82 | -0.47 | -36.43% | 1.57 |
|  |  |  |  | Change in time | -0.22 | -0.99 |  |  |  |
|  |  |  |  | (%) | -14.57% | -54.70% |  |  |  |
|  |  | **Lymphoid leukaemia** | C91 |  | 1 | 10 | Difference | (%) |  |
|  |  |  |  | 1998-2003 | 1.42 | 1.9 | 0.48 | 33.80% | 0.75 |
|  |  |  |  | 2014-2019 | 1 | 1.69 | 0.69 | 69.00% | 0.59 |
|  |  |  |  | Change in time | -0.42 | -0.21 |  |  |  |
|  |  |  |  | (%) | -29.58% | -11.05% |  |  |  |
|  |  | **Multiple myeloma** | C90 |  | 1 | 10 | Difference | (%) |  |
|  |  |  |  | 1998-2003 | 2.57 | 2.55 | -0.02 | -0.78% | 1.00 |
|  |  |  |  | 2014-2019 | 1.57 | 2.33 | 0.76 | 48.41% | 0.67 |
|  |  |  |  | Change in time | -1.00 | -0.22 |  |  |  |
|  |  |  |  | (%) | -38.91% | -8.63% |  |  |  |
|  |  | **Myeloid leukaemia** | C92 | 1 | 1 | 10 | Difference | (%) |  |
|  |  |  |  | 1998-2003 | 3.23 | 2.61 | -0.62 | -19.20% | 1.24 |
|  |  |  |  | 2014-2019 | 2.73 | 2.55 | -0.18 | -6.59% | 1.07 |
|  |  |  |  | Change in time | -0.50 | -0.06 |  |  |  |
|  |  |  |  | (%) | -15.48% | -2.30% |  |  |  |
|  |  | **Other** | C81-89; C93-94; C96 |  | 1 | 10 | Difference | (%) |  |
|  |  |  |  | 1998-2003 | 4.4 | 4.81 | 0.41 | 9.32% | 0.91 |
|  |  |  |  | 2014-2019 | 2.74 | 2.2 | -0.54 | -19.71% | 1.25 |
|  |  |  |  | Change in time | -1.66 | -2.61 |  |  |  |
|  |  |  |  | (%) | -37.73% | -54.26% |  |  |  |
|  |  | **Total** | C81-96 |  | 1 | 10 | Difference | (%) |  |
|  |  |  |  | 1998-2003 | 10.42 | 9.89 | -0.53 | -5.09% | 1.05 |
|  |  |  |  | 2014-2019 | 7.63 | 6.75 | -0.88 | -11.53% | 1.13 |
|  |  |  |  | Change in time | -2.79 | -3.14 |  |  |  |
|  |  |  |  | (%) | -26.78% | -31.75% |  |  |  |
|  | **Bone and articular cartilage** |  | C40-41 |  | 1 | 10 | Difference | (%) |  |
|  |  |  |  | 1998-2003 | 1.24 | 1.3 | 0.06 | 4.84% | 0.95 |
|  |  |  |  | 2014-2019 | 0.96 | 1.38 | 0.42 | 43.75% | 0.70 |
|  |  |  |  | Change in time | -0.28 | 0.08 |  |  |  |
|  |  |  |  | (%) | -22.58% | 6.15% |  |  |  |
|  | **Mesothelial and soft tissue** |  | C45-49 |  | 1 | 10 | Difference | (%) |  |
|  |  |  |  | 1998-2003 | 2.19 | 2.8 | 0.61 | 27.85% | 0.78 |
|  |  |  |  | 2014-2019 | 1.93 | 3.05 | 1.12 | 58.03% | 0.64 |
|  |  |  |  | Change in time | -0.26 | 0.25 |  |  |  |
|  |  |  |  | (%) | -11.87% | 8.93% |  |  |  |
|  | **Breast** |  | C50 |  | 1 | 10 | Difference | (%) |  |
|  |  |  |  | 1998-2003 | 29.64 | 32.45 | 2.81 | 9.48% | 0.91 |
|  |  |  |  | 2014-2019 | 22.65 | 21.66 | -0.99 | -4.37% | 1.05 |
|  |  |  |  | Change in time | -6.99 | -10.79 |  |  |  |
|  |  |  |  | (%) | -23.58% | -33.25% |  |  |  |
|  | **Eye, brain & CNS** |  | C69-72 |  | 1 | 10 | Difference | (%) |  |
|  |  |  |  | 1998-2003 | 4.82 | 5.81 | 0.99 | 20.54% | 0.83 |
|  |  |  |  | 2014-2019 | 4.32 | 5.09 | 0.77 | 17.82% | 0.85 |
|  |  |  |  | Change in time | -0.50 | -0.72 |  |  |  |
|  |  |  |  | (%) | -10.37% | -12.39% |  |  |  |
|  | **Skin** |  | C43-44 |  | 1 | 10 | Difference | (%) |  |
|  |  |  |  | 1998-2003 | 2.21 | 3.24 | 1.03 | 46.61% | 0.68 |
|  |  |  |  | 2014-2019 | 2.08 | 3.17 | 1.09 | 52.40% | 0.66 |
|  |  |  |  | Change in time | -0.13 | -0.07 |  |  |  |
|  |  |  |  | (%) | -5.88% | -2.16% |  |  |  |
|  | **TOTAL** |  | C00-97; D00-48 |  | 1 | 10 | Difference | (%) |  |
|  |  |  |  | 1998-2003 | 140.45 | 117.62 | -22.83 | -16.25% | 1.19 |
|  |  |  |  | 2014-2019 | 132.36 | 93.76 | -38.60 | -29.16% | 1.41 |
|  |  |  |  | Change in time | -8.09 | -23.86 |  |  |  |
|  |  |  |  | (%) | -5.76% | -20.29% |  |  |  |
| **Blood** | **TOTAL** |  | D50-89 |  | 1 | 10 | Difference | (%) |  |
|  |  |  |  | 1998-2003 | 2.27 | 1.54 | -0.73 | -32.16% | 1.48 |
|  |  |  |  | 2014-2019 | 1.8 | 1.7 | -0.10 | -5.56% | 1.06 |
|  |  |  |  | Change in time | -0.47 | 0.16 |  |  |  |
|  |  |  |  | (%) | -20.70% | 10.39% |  |  |  |
| **Endocrine, nutritional, metabolic** | **Diabetes mellitus** |  | E10-14 |  | 1 | 10 | Difference | (%) |  |
|  |  |  |  | 1998-2003 | 8.55 | 4.31 | -4.24 | -49.59% | 1.98 |
|  |  |  |  | 2014-2019 | 4.58 | 1.39 | -3.19 | -69.65% | 3.31 |
|  |  |  |  | Change in time | -3.97 | -2.92 |  |  |  |
|  |  |  |  | (%) | -46.43% | -67.75% |  |  |  |
|  | **Metabolic disorders** |  | E70-89 |  | 1 | 10 | Difference | (%) |  |
|  |  |  |  | 1998-2003 | 2.49 | 1.92 | -0.57 | -22.89% | 1.30 |
|  |  |  |  | 2014-2019 | 2.74 | 2.37 | -0.37 | -13.50% | 1.15 |
|  |  |  |  | Change in time | 0.25 | 0.45 |  |  |  |
|  |  |  |  | (%) | 10.04% | 23.44% |  |  |  |
|  | **Obesity** |  | E66-68 |  | 1 | 10 | Difference | (%) |  |
|  |  |  |  | 1998-2003 | 1.96 | 0.71 | -1.25 | -63.78% | 2.75 |
|  |  |  |  | 2014-2019 | 2.07 | 1.04 | -1.03 | -49.76% | 2.00 |
|  |  |  |  | Change in time | 0.11 | 0.33 |  |  |  |
|  |  |  |  | (%) | 5.61% | 46.48% |  |  |  |
|  | **Other** |  | E00-07; E15-63 |  | 1 | 10 | Difference | (%) |  |
|  |  |  |  | 1998-2003 | 1.6 | 1.03 | -0.57 | -35.63% | 1.57 |
|  |  |  |  | 2014-2019 | 1.28 | 1.19 | -0.09 | -7.03% | 1.07 |
|  |  |  |  | Change in time | -0.32 | 0.16 |  |  |  |
|  |  |  |  | (%) | -20.00% | 15.53% |  |  |  |
|  | **TOTAL** |  | E00-E89 |  | 1 | 10 | Difference | (%) |  |
|  |  |  |  | 1998-2003 | 12.69 | 5.78 | -6.91 | -54.45% | 2.20 |
|  |  |  |  | 2014-2019 | 9.35 | 3.97 | -5.38 | -57.54% | 2.35 |
|  |  |  |  | Change in time | -3.34 | -1.81 |  |  |  |
|  |  |  |  | (%) | -26.32% | -31.31% |  |  |  |
| **Mental behavioural** | **Psychoactive substance use** | **Alcohol** | F10 |  | 1 | 10 | Difference | (%) |  |
|  |  |  |  | 1998-2003 | 3.07 | 1.61 | -1.46 | -47.56% | 1.92 |
|  |  |  |  | 2014-2019 | 3.3 | 1.37 | -1.93 | -58.48% | 2.41 |
|  |  |  |  | Change in time | 0.23 | -0.24 |  |  |  |
|  |  |  |  | (%) | 7.49% | -14.91% |  |  |  |
|  |  | **Other** | F11-19 |  | 1 | 10 | Difference | (%) |  |
|  |  |  |  | 1998-2003 | 1.39 | 1.07 | -0.32 | -23.02% | 1.31 |
|  |  |  |  | 2014-2019 | 1.21 | 0.41 | -0.80 | -66.12% | 2.92 |
|  |  |  |  | Change in time | -0.18 | -0.66 |  |  |  |
|  |  |  |  | (%) | -12.95% | -61.68% |  |  |  |
|  |  | **Total** | F10-19 |  | 1 | 10 | Difference | (%) |  |
|  |  |  |  | 1998-2003 | 3.78 | 1.74 | -2.04 | -53.97% | 2.18 |
|  |  |  |  | 2014-2019 | 3.97 | 1.66 | -2.31 | -58.19% | 2.39 |
|  |  |  |  | Change in time | 0.19 | -0.08 |  |  |  |
|  |  |  |  | (%) | 5.03% | -4.60% |  |  |  |
|  | **Organic** |  | F01-F09 |  | 1 | 10 | Difference | (%) |  |
|  |  |  |  | 1998-2003 | 2.41 | 2.51 | 0.10 | 4.15% | 0.96 |
|  |  |  |  | 2014-2019 | 2.38 | 2.37 | -0.01 | -0.42% | 1.00 |
|  |  |  |  | Change in time | -0.03 | -0.14 |  |  |  |
|  |  |  |  | (%) | -1.24% | -5.58% |  |  |  |
|  | **Other** |  | F20-99 |  | 1 | 10 | Difference | (%) |  |
|  |  |  |  | 1998-2003 | 1.86 | 1.05 | -0.81 | -43.55% | 1.76 |
|  |  |  |  | 2014-2019 | 2.42 | 2.62 | 0.20 | 8.26% | 0.92 |
|  |  |  |  | Change in time | 0.56 | 1.57 |  |  |  |
|  |  |  |  | (%) | 30.11% | 149.52% |  |  |  |
|  | **TOTAL** |  | F01-99 |  | 1 | 10 | Difference | (%) |  |
|  |  |  |  | 1998-2003 | 6.89 | 4.06 | -2.83 | -41.07% | 1.70 |
|  |  |  |  | 2014-2019 | 8.24 | 4.97 | -3.27 | -39.68% | 1.66 |
|  |  |  |  | Change in time | 1.35 | 0.91 |  |  |  |
|  |  |  |  | (%) | 19.59% | 22.41% |  |  |  |
| **Nervous** | **Episodic & paroxysmal** |  | G40-47 |  | 1 | 10 | Difference | (%) |  |
|  |  |  |  | 1998-2003 | 2.38 | 2.63 | 0.25 | 10.50% | 0.91 |
|  |  |  |  | 2014-2019 | 2.46 | 2.52 | 0.06 | 2.44% | 0.98 |
|  |  |  |  | Change in time | 0.08 | -0.11 |  |  |  |
|  |  |  |  | (%) | 3.36% | -4.18% |  |  |  |
|  | **Other** |  | G00-09; G50-64; G70-72; G80--83; G90-98 |  | 1 | 10 | Difference | (%) |  |
|  |  |  |  | 1998-2003 | 4.38 | 2.72 | -1.66 | -37.90% | 1.61 |
|  |  |  |  | 2014-2019 | 3.39 | 3.24 | -0.15 | -4.42% | 1.04 |
|  |  |  |  | Change in time | -0.99 | 0.52 |  |  |  |
|  |  |  |  | (%) | -22.60% | 19.12% |  |  |  |
|  | **Other degenerative** |  | G30-31 |  | 1 | 10 | Difference | (%) |  |
|  |  |  |  | 1998-2003 | 3.24 | 2.49 | -0.75 | -23.15% | 1.31 |
|  |  |  |  | 2014-2019 | 3.51 | 3.69 | 0.18 | 5.13% | 0.95 |
|  |  |  |  | Change in time | 0.27 | 1.20 |  |  |  |
|  |  |  |  | (%) | 8.33% | 48.19% |  |  |  |
|  | **Demyelinating diseases of CNS** |  | G35-37 |  | 1 | 10 | Difference | (%) |  |
|  |  |  |  | 1998-2003 | 1.53 | 2.38 | 0.85 | 55.56% | 0.64 |
|  |  |  |  | 2014-2019 | 1.71 | 2.37 | 0.66 | 38.60% | 0.72 |
|  |  |  |  | Change in time | 0.18 | -0.01 |  |  |  |
|  |  |  |  | (%) | 11.76% | -0.42% |  |  |  |
|  | **Extrapyramidal & movement** |  | G20-26 |  | 1 | 10 | Difference | (%) |  |
|  |  |  |  | 1998-2003 | 1.18 | 1.46 | 0.28 | 23.73% | 0.80 |
|  |  |  |  | 2014-2019 | 1.48 | 2.17 | 0.69 | 46.62% | 0.68 |
|  |  |  |  | Change in time | 0.30 | 0.71 |  |  |  |
|  |  |  |  | (%) | 25.42% | 48.63% |  |  |  |
|  | **Systemic atrophies of CNS** |  | G10-14 |  | 1 | 10 | Difference | (%) |  |
|  |  |  |  | 1998-2003 | 2.1 | 2.33 | 0.23 | 10.95% | 0.90 |
|  |  |  |  | 2014-2019 | 2.67 | 3.05 | 0.38 | 14.23% | 0.87 |
|  |  |  |  | Change in time | 0.57 | 0.72 |  |  |  |
|  |  |  |  | (%) | 27.14% | 30.90% |  |  |  |
|  | **TOTAL** |  | G00-98 |  | 1 | 10 | Difference | (%) |  |
|  |  |  |  | 1998-2003 | 11.98 | 9.31 | -2.67 | -22.29% | 1.29 |
|  |  |  |  | 2014-2019 | 12.59 | 12.4 | -0.19 | -1.51% | 1.02 |
|  |  |  |  | Change in time | 0.61 | 3.09 |  |  |  |
|  |  |  |  | (%) | 5.09% | 33.19% |  |  |  |
| **Circulatory** | **Hypertensive** |  | I10-15 |  | 1 | 10 | Difference | (%) |  |
|  |  |  |  | 1998-2003 | 3.58 | 1.33 | -2.25 | -62.85% | 1.91 |
|  |  |  |  | 2014-2019 | 1.61 | 1.06 | -0.55 | -34.16% | 1.35 |
|  |  |  |  | Change in time | -1.97 | -0.27 |  |  |  |
|  |  |  |  | (%) | -55.03% | -20.30% |  |  |  |
|  | **Ischaemic heart disease** | **Acute MI** | I21 |  | 1 | 10 | Difference | (%) |  |
|  |  |  |  | 1998-2003 | 23.75 | 15.79 | -7.96 | -33.52% | 23.75 |
|  |  |  |  | 2014-2019 | 11.88 | 4.14 | -7.74 | -65.15% | 11.88 |
|  |  |  |  | Change in time | -11.87 | -11.65 |  |  | -11.87 |
|  |  |  |  | (%) | -49.98% | -73.78% |  |  | -49.98% |
|  |  | **Chronic** | I25 |  | 1 | 10 | Difference | (%) |  |
|  |  |  |  | 1998-2003 | 11.26 | 5.43 | -5.83 | -51.78% | 2.07 |
|  |  |  |  | 2014-2019 | 5.46 | 1.92 | -3.54 | -64.84% | 2.84 |
|  |  |  |  | Change in time | -5.80 | -3.51 |  |  |  |
|  |  |  |  | (%) | -51.51% | -64.64% |  |  |  |
|  |  | **Other** | I20; I22-24 |  | 1 | 10 | Difference | (%) |  |
|  |  |  |  | 1998-2003 | 2.02 | 1.49 | -0.53 | -26.24% | 1.35 |
|  |  |  |  | 2014-2019 | 1.01 | 1.37 | 0.36 | 35.64% | 0.74 |
|  |  |  |  | Change in time | -1.01 | -0.12 |  |  |  |
|  |  |  |  | (%) | -50.00% | -8.05% |  |  |  |
|  |  | **Total** | I20-25 |  | 1 | 10 | Difference | (%) |  |
|  |  |  |  | 1998-2003 | 36.5 | 21.2 | -15.30 | -41.92% | 1.72 |
|  |  |  |  | 2014-2019 | 17.59 | 5.72 | -11.87 | -67.48% | 3.08 |
|  |  |  |  | Change in time | -18.91 | -15.48 |  |  |  |
|  |  |  |  | (%) | -51.81% | -73.02% |  |  |  |
|  | **Pulmonary** | **Other** | I27-28 |  | 1 | 10 | Difference | (%) |  |
|  |  |  |  | 1998-2003 | 1.46 | 0.73 | -0.73 | -50.00% | 2.02 |
|  |  |  |  | 2014-2019 | 1.25 | 0.85 | -0.40 | -32.00% | 1.48 |
|  |  |  |  | Change in time | -0.21 | 0.12 |  |  |  |
|  |  |  |  | (%) | -14.38% | 16.44% |  |  |  |
|  |  | **Pulmonary embolism** | I26 |  | 1 | 10 | Difference | (%) |  |
|  |  |  |  | 1998-2003 | 5.75 | 3.41 | -2.34 | -40.70% | 1.68 |
|  |  |  |  | 2014-2019 | 4.06 | 2.07 | -1.99 | -49.01% | 1.96 |
|  |  |  |  | Change in time | -1.69 | -1.34 |  |  |  |
|  |  |  |  | (%) | -29.39% | -39.30% |  |  |  |
|  |  | **Total** | I26-28 |  | 1 | 10 | Difference | (%) |  |
|  |  |  |  | 1998-2003 | 6.48 | 3.83 | -2.65 | -40.90% | 1.70 |
|  |  |  |  | 2014-2019 | 5.01 | 2.54 | -2.47 | -49.30% | 1.97 |
|  |  |  |  | Change in time | -1.47 | -1.29 |  |  |  |
|  |  |  |  | (%) | -22.69% | -33.68% |  |  |  |
|  | **Veins, lymph, vessels and nodes** |  | I80-89 |  | 1 | 10 | Difference | (%) |  |
|  |  |  |  | 1998-2003 | 2.1 | 1.54 | -0.56 | -26.67% | 1.36 |
|  |  |  |  | 2014-2019 | 1.62 | 0.77 | -0.85 | -52.47% | 2.11 |
|  |  |  |  | Change in time | -0.48 | -0.77 |  |  |  |
|  |  |  |  | (%) | -22.86% | -50.00% |  |  |  |
|  | **Other types of heart disease** | **Atrial fibrillation & flutter** | I48 |  | 1 | 10 | Difference | (%) |  |
|  |  |  |  | 1998-2003 | 2.46 | 1.46 | -1.00 | -40.65% | 1.69 |
|  |  |  |  | 2014-2019 | 1.64 | 1.08 | -0.56 | -34.15% | 1.51 |
|  |  |  |  | Change in time | -0.82 | -0.38 |  |  |  |
|  |  |  |  | (%) | -33.33% | -26.03% |  |  |  |
|  |  | **Cardiomyopathy** | I42 |  | 1 | 10 | Difference | (%) |  |
|  |  |  |  | 1998-2003 | 1 | 10 |  |  | 1.16 |
|  |  |  |  | 2014-2019 | 2.21 | 1.9 | -0.31 | -14.03% | 1.23 |
|  |  |  |  | Change in time | 1.5 | 1.22 | -0.28 | -18.67% |  |
|  |  |  |  | (%) | -0.71 | -0.68 |  |  |  |
|  |  | **Heart failure** | I50 |  | 1 | 10 | Difference | (%) |  |
|  |  |  |  | 1998-2003 | 8 | 4.09 | -3.91 | -48.88% | 1.96 |
|  |  |  |  | 2014-2019 | 4.47 | 3.31 | -1.16 | -25.95% | 1.35 |
|  |  |  |  | Change in time | -3.53 | -0.78 |  |  |  |
|  |  |  |  | (%) | -44.13% | -19.07% |  |  |  |
|  |  | **Other** | I30-40; I43-47; I49; I51 |  | 1 | 10 | Difference | (%) |  |
|  |  |  |  | 1998-2003 | 16.6 | 9.05 | -7.55 | -45.48% | 1.83 |
|  |  |  |  | 2014-2019 | 10.26 | 5.16 | -5.10 | -49.71% | 1.99 |
|  |  |  |  | Change in time | -6.34 | -3.89 |  |  |  |
|  |  |  |  | (%) | -38.19% | -42.98% |  |  |  |
|  |  | **Total** | I30-51 |  | 1 | 10 | Difference | (%) |  |
|  |  |  |  | 1998-2003 | 27.53 | 14.73 | -12.80 | -46.49% | 1.87 |
|  |  |  |  | 2014-2019 | 16.92 | 9.39 | -7.53 | -44.50% | 1.80 |
|  |  |  |  | Change in time | -10.61 | -5.34 |  |  |  |
|  |  |  |  | (%) | -38.54% | -36.25% |  |  |  |
|  | **Stroke** | **Haemorrhage** | I60-62 |  | 1 | 10 | Difference | (%) |  |
|  |  |  |  | 1998-2003 | 10.93 | 6.84 | -4.09 | -37.42% | 1.60 |
|  |  |  |  | 2014-2019 | 6.79 | 3.98 | -2.81 | -41.38% | 1.71 |
|  |  |  |  | Change in time | -4.14 | -2.86 |  |  |  |
|  |  |  |  | (%) | -37.88% | -41.81% |  |  |  |
|  |  | **Infarction** | I63 |  | 1 | 10 | Difference | (%) |  |
|  |  |  |  | 1998-2003 | 2.46 | 2.87 | 0.41 | 16.67% | 0.86 |
|  |  |  |  | 2014-2019 | 2.39 | 2.05 | -0.34 | -14.23% | 1.17 |
|  |  |  |  | Change in time | -0.07 | -0.82 |  |  |  |
|  |  |  |  | (%) | -2.85% | -28.57% |  |  |  |
|  |  | **Other** | I64-69 |  | 1 | 10 | Difference | (%) |  |
|  |  |  |  | 1998-2003 | 11.38 | 6.97 | -4.41 | -38.75% | 1.63 |
|  |  |  |  | 2014-2019 | 5.72 | 2.9 | -2.82 | -49.30% | 1.97 |
|  |  |  |  | Change in time | -5.66 | -4.07 |  |  |  |
|  |  |  |  | (%) | -49.74% | -58.39% |  |  |  |
|  |  | **Total** | I60-69 |  | 1 | 10 | Difference | (%) |  |
|  |  |  |  | 1998-2003 | 23.97 | 15.39 | -8.58 | -35.79% | 1.56 |
|  |  |  |  | 2014-2019 | 14.17 | 7.58 | -6.59 | -46.51% | 1.87 |
|  |  |  |  | Change in time | -9.80 | -7.81 |  |  |  |
|  |  |  |  | (%) | -40.88% | -50.75% |  |  |  |
|  | **Arteries** | **Aortic aneurysm & dis.** | I71 |  | 1 | 10 | Difference | (%) |  |
|  |  |  |  | 1998-2003 | 1.91 | 1.55 | -0.36 | -18.85% | 1.23 |
|  |  |  |  | 2014-2019 | 1.72 | 1.59 | -0.13 | -7.56% | 1.08 |
|  |  |  |  | Change in time | -0.19 | 0.04 |  |  |  |
|  |  |  |  | (%) | -9.95% | 2.58% |  |  |  |
|  |  | **Other** | I70; I72-79 |  | 1 | 10 | Difference | (%) |  |
|  |  |  |  | 1998-2003 | 2.97 | 1.57 | -1.40 | -47.14% | 1.90 |
|  |  |  |  | 2014-2019 | 2.13 | 1.57 | -0.56 | -26.29% | 1.36 |
|  |  |  |  | Change in time | -0.84 | 0.00 |  |  |  |
|  |  |  |  | (%) | -28.28% | 0.00% |  |  |  |
|  |  | **Total** | I70-79 |  | 1 | 10 | Difference | (%) |  |
|  |  |  |  | 1998-2003 | 4.41 | 2.46 | -1.95 | -44.22% | 1.79 |
|  |  |  |  | 2014-2019 | 3.22 | 2.03 | -1.19 | -36.96% | 1.58 |
|  |  |  |  | Change in time | -1.19 | -0.43 |  |  |  |
|  |  |  |  | (%) | -26.98% | -17.48% |  |  |  |
|  | **Other** |  | I00-09; I95-99 |  | 1 | 10 | Difference | (%) |  |
|  |  |  |  | 1998-2003 | 2.05 | 1.21 | -0.84 | -40.98% | 1.69 |
|  |  |  |  | 2014-2019 | 1.23 | 1.13 | -0.10 | -8.13% | 1.09 |
|  |  |  |  | Change in time | -0.82 | -0.08 |  |  |  |
|  |  |  |  | (%) | -40.00% | -6.61% |  |  |  |
|  | **TOTAL** |  | I00-99 |  | 1 | 10 | Difference | (%) |  |
|  |  |  |  | 1998-2003 | 104.35 | 57.53 | -46.82 | -44.87% | 1.81 |
|  |  |  |  | 2014-2019 | 59.51 | 24.99 | -34.52 | -58.01% | 2.38 |
|  |  |  |  | Change in time | -44.84 | -32.54 |  |  |  |
|  |  |  |  | (%) | -42.97% | -56.56% |  |  |  |
| **Respiratory** | **Chronic lung disease** | **Asthma** | J45 |  | 1 | 10 | Difference | (%) |  |
|  |  |  |  | 1998-2003 | 2.78 | 1.16 | -1.62 | -58.27% | 2.09 |
|  |  |  |  | 2014-2019 | 1.26 | 1.05 | -0.21 | -16.67% | 1.20 |
|  |  |  |  | Change in time | -1.52 | -0.11 |  |  |  |
|  |  |  |  | (%) | -54.68% | -9.48% |  |  |  |
|  |  | **Other (COPD)** | J40-44; J46-47 |  | 1 | 10 | Difference | (%) |  |
|  |  |  |  | 1998-2003 | 16.57 | 4.94 | -11.63 | -70.19% | 3.35 |
|  |  |  |  | 2014-2019 | 21.22 | 6.14 | -15.08 | -71.07% | 3.45 |
|  |  |  |  | Change in time | 4.65 | 1.20 |  |  |  |
|  |  |  |  | (%) | 28.06% | 24.29% |  |  |  |
|  |  | **Total** | J40-47 |  | 1 | 10 | Difference | (%) |  |
|  |  |  |  | 1998-2003 | 18.99 | 5.2 | -13.79 | -72.62% | 3.65 |
|  |  |  |  | 2014-2019 | 22.05 | 6.57 | -15.48 | -70.20% | 3.36 |
|  |  |  |  | Change in time | 3.06 | 1.37 |  |  |  |
|  |  |  |  | (%) | 16.11% | 26.35% |  |  |  |
|  | **Influenza & pneumonia** |  | J09-J18 |  | 1 | 10 | Difference | (%) |  |
|  |  |  |  | 1998-2003 | 8.16 | 5.65 | -2.51 | -30.76% | 1.45 |
|  |  |  |  | 2014-2019 | 6.57 | 3.24 | -3.33 | -50.68% | 2.03 |
|  |  |  |  | Change in time | -1.59 | -2.41 |  |  |  |
|  |  |  |  | (%) | -19.49% | -42.65% |  |  |  |
|  | **External agents** |  | J60-70 |  | 1 | 10 | Difference | (%) |  |
|  |  |  |  | 1998-2003 | 1.66 | 2.01 | 0.35 | 21.08% | 1.18 |
|  |  |  |  | 2014-2019 | 1.62 | 1.03 | -0.59 | -36.42% | 1.56 |
|  |  |  |  | Change in time | -0.04 | -0.98 |  |  |  |
|  |  |  |  | (%) | -2.41% | -48.76% |  |  |  |
|  | **Other** |  | J00-08; J20-22; J30-39; J80-99 |  | 1 | 10 | Difference | (%) |  |
|  |  |  |  | 1998-2003 | 5.4 | 2.56 | -2.84 | -52.59% | 2.11 |
|  |  |  |  | 2014-2019 | 6.05 | 2.63 | -3.42 | -56.53% | 2.31 |
|  |  |  |  | Change in time | 0.65 | 0.07 |  |  |  |
|  |  |  |  | (%) | 12.04% | 2.73% |  |  |  |
|  | **TOTAL** |  | J00-99 |  | 1 | 10 | Difference | (%) |  |
|  |  |  |  | 1998-2003 | 32.81 | 12.88 | -19.93 | -60.74% | 2.55 |
|  |  |  |  | 2014-2019 | 34.97 | 11.97 | -23.00 | -65.77% | 2.92 |
|  |  |  |  | Change in time | 2.16 | -0.91 |  |  |  |
|  |  |  |  | (%) | 6.58% | -7.07% |  |  |  |
| **Digestive** | **Liver** | **Alcoholic liver disease** | K70 |  | 1 | 10 | Difference | (%) |  |
|  |  |  |  | 1998-2003 | 10.09 | 2.87 | -7.22 | -71.56% | 3.52 |
|  |  |  |  | 2014-2019 | 6.42 | 2.94 | -3.48 | -54.21% | 2.19 |
|  |  |  |  | Change in time | -3.67 | 0.07 |  |  |  |
|  |  |  |  | (%) | -36.37% | 2.44% |  |  |  |
|  |  | **Fibrosis and cirrhosis** | K74 |  | 1 | 10 | Difference | (%) |  |
|  |  |  |  | 1998-2003 | 3.89 | 2.28 | -1.61 | -41.39% | 1.71 |
|  |  |  |  | 2014-2019 | 4.84 | 2.06 | -2.78 | -57.44% | 2.35 |
|  |  |  |  | Change in time | 0.95 | -0.22 |  |  |  |
|  |  |  |  | (%) | 24.42% | -9.65% |  |  |  |
|  |  | **Other** | K71-73; K75-76 |  | 1 | 10 | Difference | (%) |  |
|  |  |  |  | 1998-2003 | 1.64 | 1.33 | -0.31 | -18.90% | 1.23 |
|  |  |  |  | 2014-2019 | 1.76 | 1.79 | 0.03 | 1.70% | 0.99 |
|  |  |  |  | Change in time | 0.12 | 0.46 |  |  |  |
|  |  |  |  | (%) | 7.32% | 34.59% |  |  |  |
|  |  | **Total** | K70-77 |  | 1 | 10 | Difference | (%) |  |
|  |  |  |  | 1998-2003 | 15.18 | 4.59 | -10.59 | -69.76% | 3.30 |
|  |  |  |  | 2014-2019 | 12.74 | 5.02 | -7.72 | -60.60% | 2.54 |
|  |  |  |  | Change in time | -2.44 | 0.43 |  |  |  |
|  |  |  |  | (%) | -16.07% | 9.37% |  |  |  |
|  | **Oesophagus, stomach, duodenum** |  | K20-31 |  | 1 | 10 | Difference | (%) |  |
|  |  |  |  | 1998-2003 | 1.96 | 0.95 | -1.01 | -51.53% | 2.08 |
|  |  |  |  | 2014-2019 | 1.46 | 0.95 | -0.51 | -34.93% | 1.53 |
|  |  |  |  | Change in time | -0.50 | 0.00 |  |  |  |
|  |  |  |  | (%) | -25.51% | 0.00% |  |  |  |
|  | **Gallbladder, biliary tract, pancreas** |  | K80-87 |  | 1 | 10 | Difference | (%) |  |
|  |  |  |  | 1998-2003 | 1.96 | 0.95 | -1.01 | -51.53% | 1.44 |
|  |  |  |  | 2014-2019 | 1.46 | 0.95 | -0.51 | -34.93% | 1.17 |
|  |  |  |  | Change in time | -0.50 | 0.00 |  |  |  |
|  |  |  |  | (%) | -25.51% | 0.00% |  |  |  |
|  | **Other** |  | K00-14; K35-38, K40-46; K65-66; K90-93 |  | 1 | 10 | Difference | (%) |  |
|  |  |  |  | 1998-2003 | 3.13 | 1.9 | -1.23 | -39.30% | 1.65 |
|  |  |  |  | 2014-2019 | 2.93 | 1.57 | -1.36 | -46.42% | 1.88 |
|  |  |  |  | Change in time | -0.20 | -0.33 |  |  |  |
|  |  |  |  | (%) | -6.39% | -17.37% |  |  |  |
|  | **Other intestines** |  | K50-64 |  | 1 | 10 | Difference | (%) |  |
|  |  |  |  | 1998-2003 | 5.55 | 3.37 | -2.18 | -39.28% | 1.65 |
|  |  |  |  | 2014-2019 | 5.43 | 2.58 | -2.85 | -52.49% | 2.11 |
|  |  |  |  | Change in time | -0.12 | -0.79 |  |  |  |
|  |  |  |  | (%) | -2.16% | -23.44% |  |  |  |
|  | **TOTAL** |  | K00-93 |  | 1 | 10 | Difference | (%) |  |
|  |  |  |  | 1998-2003 | 26.02 | 9.39 | -16.63 | -63.91% | 2.77 |
|  |  |  |  | 2014-2019 | 22.6 | 9.52 | -13.08 | -57.88% | 2.37 |
|  |  |  |  | Change in time | -3.42 | 0.13 |  |  |  |
|  |  |  |  | (%) | -13.14% | 1.38% |  |  |  |
| **Skin and subcutaneous tissue** | **TOTAL** |  | L00-L99 |  | 1 | 10 | Difference | (%) |  |
|  |  |  |  | 1998-2003 | 1.22 | 1 | -0.22 | -18.03% | 1.22 |
|  |  |  |  | 2014-2019 | 1.26 | 0.56 | -0.70 | -55.56% | 2.24 |
|  |  |  |  | Change in time | 0.04 | -0.44 |  |  |  |
|  |  |  |  | (%) | 3.28% | -44.00% |  |  |  |
| **Musculoskeletal** | **TOTAL** |  | M00-99 |  | 1 | 10 | Difference | (%) |  |
|  |  |  |  | 1998-2003 | 3.11 | 2.6 | -0.51 | -16.40% | 1.19 |
|  |  |  |  | 2014-2019 | 2.59 | 1.44 | -1.15 | -44.40% | 1.80 |
|  |  |  |  | Change in time | -0.52 | -1.16 |  |  |  |
|  |  |  |  | (%) | -16.72% | -44.62% |  |  |  |
| **Genitourinary system** | **Other** |  | N00-16; N20-99 |  | 1 | 10 | Difference | (%) |  |
|  |  |  |  | 1998-2003 | 2.38 | 1.48 | -0.90 | -37.82% | 1.61 |
|  |  |  |  | 2014-2019 | 3.71 | 1.51 | -2.20 | -59.30% | 2.44 |
|  |  |  |  | Change in time | 1.33 | 0.03 |  |  |  |
|  |  |  |  | (%) | 55.88% | 2.03% |  |  |  |
|  | **Renal failure** |  | N17-19 |  | 1 | 10 | Difference | (%) |  |
|  |  |  |  | 1998-2003 | 3.13 | 1.57 | -1.56 | -49.84% | 1.99 |
|  |  |  |  | 2014-2019 | 2.75 | 1.45 | -1.30 | -47.27% | 1.90 |
|  |  |  |  | Change in time | -0.38 | -0.12 |  |  |  |
|  |  |  |  | (%) | -12.14% | -7.64% |  |  |  |
|  | **TOTAL** |  | N00-99 |  | 1 | 10 | Difference | (%) |  |
|  |  |  |  | 1998-2003 | 4.87 | 2.4 | -2.47 | -50.72% | 2.03 |
|  |  |  |  | 2014-2019 | 5.91 | 2.64 | -3.27 | -55.33% | 2.23 |
|  |  |  |  | Change in time | 1.04 | 0.24 |  |  |  |
|  |  |  |  | (%) | 21.36% | 10.00% |  |  |  |
| **Symptoms, signs,  abnormal findings** | **TOTAL** |  | R00-99 |  | 1 | 10 | Difference | (%) |  |
|  |  |  |  | 1998-2003 | 25.45 | 7.9 | -17.55 | -68.96% | 3.22 |
|  |  |  |  | 2014-2019 | 24.09 | 8.12 | -15.97 | -66.29% | 2.97 |
|  |  |  |  | Change in time | -1.36 | 0.22 |  |  |  |
|  |  |  |  | (%) | -5.34% | 2.78% |  |  |  |
| **External** | **Undetermined intent** |  | Y10-34 |  | 1 | 10 | Difference | (%) |  |
|  |  |  |  | 1998-2003 | 2.53 | 0.49 | -2.04 | -80.63% | 5.11 |
|  |  |  |  | 2014-2019 | 3.4 | 3.51 | 0.11 | 3.24% | 0.97 |
|  |  |  |  | Change in time | 0.87 | 3.02 |  |  |  |
|  |  |  |  | (%) | 34.39% | 616.33% |  |  |  |
|  | **Accidents** | **Drugs, alcohol poisoning** | X41-42; X44-45 |  | 1 | 10 | Difference | (%) |  |
|  |  |  |  | 1998-2003 | 1.66 | 0.31 | -1.35 | -81.33% | 5.39 |
|  |  |  |  | 2014-2019 | 2.09 | 1.17 | -0.92 | -44.02% | 1.78 |
|  |  |  |  | Change in time | 0.43 | 0.86 |  |  |  |
|  |  |  |  | (%) | 25.90% | 277.42% |  |  |  |
|  |  | **Other** | X00- |  | 1 | 10 | Difference | (%) |  |
|  |  |  |  | 1998-2003 | 8.88 | 3.34 | -5.54 | -62.39% | 2.66 |
|  |  |  |  | 2014-2019 | 10.92 | 4.26 | -6.66 | -60.99% | 2.57 |
|  |  |  |  | Change in time | 2.04 | 0.92 |  |  |  |
|  |  |  |  | (%) | 22.97% | 27.54% |  |  |  |
|  |  | **Vehicle accidents** | V01-06; V09-34; V38-44; V46-55;  V58-67; V69-79; V81-82; V84-85; V87-89 |  | 1 | 10 | Difference | (%) |  |
|  |  |  |  | 1998-2003 | 5.99 | 4.96 | -1.03 | -17.20% | 1.21 |
|  |  |  |  | 2014-2019 | 2.57 | 4.35 | 1.78 | 69.26% | 0.59 |
|  |  |  |  | Change in time | -3.42 | -0.61 |  |  |  |
|  |  |  |  | (%) | -57.10% | -12.30% |  |  |  |
|  |  | **Total** | X00-59; V00-97; W00-98 |  | 1 | 10 | Difference | (%) |  |
|  |  |  |  | 1998-2003 | 15.21 | 7.65 | -7.56 | -49.70% | 1.99 |
|  |  |  |  | 2014-2019 | 14.38 | 6.75 | -7.63 | -53.06% | 2.13 |
|  |  |  |  | Change in time | -0.83 | -0.90 |  |  |  |
|  |  |  |  | (%) | -5.46% | -11.76% |  |  |  |
|  | **Other** |  | X85-99; Y00-09; Y35-36; Y85-98 |  | 1 | 10 | Difference | (%) |  |
|  |  |  |  | 1998-2003 |  | 1.93 | -1.39 | -41.87% | 1.72 |
|  |  |  |  | 2014-2019 | 2.31 | 2.3 | -0.01 | -0.43% | 0.66 |
|  |  |  |  | Change in time | -1.01 | 0.37 |  |  |  |
|  |  |  |  | (%) | -30.42% | 19.17% |  |  |  |
|  | **Intentional self-harm** |  | X60-84 |  | 1 | 10 | Difference | (%) |  |
|  |  |  |  | 1998-2003 | 11.23 | 6.02 | -5.21 | -46.39% | 1.86 |
|  |  |  |  | 2014-2019 | 9.94 | 8.35 | -1.59 | -16.00% | -2.04 |
|  |  |  |  | Change in time | -1.29 | 2.33 |  |  |  |
|  |  |  |  | (%) | -11.49% | 38.70% | -11.49% |  |  |
|  | **TOTAL** |  | X00-99; V00-97; W00-98; Y00-98 |  | 1 | 10 | Difference | (%) |  |
|  |  |  |  | 1998-2003 | 30.61 | 13.67 | -16.94 | -55.34% | 2.24 |
|  |  |  |  | 2014-2019 | 29.11 | 15.61 | -13.50 | -46.38% | 1.87 |
|  |  |  |  | Change in time | -1.50 | 1.94 |  |  |  |
|  |  |  |  | (%) | -4.90% | 14.19% |  |  |  |
| **Congenital** | **TOTAL** |  | Q00-99 |  | 1 | 10 | Difference | (%) |  |
|  |  |  |  | 1998-2003 | 2.12 | 2.32 | 0.20 | 9.43% | 0.91 |
|  |  |  |  | 2014-2019 | 2.08 | 3.02 | 0.94 | 45.19% | 0.69 |
|  |  |  |  | Change in time | -0.04 | 0.70 |  |  |  |
|  |  |  |  | (%) | -1.89% | 30.17% |  |  |  |
| **Other** | **TOTAL** |  | P00-96; O00-99; H05-82; Z05-ZZ; S18; T32-98; U00-49; U83-85 |  | 1 | 10 | Difference | (%) |  |
|  |  |  |  | 1998-2003 | 5.34 | 6.71 | 1.37 | 25.66% | 0.80 |
|  |  |  |  | 2014-2019 | 0.7 | 0.5 | -0.20 | -28.57% | 1.42 |
|  |  |  |  | Change in time | -4.64 | -6.21 |  |  |  |
|  |  |  |  | (%) | -86.89% | -92.55% |  |  |  |
| **ALL-CAUSE** | **TOTAL** |  |  |  | 1 | 10 | Difference | (%) |  |
|  |  |  |  | 1998-2003 | 413.95 | 246.97 | -166.98 | -40.34% | 1.71 |
|  |  |  |  | 2014-2019 | 349.91 | 183.86 | -166.05 | -47.46% | 1.96 |
|  |  |  |  | Change in time in time | -64.04 | -63.11 |  |  |  |
|  |  |  |  | (%) | -15.47% | -25.55% |  |  |  |

## Overall and cause-specific premature mortality attributable to socioeconomic inequality in Belgium


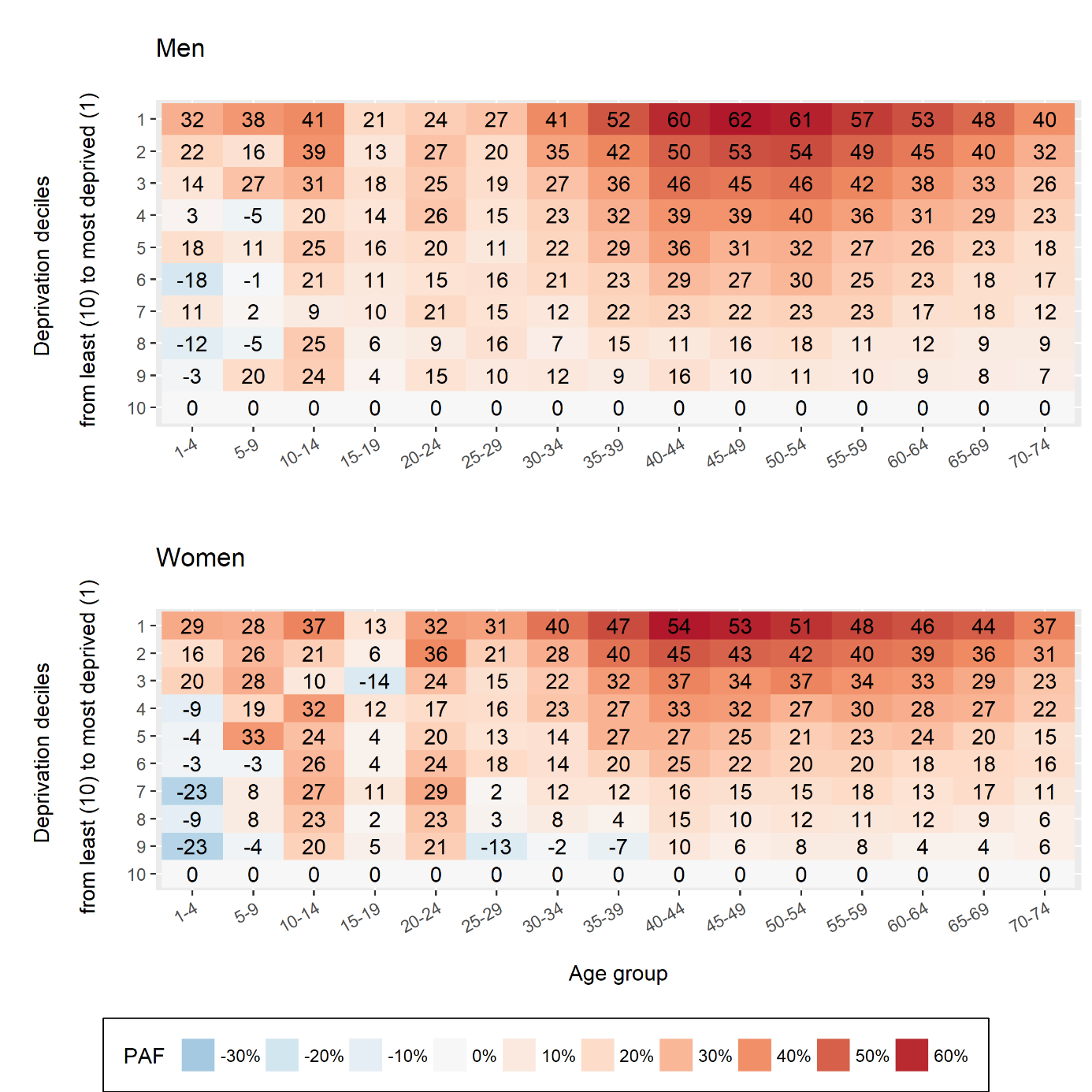


*Figure S4 Proportion of premature mortality attributable to inequality in men and women between 1998-2019*

*Table S4 Overview of mortality attributable to inequality in Belgium from 1998 to 2019*

| **Chapter** | **Subgroup** | **Diagnosis** | **ICD 10 codes** | | **Observed**  **Deaths** | **Expected deaths** | **Excess deaths** | **%**  **of excess deaths** | **PAF (95% CI)** |
| --- | --- | --- | --- | --- | --- | --- | --- | --- | --- |
| Infections | Viral hepatitis |  | B15-B19 | | 1,118 | 993 | 125 | 0.07% | 11.22 (-12.28 to 33.1) |
|  | Other |  | A0-B09; B20-B99 | | 13,839 | 9,279 | 4,560 | 2.71% | 32.95 (28.31 to 37.55) |
|  | TOTAL |  | A01-99; B00-99 | | 14,982 | 9,879 | 5,103 | 3.03% | 34.06 (31.05 to 39.24) |
| Neoplasm | Respiratory | Larynx | C32 | | 3,380 | 2,654 | 726 | 0.43% | 23.3 (14.54 to 32.62) |
|  |  | Lung | C34 | | 84,012 | 63,579 | 20,433 | 12.14% | 24.44 (22.62 to 26.24) |
|  |  | Other | C30-31; C33; C35-39 | | 1,775 | 1,661 | 114 | 0.07% | 6.42 (4.85 to 25.11) |
|  |  | Total | C30-39 | | 89,191 | 66,928 | 22,263 | 13.23% | 24.97 (23.22 to 26.72) |
|  | Mouth |  | C00-14 | | 8,842 | 6,508 | 2,334 | 1.39% | 26.39 (20.51 to 32.14) |
|  | Other |  | C76-80; C97; D00-D48 | | 22,774 | 15,613 | 7,161 | 4.25% | 31.44 (28.05 to 34.8) |
|  | Urinary | Bladder | C67 | | 6,527 | 5,831 | 696 | 0.41% | 11.8 (5.68 to 18.4) |
|  |  | Kidney | C64 | | 5,829 | 5,681 | 148 | 0.09% | 2.54 (1.88 to 12.16) |
|  |  | Other | C65-66; C68 | | 1,411 | 1,290 | 121 | 0.07% | 16.18 (6.28 to 27.35) |
|  |  | Total | C64-68 | | 13,827 | 12,342 | 1,485 | 0.88% | 10.74 (5.91 to 15.54) |
|  | Digestive | Colon | C18 | | 19,776 | 18,702 | 1,074 | 0.64% | 6.48 (2.97 to 10.27) |
|  |  | Liver & IH bile ducts | C22 | | 8,876 | 6,453 | 2,423 | 1.44% | 28.08 (22.57 to 33.54) |
|  |  | Oesophagus | C15 | | 9,520 | 8,652 | 868 | 0.52% | 11.47 (6.98 to 16.39) |
|  |  | Other | C17; C21; C23; C24; C26 | | 5,382 | 5,438 | -56 | -0.03% | -1.05 (-1.47 to 10.57) |
|  |  | Pancreas | C25 | | 16,478 | 14,405 | 2,073 | 1.23% | 12.99 (8.96 to 17.18) |
|  |  | Rectosigmoid junction | C19 | | 1,306 | 1,259 | 47 | 0.03% | 3.57 (2.51 to 25) |
|  |  | Rectum | C20 | | 5,101 | 4,829 | 272 | 0.16% | 8.25 (2.89 to 14.75) |
|  |  | Stomach | C16 | | 8,014 | 7,130 | 884 | 0.53% | 12.62 (7.16 to 18.59) |
|  |  | Total | C15-26 | | 74,549 | 65,023 | 9,526 | 5.66% | 12.78 (10.71 to 14.83) |
|  | Female genital organs |  | C51-58 | | 14,109 | 12,651 | 1,458 | 0.87% | 10.34 (5.26 to 15.32) |
|  | Thyroid & endocrine glands |  | C73-75 | | 1,565 | 1,629 | -64 | -0.04% | -4.17 (-20.19 to 11.45) |
|  | Male genital organs |  | C60-63 | | 9,109 | 7,897 | 1,212 | 0.72% | 13.29 (7.27 to 19.27) |
|  | Lymphoid & haematopoietic | Leukaemia, unspecified unspecified | | C95 | 1,916 | 1,987 | -71 | -0.04% | -4.3 (-1.95 to 18.46) |
|  |  | Lymphoid leukaemia | C91 | | 1,805 | 1,989 | -184 | -0.11% | 8.25 (2.25 to 16.65) |
|  |  | Multiple myeloma | C90 | | 3,899 | 3,985 | -86 | -0.05% | 4.1 (0.34 to 10.15) |
|  |  | Myeloid leukaemia | C92 | | 4,492 | 4,207 | 285 | 0.17% | 6.34 (5.26 to 18.71) |
|  |  | Other | C81-89; C93-94;C96 | | 7,614 | 6,963 | 651 | 0.39% | 8.55 (7.78 to 18.35) |
|  |  | Total | C81-96 | | 19,794 | 18,338 | 1,456 | 0.86% | 7.35 (3.19 to 11.49) |
|  | Bone and articular cartilage |  | C40-41 | | 1,166 | 1,209 | -43 | -0.03% | -3.69 (-24.06 to 15.58) |
|  | Mesothelial and soft tissue |  | C45-49 | | 4,796 | 5,579 | -783 | -0.47% | -16.32 (-25.76 to -7.1) |
|  | Breast |  | C50 | | 27,994 | 28,198 | -204 | -0.12% | -0.73 (-4.34 to 2.83) |
|  | Eye, brain & CNS |  | C69-72 | | 10,598 | 11,773 | -1,175 | -0.70% | -11.08 (-17.18 to -5.07) |
|  | Skin |  | C43-44 | | 4,760 | 5,350 | -590 | -0.35% | -12.4 (-21.48 to -3.5) |
|  | TOTAL |  | C00-97; D00-48 | | 303,241 | 257,272 | 45,969 | 27.31% | 15.33 (14.33 to 16.32) |
| Blood | TOTAL |  | D50-89 | | 2,044 | 1,842 | 156 | 0.09% | 7.82 (-9.82 to 24.5) |
| Endocrine, nutritional, metabolic | Diabetes mellitus |  | E10-14 | | 10,136 | 6,459 | 3,677 | 2.18% | 36.27 (30.45 to 41.9) |
|  | Metabolic disorders |  | E70-89 | | 3,059 | 2,621 | 438 | 0.26% | 14.29 (-0.61 to 28) |
|  | Obesity |  | E66-68 | | 1,675 | 1,374 | 301 | 0.18% | 17.93 (-3.49 to 38.13) |
|  | Other |  | E00-07; E15-63 | | 1,055 | 1,004 | 51 | 0.03% | 4.9 (-25.6 to 31.83) |
|  | TOTAL |  | E00-E89 | | 16,027 | 9,527 | 6,500 | 3.86% | 41.9 (38.09 to 45.68) |
| Mental behavioural | Psychoactive substance use | Alcohol | F10 | | 6,408 | 3,340 | 3,068 | 1.82% | 48.85 (42.3 to 55.21) |
|  |  | Other | F11-19 | | 1,380 | 1,129 | 251 | 0.15% | 18.17 (14.86 to 50.57) |
|  |  | Total | F10-19 | | 7,798 | 3,828 | 3,970 | 2.36% | 50.92 (44.85 to 56.79) |
|  | Organic |  | F01-F09 | | 4,348 | 3,988 | 360 | 0.21% | 8.29 (-1.16 to 17.52) |
|  | Other |  | F20-99 | | 2,054 | 1,794 | 260 | 0.15% | 12.63 (-6.29 to 30.46) |
|  | TOTAL |  | F01-99 | | 14,215 | 8,275 | 5,940 | 3.53% | 42.21 (38.06 to 46.34) |
| Nervous | Episodic & paroxysmal |  | G40-47 | | 3,373 | 2,774 | 599 | 0.36% | 17.73 (5.14 to 29.72) |
|  | Other |  | G00-09; G50-64; G70-72;  G80--83; G90-98 | | 5,647 | 4,587 | 1,060 | 0.63% | 18.77 (10.34 to 27.07) |
|  | Other degenerative |  | G30-31 | | 5,203 | 4,998 | 205 | 0.12% | 3.95 (-4.54 to 12.2) |
|  | Demyelinating diseases of CNS |  | G35-37 | | 2,107 | 2,242 | -135 | -0.08% | -6.4 (-20.15 to 7.02) |
|  | Extrapyramidal & movement |  | G20-26 | | 3,248 | 3,335 | -87 | -0.05% | -2.65 (-13.88 to 8.35) |
|  | Systemic atrophies of CNS |  | G10-14 | | 4,558 | 5,080 | -522 | -0.31% | -11.47 (-21.29 to -1.91) |
|  | TOTAL |  | G00-98 | | 24,240 | 21,459 | 2,781 | 1.65% | 12.6 (9.32 to 16.06) |
| Circulatory | Hypertensive |  |  | |  |  |  |  |  |
|  |  | Hypertensive heart disease | I11 | | 1,011 | 830 | 181 | 0.11% | -17.9 (-6.81 to 40.89) |
|  |  | Total | I10-15 | | 2,486 | 1,793 | 693 | 0.41% | 27.85 (15.25 to 39.92) |
|  | Ischaemic heart disease | Acute MI | I21 | | 46,961 | 35,961 | 11,000 | 6.54% | 23.7 (21.26 to 26.13) |
|  |  | Chronic | I25 | | 19,398 | 14,369 | 5,029 | 2.99% | 26.22 (22.34 to 30.04) |
|  |  | Other | I20; I22-24 | | 2,986 | 2,462 | 524 | 0.31% | 19.94 (10.3 to 29.79) |
|  |  | Total | I20-25 | | 69,357 | 51,304 | 18,053 | 10.73% | 26.02 (24.01 to 28.01) |
|  | Pulmonary | Other | I27-28 | | 845 | 903 | -58 | -0.03% | -6.81 (-11.85 to 26.5) |
|  |  | Pulmonary embolism | I26 | | 5,609 | 4,031 | 1,578 | 0.94% | 30.95 (23.98 to 37.82) |
|  |  | Total | I26-28 | | 6,482 | 4,726 | 1,756 | 1.04% | 27.09 (19.6 to 34.35) |
|  | Veins, lymph, vessels and nodes |  | I80-89 | | 1,510 | 1,247 | 263 | 0.16% | 17.44 (-1.41 to 35.28) |
|  | Other types of heart disease | Atrial fibrillation & flutter | I48 | | 2,509 | 2,124 | 385 | 0.23% | 19.13 (9.13 to 29.4) |
|  |  | Cardiomyopathy | I42 | | 3,491 | 2,999 | 492 | 0.29% | 18.12 (9.8 to 26.84) |
|  |  | Heart failure | I50 | | 11,277 | 8,029 | 3,248 | 1.93% | 29.84 (24.86 to 34.77) |
|  |  | Other | I30-40; I43-47; I49; I51 | | 26,935 | 20,883 | 6,052 | 3.60% | 23.06 (19.86 to 26.26) |
|  |  | Total | I30-51 | | 44,324 | 32,400 | 11,924 | 7.08% | 26.91 (24.39 to 29.39) |
|  | Stroke | Haemorrhage | I60-62 | | 14,994 | 11,956 | 3,038 | 1.80% | 21.2 (16.82 to 25.55) |
|  |  | Infarction | I63 | | 4,445 | 4,079 | 366 | 0.22% | 10.87 (4.15 to 18.76) |
|  |  | Other | I64-69 | | 14,661 | 11,352 | 3,309 | 1.97% | 23.01 (18.6 to 27.41) |
|  |  | Total | I60-69 | | 34,132 | 26,591 | 7,541 | 4.48% | 22.1 (19.16 to 25.06) |
|  | Arteries | Aortic aneurysm & dis. | I71 | | 4,516 | 4,361 | 155 | 0.09% | 10.8 (4.24 to 17.55) |
|  |  | Other | I70; I72-79 | | 19,592 | 12,252 | 7,340 | 4.36% | 38.1 (34.66 to 41.55) |
|  |  | Total | I70-79 | | 8,984 | 7,362 | 1,622 | 0.96% | 18.06 (11.53 to 24.43) |
|  | Other |  | I00-09; I95-99 | | 1,453 | 1,169 | 284 | 0.17% | 19.56 (2.24 to 35.78) |
|  | TOTAL |  | I00-99 | | 169,965 | 122,860 | 47,105 | 27.99% | 27.78 (26.53 to 29.02) |
| Respiratory | Chronic lung disease | Asthma | J45 | | 1,369 | 1,093 | 276 | 0.16% | 27.2 (10.5 to 45.84) |
|  |  | Other (COPD) | J40-44; J46-47 | | 32,373 | 17,400 | 14,973 | 8.90% | 46.34 (43.78 to 48.86) |
|  |  | Total | J40-47 | | 33,765 | 17,887 | 15,878 | 9.43% | 47.02 (44.51 to 49.49) |
|  | Influenza & pneumonia |  | J09-J18 | | 13,412 | 9,184 | 4,228 | 2.51% | 31.52 (26.9 to 36.08) |
|  | External agents |  | J60-70 | | 3,486 | 2,561 | 925 | 0.55% | 26.54 (15.91 to 36.71) |
|  | Other |  | J00-08; J20-22; J30-39; J80-99 | | 8,728 | 6,281 | 2,447 | 1.45% | 28.02 (21.65 to 34.17) |
|  | TOTAL |  | J00-99 | | 59,567 | 34,523 | 25,044 | 14.88% | 42.44 (40.51 to 44.35) |
| Digestive | Liver | Alcoholic liver disease | K70 | | 13,794 | 8,010 | 5,784 | 3.44% | 42.28 (38.15 to 46.36) |
|  |  | Fibrosis and cirrhosis | K74 | | 7,614 | 4,972 | 2,642 | 1.57% | 34.89 (28.81 to 40.85) |
|  |  | Other | K71-73; K75-76 | | 2,326 | 2,209 | 117 | 0.07% | 14.63 (5.13 to 25.57) |
|  |  | Total | K70-77 | | 23,786 | 13,533 | 10,253 | 6.09% | 43.1 (40.02 to 46.16) |
|  | Oesophagus, stomach, duodenum |  | K20-31 | | 2,253 | 1,890 | 363 | 0.22% | 16.15 (-0.94 to 32.25) |
|  | Gallbladder, biliary tract, pancreas |  | K80-87 | | 2,757 | 2,277 | 480 | 0.29% | 17.42 (4.32 to 29.91) |
|  | Other |  | K00-14; K35-38, K40-46; K65-66; K90-93 | | 4,726 | 3,393 | 1,333 | 0.79% | 28.19 (19.59 to 36.59) |
|  | Other intestines |  | K50-64 | | 7,417 | 5,380 | 2,037 | 1.21% | 27.47 (20.55 to 34.15) |
|  | TOTAL |  | K00-93 | | 40,990 | 23,810 | 17,180 | 10.21% | 42.2 (39.91 to 44.46) |
| Skin and subcutaneous tissue | TOTAL |  | L00-L99 | | 882 | 828 | 54 | 0.03% | 6.12 (1.8 to 34.05) |
| Musculoskeletal | TOTAL |  | M00-99 | | 3,063 | 2,638 | 425 | 0.25% | 13.86 (10.5 to 28.83) |
| Genitourinary system | Other |  | N00-16; N20-99 | | 3,384 | 2,204 | 1,180 | 0.70% | 34.89 (24.96 to 44.46) |
|  | Renal failure |  | N17-19 | | 3,704 | 2,811 | 893 | 0.53% | 24.11 (13.68 to 34.21) |
|  | TOTAL |  | N00-99 | | 7,093 | 4,482 | 2,611 | 1.55% | 37.78 (31.77 to 43.67) |
| Symptoms, signs,  abnormal findings | TOTAL |  | R00-99 | | 41,970 | 22,520 | 19,450 | 11.56% | 46.67 (44.48 to 48.85) |
| External | Undetermined intent |  | Y10-34 | | 5,220 | 4,401 | 819 | 0.49% | 15.68 (6.2 to 24.92) |
|  | Accidents | Drugs, alcohol poisoning | X41-42; X44-45 | | 2,154 | 1,665 | 489 | 0.29% | 22.69 (14.25 to 46.31) |
|  |  | Other | X00-40; X43; X46-59; W00-98; V00; V07-08; V37; V45; V57; V68; V80; V83; V86; V90-97 | | 19,592 | 12,252 | 7,340 | 4.36% | 38.1 (34.66 to 41.55) |
|  |  | Vehicle accidents | V01-06; V09-34; V38-44; V46-55;  V58-67; V69-79; V81-82; V84-85; V87-89 | | 17,345 | 14,282 | 3,063 | 1.82% | 18.12 (13.77 to 22.48) |
|  |  | Total | X00-59; V00-97; W00-98 | | 39,203 | 25,980 | 13,223 | 7.86% | 33.72 (31.12 to 36.32) |
|  | Other |  | X85-99; Y00-09; Y35-36; Y85-98 | | 3,706 | 3,384 | 322 | 0.19% | 8.69 (-4.41 to 21.34) |
|  | Intentional self-harm |  | X60-84 | | 34,845 | 26,418 | 8,427 | 5.01% | 24.18 (21.31 to 27.03) |
|  | TOTAL |  | X00-99; V00-97; W00-98; Y00-98 | | 83,000 | 56,164 | 26,836 | 15.94% | 32.38 (30.64 to 34.11) |
| Congenital | TOTAL |  | Q00-99 | | 2,347 | 2,533 | -186 | -0.11% | -7.67 (2.05 to 14.78) |
| Other | TOTAL |  | P00-96; O00-99; H05-82; Z05-ZZ; S18; T32-98; U00-49; U83-85 | | 5,915 | 6,301 | -386 | -0.23% | -7.82 (4.01 to 12.06) |
| ALL-CAUSE | TOTAL |  |  | | 789,666 | 568,560 | 221,106 | 100.00% | 21.31 (20.68 to 21.95) |

## Potential years of life lost in Belgium since 1998

*Table S5 Overview of observed and expected PYLL per 100,000 persons*

|  | 1998-2003 | 2004-2008 | 2009-2013 | 2014-2019 | Absolute  difference | Relative  difference |
| --- | --- | --- | --- | --- | --- | --- |
| men |  |  |  |  |  |  |
| observed PYLL per 100,000 | 8,261 | 7,283 | 6,398 | 5,592 | -2,669 | -32.31% |
| expected PYLL per 100,000 | 5,959 | 5,122 | 4,501 | 3,823 | -2,136 | -35.84% |
| Absolute difference | -2,302 | -2,161 | -1,897 | -1,769 | 533 |  |
| Relative difference | -27.9% | -29.7% | -29.6% | -31.6% |  |  |
| women |  |  |  |  |  |  |
| observed PYLL per 100,000 | 4,242 | 3,914 | 3,631 | 3,336 | -906 | -21.36% |
| expected PYLL per 100,000 | 3,292 | 2,944 | 2,371 | 2,446 | -846 | -25.70% |
| Absolute difference | -950 | -970 | -1,260 | -890 |  |  |
| Relative difference | -22.4% | -24.8% | -34.7% | -26.7% |  |  |

*Table S6 Overview of potential years of life lost in men in Belgium from 1998 to 2019*

| **Chapter** | **Subgroup** | **Diagnosis** | **ICD10 codes** | **PYLLS** | **% cause-specific PYLL / overall PYLL** | **Expected PYLL** | **Excess  PYLLS** | **% cause-specific Excess PYLL/overall excess PYLL PYLLS** | **% excess cause-specific PYLL/ cause-specific PYLL  PYLLS from PYLLS** |
| --- | --- | --- | --- | --- | --- | --- | --- | --- | --- |
| **Infections** | **Viral hepatitis** |  | B15-B19 | 9,773 | 0.13% | 5,260 | 4,513 | 0.19% | 46.18% |
|  | **Other** |  | A0-B09; B20-B99 | 116,362 | 1.61% | 65,820 | 50,542 | 2.16% | 43.44% |
|  | **TOTAL** |  | A01-99; B00-99 | 126,135 | 1.74% | 71,080 | 55,055 | 2.35% | 43.65% |
| **Neoplasm** | **Respiratory** | **Larynx** | C32 | 40,493 | 0.56% | 23,290 | 17,203 | 0.73% | 42.48% |
|  |  | **Lung** | C34 | 679,646 | 9.38% | 491,830 | 187,816 | 8.01% | 27.63% |
|  |  | **Other** | C30-31;C33;C35-39 | 75,131 | 1.04% | 51,000 | 24,131 | 1.03% | 32.12% |
|  |  | **Total** | C30-39 | 737,105 | 10.18% | 529,520 | 207,585 | 8.86% | 28.16% |
|  | **Mouth** |  | C00-14 | 106,156 | 1.47% | 68,950 | 37,206 | 1.59% | 35.05% |
|  | **Other** |  | C76-80; C97;D00-D48 | 177,038 | 2.44% | 106,070 | 70,968 | 3.03% | 40.09% |
|  | **Urinary** | **Bladder** | C67 | 47,577 | 0.66% | 37,030 | 10,547 | 0.45% | 22.17% |
|  |  | **Kidney** | C64 | 45,238 | 0.62% | 43,720 | 1,518 | 0.06% | 3.36% |
|  |  | **Other** | C65-66; C68 | 9,734 | 0.13% | 6,710 | 3,024 | 0.13% | 31.07% |
|  |  | **Total** | C64-68 | 102,549 | 1.42% | 87,460 | 15,089 | 0.64% | 14.71% |
|  | **Digestive** | **Colon** | C18 | 120,303 | 1.66% | 113,940 | 6,363 | 0.27% | 5.29% |
|  |  | **Liver & IH bile ducts** | C22 | 70,937 | 0.98% | 44,620 | 26,317 | 1.12% | 37.10% |
|  |  | **Oesophagus** | C15 | 97,258 | 1.34% | 81,810 | 15,448 | 0.66% | 15.88% |
|  |  | **Other** | C17;C21;C23;C24;C26 | 75,240 | 1.04% | 54,450 | 20,790 | 0.89% | 27.63% |
|  |  | **Pancreas** | C25 | 106,081 | 1.46% | 94,560 | 11,521 | 0.49% | 10.86% |
|  |  | **Rectosigmoid junction** | C19 | 8,521 | 0.12% | 6,710 | 1,811 | 0.08% | 21.25% |
|  |  | **Rectum** | C20 | 37,525 | 0.52% | 34,510 | 3,015 | 0.13% | 8.03% |
|  |  | **Stomach** | C16 | 68,265 | 0.94% | 52,590 | 15,675 | 0.67% | 22.96% |
|  |  | **Total** | C15-26 | 542,458 | 7.49% | 460,220 | 82,238 | 3.51% | 15.16% |
|  | **Thyroid & endocrine glands** |  | C73-75 | 12,819 | 0.18% | 14,920 | -2,101 | -0.09% | -16.39% |
|  | **Male genital organs** |  | C60-63 | 72,507 | 1.00% | 62,010 | 10,497 | 0.45% | 14.48% |
|  | **Lymphoid & haematopoietic** | **Leukaemia, unspecified unspecified** | C95 | 15,854 | 0.22% | 12,700 | 3,154 | 0.13% | 19.89% |
|  |  | **Lymphoid leukaemia** | C91 | 19,247 | 0.27% | 17,740 | 1,507 | 0.06% | 7.83% |
|  |  | **Multiple myeloma** | C90 | 22,065 | 0.30% | 21,500 | 565 | 0.02% | 2.56% |
|  |  | **Myeloid leukaemia** | C92 | 35,857 | 0.50% | 28,160 | 7,697 | 0.33% | 21.47% |
|  |  | **Other** | C81-89;C93-94;C96 | 61,909 | 0.85% | 59,050 | 2,859 | 0.12% | 4.62% |
|  |  | **Total** | C81-96 | 154,932 | 2.14% | 139,150 | 15,782 | 0.67% | 10.19% |
|  | **Bone and articular cartilage** |  | C40-41 | 19,334 | 0.27% | 20,070 | -736 | -0.03% | -3.81% |
|  | **Mesothelial and soft tissue** |  | C45-49 | 42,959 | 0.59% | 45,690 | -2,731 | -0.12% | -6.36% |
|  | **Breast** |  | C50 | 2,448 | 0.03% | 2,490 | -42 | 0.00% | -1.72% |
|  | **Eye, brain & CNS** |  | C69-72 | 118,506 | 1.64% | 129,590 | -11,084 | -0.47% | -9.35% |
|  | **Skin** |  | C43-44 | 43,624 | 0.60% | 51,020 | -7,396 | -0.32% | -16.95% |
|  | **TOTAL** |  | C00-97;D00-48 | 2,132,435 | 29.44% | 1,717,160 | 415,275 | 17.72% | 19.47% |
| **Blood** | **TOTAL** |  | D50-89 | 18,346 | 0.25% | 9,530 | 8,816 | 0.38% | 48.05% |
| **Endocrine, nutritional, metabolic** | **Diabetes mellitus** |  | E10-14 | 72,062 | 0.99% | 34,040 | 38,022 | 1.62% | 52.76% |
|  | **Metabolic disorders** |  | E70-89 | 34,953 | 0.48% | 18,250 | 16,703 | 0.71% | 47.79% |
|  | **Obesity** |  | E66-68 | 15,458 | 0.21% | 5,730 | 9,728 | 0.42% | 62.93% |
|  | **Other** |  | E00-07;E15-63 | 7,312 | 0.10% | 3,260 | 4,052 | 0.17% | 55.42% |
|  | **TOTAL** |  | E00-E89 | 129,785 | 1.79% | 61,280 | 68,505 | 2.92% | 52.78% |
| **Mental behavioural** | **Psychoactive substance use** | **Alcohol** | F10 | 90,417 | 1.25% | 31,850 | 58,567 | 2.50% | 64.77% |
|  |  | **Other** | F11-19 | 33,812 | 0.47% | 7,400 | 26,412 | 1.13% | 78.11% |
|  |  | **Total** | F10-19 | 124,229 | 1.72% | 39,250 | 84,979 | 3.63% | 68.41% |
|  | **Organic** |  | F01-F09 | 13,002 | 0.18% | 9,760 | 3,242 | 0.14% | 24.93% |
|  | **Other** |  | F20-99 | 20,456 | 0.28% | 9,120 | 11,336 | 0.48% | 55.42% |
|  | **TOTAL** |  | F01-99 | 157,687 | 2.18% | 58,130 | 99,557 | 4.25% | 63.14% |
| **Nervous** | **Episodic & paroxysmal** |  | G40-47 | 48,640 | 0.67% | 22,590 | 26,050 | 1.11% | 53.56% |
|  | **Other** |  | G00-09;G50-64; G70-72;  G80--83; G90-98 | 68,493 | 0.95% | 42,470 | 26,023 | 1.11% | 37.99% |
|  | **Other degenerative** |  | G30-31 | 19,628 | 0.27% | 17,200 | 2,428 | 0.10% | 12.37% |
|  | **Demyelinating diseases of CNS** |  | G35-37 | 14,422 | 0.20% | 14,390 | 32 | 0.00% | 0.22% |
|  | **Extrapyramidal & movement** |  | G20-26 | 12,089 | 0.17% | 11,130 | 959 | 0.04% | 7.93% |
|  | **Systemic atrophies of CNS** |  | G10-14 | 37,486 | 0.52% | 41,280 | -3,794 | -0.16% | -10.12% |
|  | **TOTAL** |  | G00-98 | 200,758 | 2.77% | 149,060 | 51,698 | 2.21% | 25.75% |
| **Circulatory** |  |  |  |  |  |  |  |  |  |
|  | **Hypertensive** |  | I10-15 | 22,621 | 0.31% | 10240 | 12,381 | 0.53% | 54.73% |
|  | **Ischaemic heart disease** | **Acute MI** | I21 | 429,595 | 5.93% | 310,740 | 118,855 | 5.07% | 27.67% |
|  |  | **Chronic** | I25 | 132,713 | 1.83% | 94,130 | 38,583 | 1.65% | 29.07% |
|  |  | **Other** | I20;I22-24 | 23,120 | 0.32% | 16,150 | 6,970 | 0.30% | 30.15% |
|  |  | **Total** | I20-25 | 585,428 | 8.08% | 421,020 | 164,408 | 7.01% | 28.08% |
|  | **Pulmonary** | **Other** | I27-28 | 4,818 | 0.07% | 3,210 | 1,608 | 0.07% | 33.37% |
|  |  | **Pulmonary embolism** | I26 | 38,598 | 0.53% | 23,370 | 15,228 | 0.65% | 39.45% |
|  |  | **Total** | I26-28 | 43,416 | 0.60% | 26,580 | 16,836 | 0.72% | 38.78% |
|  | **Veins, lymph, vessels and nodes** |  | I80-89 | 12,514 | 0.17% | 5,180 | 7,334 | 0.31% | 58.61% |
|  | **Other types of heart disease** | **Atrial fibrillation & flutter** | I48 | 10,753 | 0.15% | 7,760 | 2,993 | 0.13% | 27.83% |
|  |  | **Cardiomyopathy** | I42 | 34,269 | 0.47% | 27,230 | 7,039 | 0.30% | 20.54% |
|  |  | **Heart failure** | I50 | 70,411 | 0.97% | 37,480 | 32,931 | 1.41% | 46.77% |
|  |  | **Other** | I30-40; I43-47; I49; I51 | 227,515 | 3.14% | 172,750 | 54,765 | 2.34% | 24.07% |
|  |  | **Total** | I30-51 | 342,948 | 4.73% | 245,220 | 97,728 | 4.17% | 28.50% |
|  | **Stroke** | **Haemorrhage** | I60-62 | 107,065 | 1.48% | 76,230 | 30,835 | 1.32% | 28.80% |
|  |  | **Infarction** | I63 | 26,200 | 0.36% | 19,650 | 6,550 | 0.28% | 25.00% |
|  |  | **Other** | I64-69 | 72,571 | 1.00% | 49,030 | 23,541 | 1.00% | 32.44% |
|  |  | **Total** | I60-69 | 205,836 | 2.84% | 144,910 | 60,926 | 2.60% | 29.60% |
|  | **Arteries** | **Aortic aneurysm & dis.** | I71 | 35,765 | 0.49% | 31,120 | 4,645 | 0.20% | 12.99% |
|  |  | **Other** | I70; I72-79 | 31,631 | 0.44% | 16,540 | 15,091 | 0.64% | 47.71% |
|  |  | **Total** | I70-79 | 67,396 | 0.93% | 47,660 | 19,736 | 0.84% | 29.28% |
|  | **Other** |  | I00-09; I95-99 | 7,415 | 0.10% | 4,780 | 2,635 | 0.11% | 35.54% |
|  | **TOTAL** |  | I00-99 | 1,287,574 | 17.78% | 905,590 | 381,984 | 16.30% | 29.67% |
| **Respiratory** | **Chronic lung disease** | **Asthma** | J45 | 11,398 | 0.16% | 3,170 | 8,228 | 0.35% | 72.19% |
|  |  | **Other (COPD)** | J40-44; J46-47 | 183,340 | 2.53% | 84,010 | 99,330 | 4.24% | 54.18% |
|  |  | **Total** | J40-47 | 194,738 | 2.69% | 87,180 | 107,558 | 4.59% | 55.23% |
|  | **Influenza & pneumonia** |  | J09-J18 | 91,476 | 1.26% | 54,260 | 37,216 | 1.59% | 40.68% |
|  | **External agents** |  | J60-70 | 21,982 | 0.30% | 13,890 | 8,092 | 0.35% | 36.81% |
|  | **Other** |  | J00-08;J20-22; J30-39; J80-99 | 58,165 | 0.80% | 36,600 | 21,565 | 0.92% | 37.08% |
|  | **TOTAL** |  | J00-99 | 366,361 | 5.06% | 191,930 | 174,431 | 7.44% | 47.61% |
| **Digestive** | **Liver** | **Alcoholic liver disease** | K70 | 168,571 | 2.33% | 82,010 | 86,561 | 3.69% | 51.35% |
|  |  | **Fibrosis and cirrhosis** | K74 | 75,093 | 1.04% | 41,640 | 33,453 | 1.43% | 44.55% |
|  |  | **Other** | K71-73; K75-76 | 21,860 | 0.30% | 12,810 | 9,050 | 0.39% | 41.40% |
|  |  | **Total** | K70-77 | 265,524 | 3.67% | 136,460 | 129,064 | 5.51% | 48.61% |
|  | **Oesophagus, stomach, duodenum** |  | K20-31 | 19,067 | 0.26% | 7,420 | 11,647 | 0.50% | 61.08% |
|  | **Gallbladder, biliary tract, pancreas** |  | K80-87 | 25,901 | 0.36% | 13,720 | 12,181 | 0.52% | 47.03% |
|  | **Other** |  | K00-14; K35-38, K40-46; K65-66; K90-93 | 41,672 | 0.58% | 22,970 | 18,702 | 0.80% | 44.88% |
|  | **Other intestines** |  | K50-64 | 44,282 | 0.61% | 27,300 | 16,982 | 0.72% | 38.35% |
|  | **TOTAL** |  | K00-93 | 396,446 | 5.47% | 207,870 | 188,576 | 8.05% | 47.57% |
| **Skin and subcutaneous tissue** | **TOTAL** |  | L00-L99 | 4,652 | 0.06% | 2,890 | 1,762 | 0.08% | 37.88% |
| **Musculoskeletal** | **TOTAL** |  | M00-99 | 18,642 | 0.26% | 11,550 | 7,092 | 0.30% | 38.04% |
| **Genitourinary system** | **Other** |  | N00-16; N20-99 | 17,066 | 0.24% | 8,550 | 8,516 | 0.36% | 49.90% |
|  | **Renal failure** |  | N17-19 | 21,200 | 0.29% | 11,190 | 10,010 | 0.43% | 47.22% |
|  | **TOTAL** |  | N00-99 | 38,266 | 0.53% | 19,740 | 18,526 | 0.79% | 48.41% |
| **Symptoms, signs,  abnormal findings** | **TOTAL** |  | R00-99 | 472,054 | 6.52% | 239,250 | 232,804 | 9.93% | 49.32% |
| **External** | **Undetermined intent** |  | Y10-34 | 101,086 | 1.40% | 53,660 | 47,426 | 2.02% | 46.92% |
|  | **Accidents** | **Drugs, alcohol poisoning** | X41-42; X44-45 | 53,402 | 0.74% | 15,410 | 37,992 | 1.62% | 71.14% |
|  |  | **Other** | X00-40; X43; X46-59; W00-98; V00; V07-08; V37; V45; V57; V68; V80; V83; V86; V90-97 | 278,565 | 3.85% | 155,170 | 123,395 | 5.26% | 44.30% |
|  |  | **Vehicle accidents** | V01-06; V09-34; V38-44; V46-55;  V58-67; V69-79; V81-82; V84-85; V87-89 | 493,848 | 6.82% | 349,480 | 144,368 | 6.16% | 29.23% |
|  |  | **Total** | X00-59; V00-97; W00-98 | 825,815 | 11.40% | 520,060 | 305,755 | 13.05% | 37.02% |
|  | **Other** |  | X85-99; Y00-09; Y35-36; Y85-98 | 64,206 | 0.89% | 24,030 | 40,176 | 1.71% | 62.57% |
|  | **Intentional self-harm** |  | X60-84 | 732,146 | 10.11% | 528,760 | 203,386 | 8.68% | 27.78% |
|  | **TOTAL** |  | X00-99; V00-97; W00-98; Y00-98 | 1,723,253 | 23.79% | 1,126,510 | 596,743 | 25.46% | 34.63% |
| **Congenital** | **TOTAL** |  | Q00-99 | 41,040 | 0.57% | 43,350 | -2,310 | -0.10% | -5.63% |
| **Other** | **TOTAL** |  | P00-96; O00-99; H05-82; Z05-ZZ; S18; T32-98; U00-49; U83-85 | 130,133 | 1.80% | 144,490 | -14,357 | -0.61% | -11.03% |
| **ALL-CAUSE** | **TOTAL** |  |  | 7,243,567 | 100.00% | 4,899,798 | 2,343,769 | 100.00% | 32.36% |

Table S7 Overview of potential years of life lost in women in Belgium from 1998 to 2019

| **Chapter** | **Subgroup** | **Diagnosis** | **ICD10 codes** | **PYLLS** | **% cause-specific PYLL / overall PYLL** | **Expected PYLL** | **Excess  PYLLS** | **% cause-specific Excess PYLL/overall excess PYLL PYLLS** | **% excess cause-specific PYLL/ cause-specific PYLL  PYLLS from PYLLS** |
| --- | --- | --- | --- | --- | --- | --- | --- | --- | --- |
| **Infections** | **Viral hepatitis** |  | B15-B19 | 5,321 | 0.13% | 1,630 | 3,691 | 0.34% | 69.37% |
|  | **Other** |  | A0-B09; B20-B99 | 78,578 | 1.98% | 49,420 | 29,158 | 2.69% | 37.11% |
|  | **TOTAL** |  | A01-99; B00-99 | 83,899 | 2.11% | 51,050 | 32,849 | 3.03% | 39.15% |
| **Neoplasm** | **Respiratory** | **Larynx** | C32 | 5,856 | 0.15% | 2,550 | 3,306 | 0.30% | 56.45% |
|  |  | **Lung** | C34 | 279,913 | 7.04% | 186,000 | 93,913 | 8.65% | 33.55% |
|  |  | **Other** | C30-31;C33;C35-39 | 7,457 | 0.19% | 7,920 | -463 | -0.04% | -6.21% |
|  |  | **Total** | C30-39 | 293,226 | 7.37% | 196,470 | 96,756 | 8.91% | 33.00% |
|  | **Mouth** |  | C00-14 | 26,406 | 0.66% | 16,920 | 9,486 | 0.87% | 35.92% |
|  | **Other** |  | C76-80; C97;D00-D48 | 104,432 | 2.63% | 72,250 | 32,182 | 2.97% | 30.82% |
|  | **Urinary** | **Bladder** | C67 | 13,735 | 0.35% | 11,150 | 2,585 | 0.24% | 18.82% |
|  |  | **Kidney** | C64 | 21,509 | 0.54% | 20,800 | 709 | 0.07% | 3.30% |
|  |  | **Other** | C65-66; C68 | 3,928 | 0.10% | 2,810 | 1,118 | 0.10% | 28.46% |
|  |  | **Total** | C64-68 | 39,172 | 0.98% | 34,760 | 4,412 | 0.41% | 11.26% |
|  | **Digestive** | **Colon** | C18 | 89,814 | 2.26% | 84,590 | 5,224 | 0.48% | 5.82% |
|  |  | **Liver & IH bile ducts** | C22 | 30,119 | 0.76% | 23,910 | 6,209 | 0.57% | 20.61% |
|  |  | **Oesophagus** | C15 | 22,921 | 0.58% | 14,750 | 8,171 | 0.75% | 35.65% |
|  |  | **Other** | C17;C21;C23;C24;C26 | 25,292 | 0.64% | 26,570 | -1,278 | -0.12% | -5.05% |
|  |  | **Pancreas** | C25 | 74,406 | 1.87% | 65,090 | 9,316 | 0.86% | 12.52% |
|  |  | **Rectosigmoid junction** | C19 | 6,582 | 0.17% | 6,210 | 372 | 0.03% | 5.65% |
|  |  | **Rectum** | C20 | 21,840 | 0.55% | 21,260 | 580 | 0.05% | 2.66% |
|  |  | **Stomach** | C16 | 32,064 | 0.81% | 23,300 | 8,764 | 0.81% | 27.33% |
|  |  | **Total** | C15-26 | 303,038 | 7.62% | 265,680 | 37,358 | 3.44% | 12.33% |
|  | **Female genital organs** |  | C51-58 | 186,092 | 4.68% | 161,100 | 24,992 | 2.30% | 13.43% |
|  | **Thyroid & endocrine glands** |  | C73-75 | 11,847 | 0.30% | 10,360 | 1,487 | 0.14% | 12.55% |
|  | **Lymphoid & haematopoietic** | **Leukaemia, unspecified unspecified** | C95 | 12,480 | 0.31% | 13,560 | -1,080 | -0.10% | -8.65% |
|  |  | **Lymphoid leukaemia** | C91 | 11,052 | 0.28% | 11,080 | -28 | 0.00% | -0.25% |
|  |  | **Multiple myeloma** | C90 | 15,610 | 0.39% | 14,640 | 970 | 0.09% | 6.21% |
|  |  | **Myeloid leukaemia** | C92 | 28,805 | 0.72% | 24,960 | 3,845 | 0.35% | 13.35% |
|  |  | **Other** | C81-89;C93-94;C96 | 36,199 | 0.91% | 37,920 | -1,721 | -0.16% | -4.75% |
|  |  | **Total** | C81-96 | 104,146 | 2.62% | 102,160 | 1,986 | 0.18% | 1.91% |
|  | **Bone and articular cartilage** |  | C40-41 | 10,129 | 0.25% | 8,440 | 1,689 | 0.16% | 16.67% |
|  | **Mesothelial and soft tissue** |  | C45-49 | 24,675 | 0.62% | 28,020 | -3,345 | -0.31% | -13.56% |
|  | **Breast** |  | C50 | 410,546 | 10.32% | 423,530 | -12,984 | -1.20% | -3.16% |
|  | **Eye, brain & CNS** |  | C69-72 | 79,045 | 1.99% | 82,640 | -3,595 | -0.33% | -4.55% |
|  | **Skin** |  | C43-44 | 35,044 | 0.88% | 39,970 | -4,926 | -0.45% | -14.06% |
|  | **TOTAL** |  | C00-97;D00-48 | 1,627,798 | 40.92% | 1,442,300 | 185,498 | 17.09% | 11.40% |
| **Blood** | **TOTAL** |  | D50-89 | 13,860 | 0.35% | 9,250 | 4,610 | 0.42% | 33.26% |
| **Endocrine, nutritional, metabolic** | **Diabetes mellitus** |  | E10-14 | 37,639 | 0.95% | 16,380 | 21,259 | 1.96% | 56.48% |
|  | **Metabolic disorders** |  | E70-89 | 26,072 | 0.66% | 22,980 | 3,092 | 0.28% | 11.86% |
|  | **Obesity** |  | E66-68 | 13,785 | 0.35% | 4,840 | 8,945 | 0.82% | 64.89% |
|  | **Other** |  | E00-07;E15-63 | 7,094 | 0.18% | 3,360 | 3,734 | 0.34% | 52.64% |
|  | **TOTAL** |  | E00-E89 | 84,590 | 2.13% | 47,560 | 37,030 | 3.41% | 43.78% |
| **Mental behavioural** | **Psychoactive substance use** | **Alcohol** | F10 | 28,302 | 0.71% | 7,530 | 20,772 | 1.91% | 73.39% |
|  |  | **Other** | F11-19 | 7,672 | 0.19% | 2,500 | 5,172 | 0.48% | 67.41% |
|  |  | **Total** | F10-19 | 35,974 | 0.90% | 10,030 | 25,944 | 2.39% | 72.12% |
|  | **Organic** |  | F01-F09 | 10,918 | 0.27% | 7,720 | 3,198 | 0.29% | 29.29% |
|  | **Other** |  | F20-99 | 21,070 | 0.53% | 13,920 | 7,150 | 0.66% | 33.93% |
|  | **TOTAL** |  | F01-99 | 67,962 | 1.71% | 31,670 | 36,292 | 3.34% | 53.40% |
| **Nervous** | **Episodic & paroxysmal** |  | G40-47 | 10,918 | 0.27% | 7,720 | 3,198 | 0.29% | 29.29% |
|  | **Other** |  | G00-09;G50-64; G70-72;  G80--83; G90-98 | 43,001 | 1.08% | 25,330 | 17,671 | 1.63% | 41.09% |
|  | **Other degenerative** |  | G30-31 | 19,114 | 0.48% | 16,580 | 2,534 | 0.23% | 13.26% |
|  | **Demyelinating diseases of CNS** |  | G35-37 | 17,637 | 0.44% | 16,140 | 1,497 | 0.14% | 8.49% |
|  | **Extrapyramidal & movement** |  | G20-26 | 7,891 | 0.20% | 6,580 | 1,311 | 0.12% | 16.61% |
|  | **Systemic atrophies of CNS** |  | G10-14 | 26,584 | 0.67% | 23,530 | 3,054 | 0.28% | 11.49% |
|  | **TOTAL** |  | G00-98 | 139,701 | 3.51% | 109,010 | 30,691 | 2.83% | 21.97% |
| **Circulatory** | **Hypertensive** | **Hypertensive heart disease** | I11 | 3,882 | 0.10% | 1,340 | 2,542 | 0.23% | 65.48% |
|  | **Hypertensive** | **Total** | I10-15 | 10,242 | 0.26% | 4,340 | 5,902 | 0.54% | 57.63% |
|  | **Ischaemic heart disease** | **Acute MI** | I21 | 124,129 | 3.12% | 78,000 | 46,129 | 4.25% | 37.16% |
|  |  | **Chronic** | I25 | 37,896 | 0.95% | 20,030 | 17,866 | 1.65% | 47.14% |
|  |  | **Other** | I20;I22-24 | 7,103 | 0.18% | 5,210 | 1,893 | 0.17% | 26.65% |
|  |  | **Total** | I20-25 | 169,128 | 4.25% | 103,240 | 65,888 | 6.07% | 38.96% |
|  | **Pulmonary** | **Other** | I27-28 | 5,796 | 0.15% | 4,560 | 1,236 | 0.11% | 21.33% |
|  |  | **Pulmonary embolism** | I26 | 36,659 | 0.92% | 22,880 | 13,779 | 1.27% | 37.59% |
|  |  | **Total** | I26-28 | 42,455 | 1.07% | 27,440 | 15,015 | 1.38% | 35.37% |
|  | **Veins, lymph, vessels and nodes** |  | I80-89 | 9,168 | 0.23% | 5,520 | 3,648 | 0.34% | 39.79% |
|  | **Other types of heart disease** | **Atrial fibrillation & flutter** | I48 | 6,550 | 0.16% | 4,480 | 2,070 | 0.19% | 31.60% |
|  |  | **Cardiomyopathy** | I42 | 13,013 | 0.33% | 9,680 | 3,333 | 0.31% | 25.61% |
|  |  | **Heart failure** | I50 | 35,795 | 0.90% | 21,180 | 14,615 | 1.35% | 40.83% |
|  |  | **Other** | I30-40; I43-47; I49; I51 | 105,450 | 2.65% | 71,230 | 34,220 | 3.15% | 32.45% |
|  |  | **Total** | I30-51 | 160,808 | 4.04% | 106,570 | 54,238 | 5.00% | 33.73% |
|  | **Stroke** | **Haemorrhage** | I60-62 | 96,576 | 2.43% | 69,160 | 27,416 | 2.53% | 28.39% |
|  |  | **Infarction** | I63 | 18,782 | 0.47% | 14,340 | 4,442 | 0.41% | 23.65% |
|  |  | **Other** | I64-69 | 44,725 | 1.12% | 32,520 | 12,205 | 1.12% | 27.29% |
|  |  | **Total** | I60-69 | 160,083 | 4.02% | 116,020 | 44,063 | 4.06% | 27.53% |
|  | **Arteries** | **Aortic aneurysm & dis.** | I71 | 9,147 | 0.23% | 6,140 | 3,007 | 0.28% | 32.87% |
|  |  | **Other** | I70; I72-79 | 15,531 | 0.39% | 6,230 | 9,301 | 0.86% | 59.89% |
|  |  | **Total** | I70-79 | 24,678 | 0.62% | 12,370 | 12,308 | 1.13% | 49.87% |
|  | **Other** |  | I00-09; I95-99 | 6,677 | 0.17% | 2,670 | 4,007 | 0.37% | 60.01% |
|  | **TOTAL** |  | I00-99 | 587,121 | 14.76% | 379,510 | 207,611 | 19.13% | 35.36% |
| **Respiratory** | **Chronic lung disease** | **Asthma** | J45 | 10,589 | 0.27% | 2,930 | 7,659 | 0.71% | 72.33% |
|  |  | **Other (COPD)** | J40-44; J46-47 | 99,146 | 2.49% | 38,480 | 60,666 | 5.59% | 61.19% |
|  |  | **Total** | J40-47 | 109,735 | 2.76% | 41,410 | 68,325 | 6.29% | 62.26% |
|  | **Influenza & pneumonia** |  | J09-J18 | 51,106 | 1.28% | 29,360 | 21,746 | 2.00% | 42.55% |
|  | **External agents** |  | J60-70 | 10,160 | 0.26% | 6,150 | 4,010 | 0.37% | 39.47% |
|  | **Other** |  | J00-08;J20-22; J30-39; J80-99 | 34,979 | 0.88% | 17,670 | 17,309 | 1.59% | 49.48% |
|  | **TOTAL** |  | J00-99 | 205,980 | 5.18% | 94,590 | 111,390 | 10.26% | 54.08% |
| **Digestive** | **Liver** | **Alcoholic liver disease** | K70 | 80,343 | 2.02% | 36,410 | 43,933 | 4.05% | 54.68% |
|  |  | **Fibrosis and cirrhosis** | K74 | 36,745 | 0.92% | 15,810 | 20,935 | 1.93% | 56.97% |
|  |  | **Other** | K71-73; K75-76 | 13,988 | 0.35% | 6,980 | 7,008 | 0.65% | 50.10% |
|  |  | **Total** | K70-77 | 131,076 | 3.29% | 59,200 | 71,876 | 6.62% | 54.84% |
|  | **Oesophagus, stomach, duodenum** |  | K20-31 | 9,292 | 0.23% | 5,910 | 3,382 | 0.31% | 36.40% |
|  | **Gallbladder, biliary tract, pancreas** |  | K80-87 | 11,822 | 0.30% | 5,920 | 5,902 | 0.54% | 49.92% |
|  | **Other** |  | K00-14; K35-38, K40-46; K65-66; K90-93 | 19,716 | 0.50% | 11,920 | 7,796 | 0.72% | 39.54% |
|  | **Other intestines** |  | K50-64 | 32,847 | 0.83% | 19,910 | 12,937 | 1.19% | 39.39% |
|  | **TOTAL** |  | K00-93 | 204,753 | 5.15% | 102,860 | 101,893 | 9.39% | 49.76% |
| **Skin and subcutaneous tissue** | **TOTAL** |  | L00-L99 | 3,739 | 0.09% | 1,920 | 1,819 | 0.17% | 48.65% |
| **Musculoskeletal** | **TOTAL** |  | M00-99 | 20,357 | 0.51% | 15,900 | 4,457 | 0.41% | 21.89% |
| **Genitourinary system** | **Other** |  | N00-16; N20-99 | 16,035 | 0.40% | 7,850 | 8,185 | 0.75% | 51.04% |
|  | **Renal failure** |  | N17-19 | 13,824 | 0.35% | 7,110 | 6,714 | 0.62% | 48.57% |
|  | **TOTAL** |  | N00-99 | 29,859 | 0.75% | 14,960 | 14,899 | 1.37% | 49.90% |
| **Symptoms, signs, abnormal findings** | **TOTAL** |  | R00-99 | 213,491 | 5.37% | 102,080 | 111,411 | 10.26% | 52.19% |
| **External** | **Undetermined intent** |  | Y10-34 | 42,103 | 1.06% | 29,850 | 12,253 | 1.13% | 29.10% |
|  | **Accidents** | **Drugs, alcohol poisoning** | X41-42; X44-45 | 17,922 | 0.45% | 5,210 | 12,712 | 1.17% | 70.93% |
|  |  | **Other** | X00-40; X43; X46-59; W00-98; V00; V07-08; V37; V45; V57; V68; V80; V83; V86; V90-97 | 110,327 | 2.77% | 53,620 | 56,707 | 5.22% | 51.40% |
|  |  | **Vehicle accidents** | V01-06; V09-34; V38-44; V46-55;  V58-67; V69-79; V81-82; V84-85; V87-89 | 138,432 | 3.48% | 96,810 | 41,622 | 3.83% | 30.07% |
|  |  | **Total** | X00-59; V00-97; W00-98 | 266,681 | 6.70% | 155,640 | 111,041 | 10.23% | 41.64% |
|  | **Other** |  | X85-99; Y00-09; Y35-36; Y85-98 | 40,798 | 1.03% | 28,280 | 12,518 | 1.15% | 30.68% |
|  | **Intentional self-harm** |  | X60-84 | 256,852 | 6.46% | 182,380 | 74,472 | 6.86% | 28.99% |
|  | **TOTAL** |  | X00-99; V00-97; W00-98; Y00-98 | 606,434 | 15.24% | 396,150 | 210,284 | 19.37% | 34.68% |
| **Congenital** | **TOTAL** |  | Q00-99 | 36,973 | 0.93% | 36,460 | 513 | 0.05% | 1.39% |
| **Other** | **TOTAL** |  | P00-96; O00-99; H05-82; Z05-ZZ; S18; T32-98; U00-49; U83-85 | 51,757 | 1.30% | 58,340 | -6,583 | -0.61% | -12.72% |
| **ALL-CAUSE** | **TOTAL** |  |  | 3,978,274 | 100.00% | 2,892,879 | 1,085,395 | 100.00% | 27.28% |

1. Boyle, P. and D.M. Parkin, *Statistical Methods for Registries*, in *Cancer Registration: Principles and Methods*, O.M. Jensen, et al., Editors. 1991, International Agency for Research on Cancer: Oxford. p. 126-158.

2. Gardner, J.W. and J.S. Sanborn, *Years of potential life lost (YPLL)--what does it measure?* Epidemiology, 1990. **1**(4): p. 322-9.
